# FEMPAR: An Object-Oriented Parallel Finite Element Framework

**DOI:** 10.1007/s11831-017-9244-1

**Published:** 2017-10-11

**Authors:** Santiago Badia, Alberto F. Martín, Javier Principe

**Affiliations:** 1 Department of Civil and Environmental Engineering, Universitat Politècnica de Catalunya, Jordi Girona 1-3, Edifici C1, 08034 Barcelona, Spain; 2 CIMNE Centre Internacional de Mètodes Numèrics en Enginyeria, Parc Mediterrani de la Tecnologia, UPC, Esteve Terradas 5, 08860 Castelldefels, Spain; 3 Department of Fluid Mechanics, Universitat Politècnica de Catalunya, Eduard Maristany, 10-14, 08019 Barcelona, Spain

## Abstract

FEMPAR is an open source object oriented Fortran200X scientific software library for the high-performance scalable simulation of complex multiphysics problems governed by partial differential equations at large scales, by exploiting state-of-the-art supercomputing resources. It is a highly modularized, flexible, and extensible library, that provides a set of modules that can be combined to carry out the different steps of the simulation pipeline. FEMPAR includes a rich set of algorithms for the discretization step, namely (arbitrary-order) grad, div, and curl-conforming finite element methods, discontinuous Galerkin methods, B-splines, and unfitted finite element techniques on cut cells, combined with *h*-adaptivity. The linear solver module relies on state-of-the-art bulk-asynchronous implementations of multilevel domain decomposition solvers for the different discretization alternatives and block-preconditioning techniques for multiphysics problems. FEMPAR is a framework that provides users with out-of-the-box state-of-the-art discretization techniques and highly scalable solvers for the simulation of complex applications, hiding the dramatic complexity of the underlying algorithms. But it is also a framework for researchers that want to experience with new algorithms and solvers, by providing a highly extensible framework. In this work, the first one in a series of articles about FEMPAR, we provide a detailed introduction to the software abstractions used in the discretization module and the related geometrical module. We also provide some ingredients about the assembly of linear systems arising from finite element discretizations, but the software design of complex scalable multilevel solvers is postponed to a subsequent work.

## Introduction

Even though the origins of the FE method trace back to the 50s, the field has drastically evolved during the last six decades, leading to increasingly complex algorithms to improve accuracy, stability, and performance. The use of the *p*-version of the FE method and its exponential convergence makes high-order approximations an excellent option in many applications [1]. Adaptive mesh refinement driven by *a posteriori* error estimates, i.e., *h*-adaptivity, is an essential ingredient to reduce computational cost in an automatic way [2]. For smooth solutions, *p*-adaptivity or hybrid *hp*-adaptivity can further reduce computational cost for a target level of accuracy [3]. Originally, FE methods were restricted to nodal Lagrangian bases for structural problems. The extension of FE methods to other applications, like porous media flow or electromagnetism, motivated the design of more complex bases and require different mappings from the reference to the physical space, complicating the implementation of these techniques in standard FE codes. Saddle-point problems also require particular mixed FE discretizations for stability purposes [4, 5]. More recently, novel FE formulations have been proposed within the frame of exterior calculus, e.g., for mixed linear elasticity problems [6]. Physics-compatible discretization are also gaining attention, e.g., in the field of incompressible fluid mechanics. Divergence-free mixed FEs satisfy mass conservation up to machine precision, but their implementation is certainly challenging [7]. During the last decade, a huge part of the computational mechanics community has embraced isogeometric analysis techniques [8], in which the discretization spaces are defined in terms of NURBS (or simply splines), leading to smoother global spaces. In the opposite direction, discontinuous galerkin (DG) methods have also been actively developed, and novel approaches, like hybridizable DG and Petrov-Galerkin DG methods, have been proposed [9, 10]. As the discretization methods become more and more complex, the efficient implementation of these techniques is more complicated. It also poses a challenge in the design of scientific software libraries, which should be extensible and provide a framework for the (easy) implementation of novel techniques, to be resilient to new algorithmic trends.

The hardware in which scientific codes run evolves even faster. During 40 years, core performance has been steadily increasing, as predicted by Moore’s law. In some years, supercomputers will reach 1 exaflop/s, a dramatic improvement in computational power that will not only affect the extreme scale machines but radically transform the whole range of platforms, from desktops to high performance computing (HPC) clouds. The ability to efficiently exploit the forthcoming 100x boost of computational performance will have a tremendous impact on scientific discoveries/economic benefits based on computational science, reaching almost every field of research. However, all the foreseen exascale growth in computational power will be delivered by increasing hardware parallelism (in distinct forms), and the efficient exploitation of these resources will not be a simple task. HPC architectures will combine general-purpose fat cores, fine-grain many-cores accelerators (GPUs, DSPs, FPGAs, Intel MIC, etc.), and multiple-level disruptive-technology memories, with high non-uniformity as common denominator [11]. This (inevitable) trend challenges algorithm/software design. Traditional bulk-synchronous message passing interface (MPI) approaches are likely to face significant performance obstacles. Significant progress is already being made by MPI+X [12] (with X=OpenMP, CUDA, OpenCL, OmpSs, Kokkos, etc.) hybrid execution models. Going a step further, asynchronous many-task execution models (e.g., Charm++[13], Legion [14], or HPX [15]) and their supporting run-time systems hold great promise [16].

Traditionally, researchers in the field of scientific computing used to develop codes with a very reduced number of developers, e.g., a university department, and a limited life span. The software engineering behind scientific codes was poor. Codes were rigid and non-extensible, and developed for a target application and a specific numerical method. However, the increasing levels of complexity both in terms of algorithms and hardware make the development of scientific software that can efficiently run state-of-the-art numerical algorithms on HPC resources a real challenge. Considering to start from scratch a project of this kind has an ever increasing level of complexity. Furthermore, due to the huge resources required to carry out such a project, it is natural to develop a framework that will be resilient to new algorithmic and hardware trends, in order to maximize life time, and to be applicable to a broad range of applications. In this sense, object-oriented (OO) programming, which provides modularity of codes and data-hiding, is the key for the software design of flexible and scalable (in terms of developers) projects.

There is a number of open source OO FE libraries available through the Internet, e.g., deal.II [17, 18], FEniCS [19], GRINS [20], Nektar++ [21], MOOSE [22], MFEM [23], FreeFem++ [24], and DUNE [25]. In general, these libraries aim to provide all the machinery required to simulate complex problems governed by partial differential equations (PDE) using FE techniques. In any case, every library has its main goal and distinctive features. Some libraries, like FreeFem++ or FEniCS, have extremely simple user interfaces. FEniCS has its own domain specific language for weak forms to automatically generate the corresponding FE code (preventing *p*-adaptivity) and includes a collection of Python wrappers to provide user-friendly access to the services of the library. Other sophisticated libraries like deal.II or DUNE have a slightly more demanding learning curve. In general, parallel adaptivity is at most partially supported; as far as we know, none of the libraries above have support for parallel *hp*-adaptivity, unless DG methods are being used. Some libraries are restricted to a particular cell topology, e.g., deal.II is limited to hexahedral/quadrilateral (n-cubes) meshes, while FEniCS only supports simulations on triangular/tetrahedral (n-simplices) meshes.

In general, these libraries provide modules for some of the different steps in the simulation pipeline, which involves the set-up of the mesh, the construction of the FE space, the integration and assembly of the weak form, the solution of the resulting linear system, and the visualization of the computed solution. The solution of the linear system is clearly segregated from the discretization step in all the scientific software libraries described above (for parallel computations); the linear system is transferred to a general-purpose sparse linear algebra library, mainly PETSc [26–28], Hypre [29], and Trilinos [30, 31]. As a result, the coupling between the discretization step and the linear solver step is somehow weak, since they rely on general purpose solvers, which usually involve simple interfaces. The strong point of these general purpose numerical linear algebra libraries is to be problem-independent, but it also limits their performance for specific applications, since they cannot fully exploit the underlying properties of the PDE operator and the numerical discretization.^1^ This segregation has a clear impact on the type of methods to be used. This black-box approach to general-purpose linear solvers has favoured the use of algebraic multigrid methods, the *de facto* linear solver [29]. On the other hand, geometric multigrid methods and domain decomposition (DD) methods, which are very specific to mesh-based PDE solvers, are not common, even though they can be superior to algebraic methods in many cases. A geometric multigrid method that exploits the *hp*-adaptive structure of the FE space is included in deal.II, but it can only be used in the serial case. In parallel scenarios, DD methods have certainly evolved during the last decade. Modern DD methods do not (necessarily) rely on a static condensation of the internal variables, which requires sparse direct methods for the local subdomain problems. Instead, *inexact* solvers can be used, e.g., multigrid methods, and linear complexity DD preconditioners can be defined (see [33, 34]). The definition of two-level DD methods resembles the one of FE methods, by exchanging the FE and subdomain concepts, and their definition is strongly related to the one of multiscale FEs [35]. Furthermore, multilevel extensions can be naturally defined. In short, state-of-the-art multilevel DD methods can be understood (in their *inexact* version) as a non-conforming multigrid method. Even though the mathematical theory of the DD methods is very sound, high performance implementations are quite recent (see [36–38]). On the other hand, we are not aware of any general purpose FE code that integrates a DD algorithm in the solution workflow. DD methods require sub-assembled matrices to be used, and are not supported by the majority of the existing advanced OO FE libraries. Analogously, the use of block-preconditioning is in general poorly supported, because it involves the discretization of additional operators to define the approximated Schur complement, and the corresponding block-based assembly of matrices.

On the other hand, based on the supercomputing trends, the segregation between time discretization, linearization, space discretization, and linear system solve, will progressively blur. As an example, nonlinear preconditioning and parallel-in-time solvers are two natural ways to attain the higher levels of concurrency of the forthcoming exascale supercomputers [36, 39]. These facts will complicate even more the rigid workflow of current advanced FE libraries. In this sense, current efforts in PETSc to provide nonlinear preconditioning interfaces can be found in [40], relying on call-back functions, and the XBraid solver [41] aims to provide time-parallelism in a non-intrusive way.

## The FEMPAR Project

In this work, we present FEMPAR, an OO FE framework for the solution of PDEs, designed from inception to be highly scalable on supercomputers and to easily handle complex multiphysics problems. The first public release of FEMPAR has almost 300K lines of code written in (mostly) OO Fortran and makes intensive use of the features defined in the 2003 and 2008 standards of the language. The source code that is complementary to this work corresponds to the first public release of FEMPAR, i.e., version 1.0.0. It is available at a git repository [42]. In particular, the first public release was assigned the git tag FEMPAR-1.0.0, in accordance with the “Semantic Versioning” system.^2^

FEMPAR is very rich in terms of FE technology. In particular, it includes not only Lagrangian FEs, but also curl- and div-conforming ones, e.g., Nédélec (edge) and Raviart-Thomas FEs. The library supports n-cube and n-simplex meshes, and arbitrary high-order bases for all the FEs included. Continuous and discontinuous spaces can be used, providing all the machinery for the integration of DG facet (i.e., edges in 2D and faces in 3D) terms. Recently, in a beta version of the code, B-splines have also been added, together with the support for cut cell methods (using XFEM-type techniques) and *hp*-adaptivity, but we will not discuss these developments for the sake of brevity.

Moreover, FEMPAR has been developed with the aim to provide a framework that will allow developers to implement complex techniques that are not well-suited in the traditional segregated workflow commented above. FEMPAR also provides a highly scalable built-in numerical linear algebra module based on state-of-the-art domain decomposition solvers. FEMPAR can provide partially assembled matrices, required for DD solvers; the multilevel BDDC solver in FEMPAR has scaled up to almost half a million cores and 1.75 million MPI tasks (subdomains) in the JUQUEEN Supercomputer [34, 37]. It includes an abstract framework to construct applications and preconditioners based on multilevel nonoverlapping partitions. Even though every block within the library preserves modularity, the interface between discretization and numerical linear algebra modules within FEMPAR is very rich and focused on PDE-based linear systems. In the path to the exascale, FEMPAR has been designed to permit an asynchronous implementation of multilevel methods, both in terms of multiphysics FEs and multilevel solvers, which have been exploited, e.g., in [37]. It is a unique feature that is not available in other similar libraries. The library also allows the user to define blocks in multiphysics applications, that can be used to easily implement complex block preconditioners [43–45]. All these blocks are very customizable, which has already been used to develop scalable DD solvers for electromagnetics problems and block preconditioners for multiphysics problems, e.g., magnetohydrodynamics [44]. These distinctive features of FEMPAR, however, are not discussed in this article but in a forthcoming one. A general discussion of the main ingredients of our implementation of the discretization step using FE-like approximations is first necessary, which is the purpose of this work.

FEMPAR has already been successfully used in a wide set of applications by the authors of the library: simulation of turbulent flows and stabilized FE methods [46–49], magnetohydrodynamics [50–54], monotonic FEs [55–59], unfitted FEs and embedded boundary methods [60], and additive manufacturing simulations [61]. It has also been used for the highly efficient implementation of DD solvers [34, 37, 39, 62–66] and block preconditioning techniques [44].

This work is more than an overview article with the main features of the library. It is a detailed description of the software abstractions being used within FEMPAR to develop an efficient, modular, and extensible implementation of FE methods and supporting modules in a broad sense. To this end, we enrich the discussion with code snippets that describe data structures, bindings, and examples of use.^3^ This document is intended to be used as a guide for new FEMPAR *developers* that want to get familiarized with its software abstractions. But it can also be a useful tool for developers of FE codes that want to learn how to implement FE methods in an advanced OO framework. In any case, due to the size of the library itself, many details cannot be exposed, to keep a reasonable article length. The article can be read in different ways, since it is not necessary to fully understand all the preceding sections to grasp the main ideas of a section. For instance, the section about the abstract implementation of polytopes in arbitrary dimensions and its related algorithms is quite technical and a reader that is not particularly interested in the internal design of this type and its bindings implementations can skip it. Experienced FE researchers can skip the short section with the basics of FE methods, and only look at this one (if needed) when referred in subsequent sections.

The article is organized as follows. In Sect. 3 we present a concise mathematical description of the FE framework. The main mathematical abstractions are expressed in software by means of a set of derived data types and their associated TBPs, which are described in subsequent sections. In particular, the main software abstractions in FEMPAR and their roles in the solution of the problem are:

- The polytope, which describes a set of admissible geometries and permits the automatic, dimension-independent generation of reference cells and structured domains. The mathematics underlying the polytope are presented in Sect. 3.14, while its software implementation in Sect. 4.
- The polynomial abstraction and related data types, which are presented in Sects. 3.4 and 5, respectively. These sections describe how shape functions bases can be generated for arbitrary orders and for n-cube and n-simplex topologies.
- The reference FE in Sect. 6, which describes the reference cell and defines a set of basis functions and degrees of freedom on each cell.
- The triangulation in Sect. 7, which represents a discrete approximation of the physical domain $\Omega$.
- A set of tools required to perform numerical integration (e.g., quadratures and geometrical maps) produced by the reference FE and described in Sect. 8 for cell integrals and in Sect. 9 for facet integrals.
- The FE space described in Sect. 10, built from a triangulation and a set of reference FEs, which represents a global space of FE functions.
- The discrete integration, an abstract class to be extended by the user to define an affine FE operator, which describes the numerical integration of the weak form of the problem to be solved, described in Sect. 11.2.
- The linear (affine) operator in Sect. 11, whose root is the solution of the problem at hand, constructed using the FE space and a discrete integration.
- An example of a user driver in Sect. 12, in which the different ingredients previously described are used to simulate a problem governed by PDEs, the Stokes system.

**Fig. 1:**
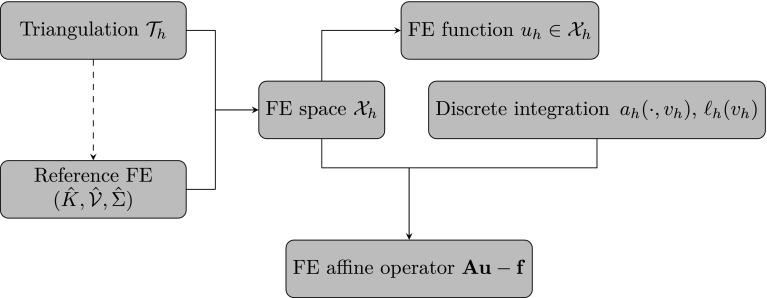
Main software abstractions in FEMPAR and some of their relationships

A (very simplified) graphical overview of the main software abstractions in FEMPAR and some of their relationships is shown in Fig. 1.

## The FE Framework

In this section, we briefly introduce all the mathematical abstractions behind the FE method for the discretization of problems governed by PDEs. For a more detailed exposition of the topics, we refer to [69–72]. The FEs described below (and many other not covered herein) can be formulated and analyzed using the *finite element exterior calculus* framework [6], which makes use of exterior algebra and exterior calculus concepts. In this framework, one can define FEs, e.g., div and curl-conforming ones, in arbitrary space dimensions, using the concept of differential *k*-forms. However, we have decided not to use such presentation of FE methods to simplify the exposition for readers not familiar with these abstractions.

### The Boundary Value Problem in Weak Form

We are interested in problems governed by PDEs posed in a physical domain $\Omega \subset \mathbb {R}^d$ with boundary ${\Gamma }\, \doteq \, \partial \Omega$. In practice $d=2,3$ but we are also interested in $d>3$ for some particular applications (see Sect. 3.14). Let us consider a differential operator ${ A }$, e.g., the Laplace operator $-\Delta$, and a force term $f: \Omega \rightarrow \mathbb {R}$. Let us also consider a partition of ${\Gamma }$ into a Dirichlet boundary ${\Gamma _\mathrm{D}}$ and a Neumann boundary ${\Gamma _\mathrm{N}}$, and the corresponding boundary data $u_\mathrm{D}: {\Gamma _\mathrm{D}}\rightarrow \mathbb {R}$ and $g_\mathrm{N}: {\Gamma _\mathrm{N}}\rightarrow \mathbb {R}$. The boundary value problem reads as follows: find $u(\varvec{x})$ such that

1 $$\begin{aligned} { A }u(\varvec{x}) = f(\varvec{x}) \quad \hbox { in } \, \Omega , \qquad { B }_\mathrm{D} u(\varvec{x}) = u_\mathrm{D}(\varvec{x}) \quad \hbox { on } \, {\Gamma _\mathrm{D}}, \qquad { B }_\mathrm{N} u(\varvec{x}) = g_\mathrm{N}(\varvec{x}) \quad \hbox { on } \, {\Gamma _\mathrm{N}}. \end{aligned}$$

The operator ${ B }_\mathrm{D}$ is a trace operator and ${ B }_\mathrm{N}$ is the flux operator. Other boundary conditions, e.g., Robin (mixed) conditions can also be considered. We assume that the unknown $u(\varvec{x})$ in (1) can be a scalar, vector, or tensor field. (The case of multi-field problems is considered in Sect. 3.11.)

For FE analysis, we must consider the weak form of (1). The weak formulation can be stated in an abstract setting as follows. Let us consider an abstract problem determined by a Banach space $\mathcal {X}$ (*trial space*), a reflexive Banach space $\mathcal {Y}$ (*test space*), a continuous bilinear form ${a} : \mathcal {X}\times \mathcal {Y}\rightarrow \mathbb {R}$, and a continuous linear form $\ell : \mathcal {Y}\rightarrow \mathbb {R}$. The abstract problem is stated as: find $u \in \mathcal {X}$ such that

2 $$\begin{aligned} {a}(u,v) = \ell ( v) , \qquad \hbox { for any } \, v \in \mathcal {Y}. \end{aligned}$$

The link between the two formulations is the following. Let $\mathcal {D}(\Omega )$ be the space of $\mathcal {C}^\infty$ functions with compact support in $\Omega$; the dual space $\mathcal {D}(\Omega )'$ is the space of distributions. We have that:

$$\begin{aligned} {a}(u,\varphi ) \, \doteq \, \langle { A }u, \varphi \rangle _\Omega , \qquad \ell ( \varphi ) \, \doteq \, \langle g_{\mathrm{N}}, \varphi \rangle _{{\Gamma _\mathrm{N}}} + \langle f, \varphi \rangle _\Omega , \quad \hbox { for any } \varphi \in \mathcal {D}(\Omega ), \end{aligned}$$

where the derivatives are understood in distributional sense. e.g., For the Laplace operator, the bilinear form reads ${a}(u,v) \, \doteq \, \int _\Omega {\varvec{\nabla }}u \cdot {\varvec{\nabla }}v \mathrm{d} \Omega$. Furthermore, homogeneous Dirichlet boundary conditions, i.e., $u = 0$ on ${\Gamma _\mathrm{D}}$, are usually enforced in a strong way; the functions in $\mathcal {Y}$ satisfy these boundary conditions. The extension to non-homogeneous boundary conditions is straightforward. One can define an arbitrary extension $Eu_\mathrm{D}$ of the Dirichlet data, i.e., $Eu_\mathrm{D} = u_{\mathrm{D}}$ on ${\Gamma _\mathrm{D}}$. Next, we define the function $u_0 \, \doteq \, u - Eu_\mathrm{D}$ with zero trace on ${\Gamma _\mathrm{D}}$ and solve (2) for ${u}_0$ with the right-hand side

3 $$\begin{aligned} \ell ( v) -{a}(Eu_\mathrm{D},v). \end{aligned}$$

Let us consider two classical examples.

#### *Example 3.1*

(Heat equation) Let us consider the Poisson problem $-{\varvec{\nabla }}\cdot \varvec{\kappa } {\varvec{\nabla }}u = f$ with $u = u_{\mathrm{D}}$ on ${\Gamma _\mathrm{D}}$ and $\partial _{\varvec{n}} u = g_\mathrm{N}$; $\varvec{n}$ is the outward normal. Let us assume that $\varvec{\kappa } \in L^\infty (\Omega )^{d \times d}$, $f \in H^{-1}(\Omega )$, $g_{\mathrm{N}} \in H^{-\frac{1}{2}}({\Gamma _\mathrm{N}})$, and $u_{\mathrm{D}} \in H^\frac{1}{2}({{\Gamma _\mathrm{D}}})$. Let us also consider an extension $Eu_\mathrm{D} \in H^{1}(\Omega )$ such that $Eu_\mathrm{D} = u_{\mathrm{D}}$ on ${\Gamma _\mathrm{D}}$. The weak form of the problem reads as: find $u_0 \in H_0^1(\Omega )$ such that

$$\begin{aligned} \int _\Omega \varvec{\kappa } {\varvec{\nabla }}u_0 \cdot {\varvec{\nabla }}v \mathrm{d}\Omega = \int _\Omega f v \mathrm{d}\Omega + \int _{\Gamma _\mathrm{N}}g v \mathrm{d}\Gamma - \int _\Omega \varvec{\kappa } {\varvec{\nabla }}Eu_\mathrm{D} \cdot {\varvec{\nabla }}v \mathrm{d}\Omega , \qquad \hbox {for any } \, v \in H_0^1(\Omega ). \end{aligned}$$

The solution is $u \, \doteq \, u_0 + Eu_\mathrm{D}$.

#### *Example 3.2*

(Stokes problem) The Stokes problem consists on finding a velocity field $\varvec{u}$ and a pressure field *p* such that

$$\begin{aligned} -{\varvec{\nabla }}\cdot (\mu {\varvec{\epsilon }}( \varvec{u}) ) + {\varvec{\nabla }}p = \varvec{f}, \qquad \qquad {\varvec{\nabla }}\cdot \varvec{u}= 0, \end{aligned}$$

and (for example) $\varvec{u}=\varvec{u}_\mathrm{D}$ on $\Gamma$, where ${\varvec{\epsilon }}(\varvec{u}) = \frac{1}{2}({\varvec{\nabla }}\varvec{u}+ {\varvec{\nabla }}\varvec{u}^T)$ is the strain tensor. The weak form of the problem consists of finding $(\varvec{u}_0,p) \in \mathcal {X}\, \doteq \, \left[ H^1_0(\Omega )\right] ^d \times L_0^2(\Omega )$ such that

$$\begin{aligned} \mu \int _{\Omega } {\varvec{\epsilon }}( \varvec{u}_0) : {\varvec{\epsilon }}( \varvec{v}) - \int _{\Omega } {\varvec{\nabla }}\cdot \varvec{v}p + \int _{\Omega } q {\varvec{\nabla }}\cdot \varvec{u}_0 =\int _{\Omega } \varvec{v}\cdot \varvec{f}- \mu \int _{\Omega } {\varvec{\epsilon }}( \varvec{E}\varvec{u}_\mathrm{D}) : {\varvec{\epsilon }}( \varvec{v}) - \int _{\Omega } q {\varvec{\nabla }}\cdot \varvec{E} \varvec{u}_\mathrm{D}, \end{aligned}$$

for any $(\varvec{v},q) \in \mathcal {X}$, where $\varvec{E}\varvec{u}_\mathrm{D} \in \left[ H^1_0(\Omega )\right] ^d$ is an extension of the Dirichlet data, i.e., $\varvec{E}\varvec{u}_\mathrm{D}=\varvec{u}_\mathrm{D}$ on $\Gamma$. The solution is $\varvec{u}\, \doteq \, \varvec{u}_0+ \varvec{E}\varvec{u}_\mathrm{D}$.

### Space Discretization with FEs

Problem (2) is an infinite-dimensional problem. In order to end up with a computable one, we must introduce finite-dimensional subspaces with some approximability properties. We restrict ourselves to FE schemes in a broad sense that involve conforming and non-conforming spaces. Thus, our aim is to explicitly build spaces $\mathcal {X}_h$ (and $\mathcal {Y}_h$) with some approximability properties. If the discrete spaces are subspaces of the original ones (conforming), i.e., $\mathcal {X}_h\subset \mathcal {X}$ and $\mathcal {Y}_h\subset \mathcal {Y}$, the discrete problem reads as: find $u_h \in \mathcal {X}_h$ such that

$$\begin{aligned} {a}(u_h,v_h) = \ell ( v_h) , \qquad \hbox { for any } \, v_h \in \mathcal {Y}_h. \end{aligned}$$

This is the *Petrov-Galerkin* problem. In the particular case when $\mathcal {X}_h= \mathcal {Y}_h$, we have a *Galerkin* problem. The previous problem can be ill-posed for some choices of the FE spaces, e.g., using discrete spaces that do not satisfy the inf-sup condition for indefinite problems [5]. In some cases, judiciously chosen perturbations of ${a}(\cdot ,\cdot )$ and $\ell ( \cdot )$, represented with ${a_h}(\cdot ,\cdot )$ and $\ell _h( \cdot )$ respectively, can *stabilize* the problem and make it stable and optimally convergent, circumventing the inf-sup condition restriction. In the most general case, we can describe any FE space as: find $u_h \in \mathcal {X}_h$ such that

4 $$\begin{aligned} a_h(u_h,v_h) = \ell _h(v_h), \qquad \hbox { for any } \, v_h \in \mathcal {Y}_h, \end{aligned}$$

replacing the continuous bilinear form by a general *discrete* bilinear form. One can also define the affine operator

5 $$\begin{aligned} \mathcal {F}_h(u_h) = a_h(u_h,\cdot ) - \ell _h(\cdot ) \in \mathcal {Y}_h', \end{aligned}$$

and state (4) as: find $u_h \in \mathcal {X}_h$ such that $\mathcal {F}_h(u_h) = 0$. This statement is the one being used for the practical implementation of FE operators in FEMPAR (see Sect. 11).

In order to define FE spaces, we require a triangulation $\mathcal {T}_h$ of the domain $\Omega$ into a set $\{ K\}$ of *cells*. This triangulation is assumed to be conforming, i.e., for two neighbour cells $K^+, \, K^- \in \mathcal {T}_h$, its intersection $K^+ \cap K^-$ is a *whole* *k*-face ($k<d$) of both cells (note that *k*-face refers to a geometrical entity, e.g. cells, faces, edges and vertices for $d=3$, see Sect. 3.14). In practice, the cells must be expressed as a particular type of mapping over a set of admissible geometries (polytopes, see Sect. 3.14). Thus, for every element $K \in \mathcal {T}_h$, we assume that there is a reference cell ${\hat{K}}_K$ and a diffeomorphism $\varvec{\Phi }_K: {\hat{K}}\rightarrow K$. In what follows, we usually use the notation ${\hat{\varvec{x}}} \, \doteq \, \varvec{\Phi }_K^{-1}(\varvec{x})$.

The definition of the functional space also relies on a reference functional space as follows: (1) we define a functional space in the reference cell ${\hat{K}}$; (2) we define a set of functions in the physical cell $K$ via a function mapping; (3) we define the global space as the assemble of cell-based spaces plus continuity constraints between cells. In order to present this process, we introduce the concept of reference FE, FE, and FE space, respectively.

### The FE Concept in the Reference and Physical Spaces

Using the abstract definition of Ciarlet, a FE is represented by the triplet $\{ K, \mathcal {V}, \Sigma \}$, where $K$ is a compact, connected, Lipschitz subset of $\mathbb {R}^d$, $\mathcal {V}$ is a vector space of functions, and $\Sigma$ is a set of linear functionals that form a basis for the dual space $\mathcal {V}'$. The elements of $\Sigma$ are the so-called DOFs of the FE. We denote the number of moments as $n_\Sigma$. The moments can be written as $\sigma _a$ for $a \in {\mathcal {N}}_\Sigma \, \doteq \, \{ 1, \ldots ,n_\Sigma \}$. We can also define the basis $\{\phi ^{a}\}_{a \in {\mathcal {N}}_\Sigma }$ for $\mathcal {V}$ such that $\sigma _a(\phi ^{b}) = \delta _{a b}$ for $a,\, b \in {\mathcal {N}}_\Sigma$. These functions are the so-called *shape functions* of the FE, and there is a one-to-one mapping between shape functions and DOFs. Given a function *v*, we define the *local interpolator* for the FE at hand as

6 $$\begin{aligned} \pi _K(v) \, \doteq \, \sum _{a \in {\mathcal {N}}_\Sigma } \sigma _a (v) \phi ^{a}. \end{aligned}$$

It is easy to check that the interpolation operator is in fact a projection.

In the reference space, we build *reference* FEs $({\hat{K}},\hat{\mathcal {V}},{\hat{\Sigma }})$ as follows. First, we consider a bounded set of possible cell geometries, denoted by ${\hat{K}}$; see the definition of polytopes in Sect. 3.14. On ${\hat{K}}$, we build a functional space $\hat{\mathcal {V}}$ and a set of DOFs ${\hat{\Sigma }}$. We consider some examples of reference FEs in Sects. 3.8, 3.9, and 3.10.

In the physical space, the FE triplet $(K,\mathcal {V},\Sigma )$ on a mesh cell $K\in \mathcal {T}_h$ relies on: (1) a reference FE $({\hat{K}},\hat{\mathcal {V}},{\hat{\Sigma }})$, (2) a geometrical mapping $\varvec{\Phi }_K$ such that $K\, \doteq \, \varvec{\Phi }_K({\hat{K}})$, and (3) a linear bijective function mapping ${\hat{\Psi }}_K: \hat{\mathcal {V}}\rightarrow \hat{\mathcal {V}}$. The functional space in the physical space is defined as $\mathcal {V}\, \doteq \, \{ \hat{\Psi }_K({\hat{v}}) \circ \varvec{\Phi }^{-1}_K: \, {\hat{v}} \in \hat{\mathcal {V}}\}$; we will also use ${\Psi }_K: \hat{\mathcal {V}}\rightarrow \mathcal {V}$ defined as ${\Psi }_K({\hat{v}}) \, \doteq \, {\hat{\Psi }}_K({\hat{v}}) \circ \varvec{\Phi }^{-1}_K$. The set of DOFs in the physical space is defined as $\Sigma \, \doteq \, \{ {\hat{\sigma }} \circ {\Psi }_K^{-1} \, : \, {\hat{\sigma }} \in {\hat{\Sigma }}\}$. Given the set of shape functions $\{ \hat{\phi }^{a} : a \in {\mathcal {N}}_{\hat{\Sigma }}\}$ in the reference FE, it is easy to check that $\{ \phi _K^{a} \, \doteq \, \Psi _K( \hat{\phi }^{a} ) : a \in {\mathcal {N}}_{\hat{\Sigma }}\}$ are the set of shape functions of the FE in the physical space.

The reference FE space $\hat{\mathcal {V}}$ is usually a polynomial space. Thus, the first ingredient is to define bases of polynomials; see Sect. 3.4. The analytical expression of the basis of shape functions is not straightforward for complicated definitions of moments; this topic is covered in Sect. 3.5. After that, we will consider how to build global (and conforming) FE spaces in Sect. 3.6, and how to integrate the bilinear forms in the corresponding weak formulation in Sect. 3.7. We finally provide three examples of FEs in Sects. 3.8, 3.9, and 3.10.

### Construction of Polynomial Spaces

Local FE spaces are usually polynomial spaces. Given an order $k \in \mathbb {N}$ and a set ${\mathcal {N}}_k$ of distinct points (nodes) in $\mathbb {R}$ (we will indistinctly represent nodes by their index *i* or position $x_i$), we define the corresponding set of Lagrangian polynomials $\{ \ell ^k_0, \ldots , \ell ^k_k \}$ as:

7 $$\begin{aligned} \ell ^k_m(x) \, \doteq \, \frac{ \Pi _{n \in {\mathcal {N}}_k \setminus \{m\} } (x - x_s) }{ \Pi _{n \in {\mathcal {N}}_k \setminus \{m\} } (x_m - x_s) }. \end{aligned}$$

We can also define the Lagrangian basis $\mathcal {L}^k = \{ \ell ^k_i \, : \, 0 \le i \le k \}$. This set of polynomials are a basis for *k*-th order polynomials. We note that $\ell ^k_m(x_l) = \delta _{ml}$, for $0 \le m, \, l \le k$.

For multi-dimensional spaces, we can define the set of nodes as the Cartesian product of 1D nodes. Given a *d*-tuple order ${\varvec{k}}$, we define the corresponding set of nodes for n-cubes as: ${\mathcal {N}}^{\varvec{k}}\, \doteq \, {\mathcal {N}}^{k_1} \times \cdots \times {\mathcal {N}}^{k_d}$. Analogously, we define the multi-dimensional Lagrange basis

8 $$\begin{aligned} \mathcal {L}^{\varvec{k}}= \{ \ell ^{\varvec{k}}_{\varvec{m}} \, : {\varvec{m}} \in {\mathcal {N}}^{\varvec{k}}\}, \qquad \hbox { where } \quad \ell ^{\varvec{k}}_{\varvec{m}}(\varvec{x}) \, \doteq \, \Pi _{i=1}^d \ell ^{k_i}_{m_i}(x_i). \end{aligned}$$

Clearly, $$\ell _{\varvec{t}}^{\varvec{k}}(\varvec{x}_{\varvec{s}}) = \delta _{{\varvec{s}}\varvec{t}}$$, for $${\varvec{s}}, \, \varvec{t} \in {\mathcal {N}}^{\varvec{k}}$$.

This Cartesian product construction leads to a basis for the local FE spaces usually used on n-cubes, i.e., the space of polynomials that are of degree less or equal to *k* with respect to each variable $x_1, \ldots , x_d$. We can define monomials by a *d*-tuple $\varvec{\alpha }$ as $p_{\varvec{\alpha }}(\varvec{x}) \, \doteq \, \Pi _{i=1}^d x_i^{\alpha _i}$, and the polynomial space of order ${\varvec{k}}$ as ${\mathcal {Q}}_{\varvec{k}}= \mathrm{span} \{ p_{\varvec{\alpha }}(\varvec{x}) \, : 0 \le \alpha _i \le k_i, \, i = 1, \ldots , d \}$. We have ${\mathcal {Q}}_{\varvec{k}}= \mathrm{span} \{ \ell \, : \, \ell \in \mathcal {L}^{\varvec{k}}\}$.

The definition of polynomial spaces on n-simplices is slightly different. It requires the definition of the space of polynomials of degree equal or less than *k* in the variables $x_1,\ldots ,x_d$. It does not involve a full Cartesian product of 1D Lagrange polynomials (or monomials) but a truncated space, i.e., the corresponding polynomial space of order *k* is ${\mathcal {P}}_k = \mathrm{span} \{ p_{\varvec{\alpha }}(\varvec{x}) \, : | \varvec{\alpha }| \le k \}$, with $| \varvec{\alpha }| \, \doteq \, \sum _{i=1}^d \alpha _i$. Analogously as for n-cubes, a basis for the dual space of ${\mathcal {P}}_k$ are the values at the set of nodes $\tilde{{\mathcal {N}}}^{k} \, \doteq \, \{ {\varvec{s}}\in {\mathcal {N}}^{k\varvec{1}} \, : \, |{\varvec{s}}| \le k \}$. It generates the typical grad-conforming FEs on n-simplices.

### Construction of the Shape Functions Basis

The analytical expression of shape functions can become very complicated for high order FEs and non-trivial definitions of DOFs, e.g., for electromagnetic applications. Furthermore, to have a code that provides a basis for an arbitrary high order, an automatic generator of shape functions must be implemented. When the explicit construction of the shape functions is not obvious, we proceed as follows.

Let us consider a FE defined by $\{ K, \mathcal {V}, \Sigma \}$.^4^ First, we generate a *pre-basis* $\{ {\psi }^b \}_{b \in \Sigma }$ that spans the local FE space $\mathcal {V}$, e.g., a Lagrangian polynomial basis (see Sect. 3.4). On the other hand, given the set of local DOFs, we proceed as follows. The shape functions can be written as ${\phi }^a = \sum _{b\in {\mathcal {N}}_\Sigma } \varvec{\Phi }_{ab} {\psi }^b$, where ${\psi }^b$ are the elements of the pre-basis. By definition, the shape functions must satisfy $\sigma _{a}({\phi }^b) = \delta _{ab}$ for $a, \, b \in {\mathcal {N}}_\Sigma$. As a result, let us define $\mathbf {C}_{ab} \, \doteq \, \sigma _{a}({\psi }^b)$. We have (using Einstein’s notation):

$$\begin{aligned} \sigma _{a}({\phi }^b) = \sigma _{a}( \varvec{\Phi }_{bc} {\psi }^c ) = \sigma _{a} ({\psi }^c) \varvec{\Phi }_{bc} = \delta _{ab}, \end{aligned}$$

or in compact form, $\mathbf {C} \varvec{\Phi }^T = I$, and thus $\varvec{\Phi }^T = \mathbf {C}^{-1}$. As a result, $\varvec{\Phi }_{ab} = \mathbf {C}^{-1}_{ba}$. The shape functions are computed as a linear combination of the pre-basis functions.

### Global FE Space and Conformity

Finally, we must define the *global* FE space. Conforming FE spaces are defined as: $\mathcal {X}_h\, \doteq \, \{ v \in \mathcal {X}\, : \, v|_K \in \mathcal {V}\}.$ The main complication in this definition is to enforce the conformity of the FE space, i.e., $\mathcal {X}_h\subset \mathcal {X}$. In fact, the conformity constraint is the one that motivates the choice of ${\hat{\Sigma }}$ and $\Psi$, and as a consequence, $\Sigma$. In practice, the conformity constraint must be re-stated as a continuity constraint over FE DOFs. In general, these constraints are implicitly enforced via a global DOF numbering, even though it is not possible in general for adaptive schemes with non-conforming meshes and/or variable order cells, which require more involved constraints.

Let us define by $\mathcal {M}_h \, \doteq \, \{ (b,K) : b \in {\mathcal {N}}_{\Sigma _K}, \, K\in \mathcal {T}_h\}$ the Cartesian product of local DOFs for all cells. We define the global DOFs as the quotient space of $\mathcal {M}_h$ by an equivalence relation $\sim$. Using standard notation, given $\sim$, the equivalence class of $a \in \mathcal {M}_h$ with respect to $\sim$ is represented with $[a] \, \doteq \, \{ b \in \mathcal {M}_h \, : \, a \sim b \}$, and the corresponding quotient set is ${\mathcal {N}}_h \, \doteq \, \{ [a] \, : \, a \in \mathcal {M}_h \}$. The set ${\mathcal {N}}_h$ is the set of global DOF and $[\cdot ]$ represents the local-to-global DOF map. We assume that the equivalence relation is such that if two elements $(b,K), \, (b',K') \in \mathcal {M}_h$ are such that $(b,K) \sim (b',K')$, then $K \ne K'$.^5^ Using the one-to-one mapping between moments and shape functions, the same operator allows one to define global shape functions $\phi ^{a} = \sum _{(b,K) \sim a} \phi ^{b}_K$. We assume that the choices above are such that they satisfy the conformity constraint, i.e., $\mathcal {X}_h= \mathrm{span}\{ \phi ^{a} \}_{a \in {\mathcal {N}}_h} \subset \mathcal {X}$.

Let us consider an infinite-dimensional space $\tilde{\mathcal {X}}$ such that (1) $\mathcal {X}_h\subset \tilde{\mathcal {X}} \subset \mathcal {X}$ and (2) for every function $v \in \tilde{\mathcal {X}}$ and global DOF $a \in {\mathcal {N}}_h$, all the local DOFs $b, \, b' \in [a]$ are such that $\sigma _b(v) = \sigma _{b'}(v)$, i.e., local DOF related to the same global DOF are continuous among cells. The *global interpolator* is defined as:

9 $$\begin{aligned} \pi _{\mathcal {X}_h}(v) \, \doteq \, \sum _{K\in \mathcal {T}_h} \pi _K(v) = \sum _{K\in \mathcal {T}_h} \sum _{b \in {\mathcal {N}}_{\Sigma _K} } \sigma _b(v) \phi ^{b}_K, \qquad \hbox {for } v \in \tilde{\mathcal {X}}. \end{aligned}$$

It is easy to check that it is in fact a projector. In any case, we use *projection operator* to refer to other projectors that involve the solution of a global FE system, e.g., based on the minimization of the $L^2$ or $H^1$ norm.

Below, we provide details about how to choose the local DOFs ${\hat{\Sigma }}$, the function map $\Psi$, and the equivalence relation $\sim$ such that the conformity property is satisfied for grad, div, and curl-conforming FE spaces. The case of non-conforming methods, e.g., DG methods, can readily be considered. In this case, the conformity constraint is not required, which leads to much more flexibility in the definition of DOFs. On the other side, these schemes require numerical perturbations of the continuous bilinear and linear forms in (4) that involve integrals over the facets of FEs to *weakly* enforce the conformity. (Facets are $(d-1)$-faces, e.g., faces in 3D and edges in 2D).

Once we have defined a basis for the FE spaces $\mathcal {X}_h$ and $\mathcal {Y}_h$ using the FE machinery presented above, every FE function $u_h$ can be uniquely represented by a vector $\mathbf {u}\in \mathbb {R}^{|{\mathcal {N}}_h|}$ as $u_h = \sum _{b \in {\mathcal {N}}_h} \phi ^{b} \mathbf {u}_b$. In fact, problem (4) can be re-stated as: find $\mathbf {u}\in \mathbb {R}^{|{\mathcal {N}}_h|}$ such that

$$\begin{aligned} {a_h}(\phi ^{b},\psi ^{a}) \mathbf {u}_b = \ell _h( \psi ^{a}) , \qquad \hbox {for any } \, a \in {\mathcal {N}}_h. \end{aligned}$$

We have ended up with a finite-dimensional linear problem, i.e., a linear system. We note that in general, the trial space moments can be different from the ones of the test space, as soon as the cardinality is the same. In matrix form, the problem can be stated as:

10 $$\begin{aligned} \mathrm {Solve} \ \ \ \mathbf {A}\mathbf {u}= \mathbf {f}, \qquad \hbox {with } \quad \mathbf {A}_{ab} \, \doteq \, {a_h}(\phi ^{b},\psi ^{a}), \quad \mathbf {f}_{a} \, \doteq \, \ell _h( \psi ^{a}) . \end{aligned}$$

Assuming that the bilinear form can be split into cell contributions as ${a_h}(\cdot ,\cdot ) = \sum _{K\in \mathcal {T}_h} {a}_{K}(\cdot ,\cdot )$, e.g., by replacing $\int _\Omega$ by $\sum _{K\in \mathcal {T}_h} \int _K$, the construction of the matrix is implemented through a cell-wise assembly process, as follows:

11 $$\begin{aligned} \mathbf {A}_{[a][b]} = \sum _{K\in \mathcal {T}_h} \sum _{a,b \in {\mathcal {N}}_{\Sigma _K} }\mathbf {A}_{ab}^{K} \, \doteq \, \sum _{K\in \mathcal {T}_h} \sum _{a,b \in {\mathcal {N}}_{\Sigma _K} } {a}_{K}(\phi ^{b}_K,\psi ^{a}_K). \end{aligned}$$

The FE affine operator (5) can be represented as $\mathcal {F}_h(u_h) \, \doteq \, \mathbf {A}\mathbf {u}- \mathbf {f}$, i.e., it can be represented with a matrix and a vector of size ${|{\mathcal {N}}_h|}$.

### Numerical Integration

In general, the local bilinear form can be stated as:

$$\begin{aligned} {a}_{K}(\phi ^{b}_K,\psi ^{a}_K) = \int _K\varvec{\mathcal {F}}(\varvec{x}) \mathrm{d}\Omega , \end{aligned}$$

where the evaluation of $\varvec{\mathcal {F}}(\varvec{x})$ involves the evaluation of shape function derivatives. Let us represent the Jacobian of the geometrical mapping with $\varvec{J}_K\, \doteq \, \frac{\partial \varvec{\Phi }_K}{\partial \varvec{x}}$. We can rewrite the cell integration in the reference cell, and next consider a quadrature rule $\mathrm{Q}$ defined by a set of points/weights $(\hat{\varvec{x}}_\mathrm{gp}, \mathrm{w}_\mathrm{gp})$, as follows:

12 $$\begin{aligned} \int _K\varvec{\mathcal {F}}(\varvec{x}) \mathrm{d} \Omega = \int _{\hat{K}}\varvec{\mathcal {F}}\circ \varvec{\Phi }(\varvec{x}) |\varvec{J}_K| \mathrm{d}\Omega = \sum _{{\hat{\varvec{x}}_\mathrm{gp} \in \mathrm {Q}}} \varvec{\mathcal {F}}\circ \varvec{\Phi }(\hat{\varvec{x}}_\mathrm{gp}) \mathrm{w}(\hat{\varvec{x}}_\mathrm{gp}) |\varvec{J}_K(\hat{\varvec{x}}_\mathrm{gp})|. \end{aligned}$$

Here, the main complication is the evaluation of $\varvec{\mathcal {F}}\circ \varvec{\Phi }(\hat{\varvec{x}}_\mathrm{gp})$. By construction, the evaluation of this functional only requires the evaluation of $\partial _{\varvec{\alpha }}\phi ^{b}_K \circ \varvec{\Phi }(\hat{\varvec{x}}_\mathrm{gp})$ for some values of the multi-index $\varvec{\alpha }$ (idem for the test functions). Usually, $|\varvec{\alpha }| \le 2$ in $\mathcal {C}^0$ FEs, since higher-order derivatives would require higher inter-cell continuity. The second derivatives, which only have sense for *broken* cell-wise integrals, are in fact only needed for some method based on *stabilization* techniques based on the pointwise evaluation of residuals in the interior of cells [46].

Let us consider the case of zero and first derivatives, i.e., the evaluation of $\phi ^{b}_K \circ \varvec{\Phi }_K(\hat{\varvec{x}}_\mathrm{gp})$ and ${\varvec{\nabla }}\phi ^{b}_K \circ \varvec{\Phi }_K(\hat{\varvec{x}}_\mathrm{gp})$. The values of the shape functions (times the geometrical mapping) on the quadrature points is determined as follows:

13 $$\begin{aligned} \phi ^{b}_K\circ \varvec{\Phi }_K(\hat{\varvec{x}}_\mathrm{gp})= {\hat{\Psi }}(\hat{\phi }^{b})(\hat{\varvec{x}}_\mathrm{gp}), \end{aligned}$$

whereas shape function gradients are computed as:

14 $$\begin{aligned} {\varvec{\nabla }}\phi ^{b}_K\circ \varvec{\Phi }_K(\hat{\varvec{x}}_\mathrm{gp}) = {\varvec{\nabla }}( {\hat{\Psi }}(\hat{\phi }^{b}) \circ \varvec{\Phi }_K^{-1}) \circ \varvec{\Phi }_K(\hat{\varvec{x}}_\mathrm{gp}) = {\varvec{\nabla }}_{\hat{\varvec{x}}} {\hat{\Psi }}(\hat{\phi }^{b})(\hat{\varvec{x}}_\mathrm{gp}) \varvec{J}_K^{-1}(\hat{\varvec{x}}_\mathrm{gp}), \end{aligned}$$

where we have used some elementary differentiation rules and the inverse function theorem in the last equality; ${\varvec{\nabla }}_{\hat{\varvec{x}}}$ represents the gradient in the reference space. Thus, one only needs to provide the values of the Jacobian, its inverse, and its determinant, from one side, and the value of the shape functions $\Psi (\hat{\phi }^{b})$ and their gradients ${\varvec{\nabla }}_{\hat{\varvec{x}}} \Psi (\hat{\phi }^{b})$ in the reference space, on the other side, at all quadrature points, to compute all the entries of the FE matrices; second order derivatives can be treated analogously.

Quadrature rules for ${\hat{K}}$ being an n-cube can readily be obtained as a tensor product of a 1D quadrature rule, e.g., the Gauss-Legendre quadrature. Symmetric quadrature rules on triangles and tetrahedra for different orders can be found, e.g., in [69]. In any case, to create arbitrarily large quadrature rules for n-simplices, one can consider the so-called Duffy transformation [73, 74].

As it is well known, considering n-cube topologies for ${\hat{K}}$, Gauss quadratures with *n* points per direction can integrate *exactly* $2n-1$ order polynomials. e.g., For a Lagrangian reference FE of order *p* and an affine geometrical map, we choose $n=p+ \mathrm{ceiling}( 1/2 ) = p+1$ per direction to integrate exactly a mass matrix. For n-simplex meshes, we use either symmetric quadratures (if available) or tensor product rules plus the Duffy transformation [73, 74]. The latter case is based on introducing a change of variables that transform our n-simplex integration domain into an n-cube, and integrate on the n-cube using tensor product quadratures. It is worth noting that this change of variables introduces a non-constant Jacobian. The determinant of the Jacobian is of order at most $d-1$ with respect to each variable. To integrate a mass matrix exactly, we must be able to integrate exactly polynomials of order $2p+d-1$. Therefore, we need to take $n=p+ \mathrm{ceiling}( d/2 )$ to exactly integrate mass matrices.

### Grad-Conforming FEs: Lagrangian (Nodal) Elements

In this section, we consider one characterization of the abstract FE technology above. First, we are interested in the so-called nodal FEs, based on Lagrange polynomials and DOFs based on nodal values.

Let us consider the same order for all components, i.e., $k \varvec{1}\, \doteq \, (k ,\ldots , k)$. When the reference geometry ${\hat{K}}$ is an n-cube, we define the reference FE space as $\mathcal {V}_k \, \doteq \, {\mathcal {Q}}_{k \varvec{1}}$. The set of nodes ${\mathcal {N}}^{k\varvec{1}}$ can be generated, e.g., from the equidistant Lagrangian nodes. Let us define the bijective mapping ${\mathtt {i}}(\cdot )$ from the set of nodes ${\mathcal {N}}^{k\varvec{1}}$ to $\{1, \ldots , |{\mathcal {N}}^{k\varvec{1}}| \} \equiv {\mathcal {N}}_\Sigma$, i.e., the local node numbering. The set of local DOFs ${\mathcal {N}}_{\Sigma _K}$ are the nodal values, i.e., $\sigma _{{\mathtt {i}}(\varvec{s})} \, \doteq \, v(\varvec{x}_{\varvec{s}})$, for $\varvec{s} \in {\mathcal {N}}^{\varvec{k}}$. Clearly, the reference FE shape functions related to these DOFs are $\phi ^{{\mathtt {i}}(\varvec{s})} \, \doteq \, \ell _{\varvec{s}}^{k\varvec{1}}$. On the other hand, we simply take ${\hat{\Psi }}(v) \, \doteq \, v$.

For n-simplices, we consider the reference FE space ${\mathcal {P}}_k$ spanned by the pre-basis $\{ p_{\varvec{\alpha }}(\varvec{x}) \, : 0 \le \alpha _i \le k, \, i = 1, \ldots , d \}$ and the set of nodes $\tilde{{\mathcal {N}}}^{k}$ (see Sect. 3.4). The set of local DOFs ${\mathcal {N}}_{\Sigma _K}$ are the nodal values. Since the pre-basis elements are not shape functions, we proceed as in Sect. 3.5 to generate the expression of the shape functions basis for arbitrary order reference FEs on n-simplices.

The global FE space is determined by the following equivalence relation. The set of local DOFs for n-cubes is $\mathcal {M}_h \, \doteq \, \{ (\varvec{s},K) \, : \, \varvec{s}\in {\mathcal {N}}^{k \varvec{1}}, K \in \mathcal {T}_h\}$ due to the one-to-one mapping between DOFs and nodes; we replace the set of nodes by $\tilde{{\mathcal {N}}}^{k}$ for n-simplices. Furthermore, we say that $(\varvec{s},K) \sim (\varvec{s}',K')$ iff $\varvec{x}_{\varvec{s}} = \varvec{x}_{\varvec{s}'}$. The implementation of this equivalence relation, and thus, the global numbering, relies on the ownership relation between n-faces and DOFs (e.g., in 3D we can say whether a DOF belongs to a vertex, edge, or face) and a permutation between the local node numbering in $K^+$ to the one in $K ^-$ for nodes on *F*. See Sect. 3.14 for more details. With such global DOF definition, it is easy to check that the global FE space functions are $\mathcal {C}^0$ and thus grad-conforming.

Since Lagrangian moments involve point-wise evaluations of functions and $H^1_0(\Omega ) \not \subset \mathcal {C}^0(\Omega )$ for $d>1$, the interpolator (9) is not defined in such space. Instead, we consider that functions to be interpolated belong, e.g., to the space $\tilde{\mathcal {X}} \, \doteq \, \mathcal {C}^0(\Omega )$.

When one has to deal with vector or tensor fields, we can generate them as a Cartesian product of scalar spaces as follows. We define the local FE space $\varvec{\mathcal {V}}_k \, \doteq \, [{\mathcal {Q}}_{k \varvec{1}}]^d$ and the function map ${\hat{\Psi }}(\varvec{v}) \, \doteq \, \varvec{v}$. In the vector case, the local DOFs set is represented with $\mathcal {M}_h \, \doteq \, \{ (i,\varvec{s},K) \, : \, 1 \le i \le d, \, \varvec{s}\in {\mathcal {N}}^{k \varvec{1}}, K \in \mathcal {T}_h\}$, and $(i,\varvec{s},K) \sim (i',\varvec{s}',K')$ iff $i = i'$ and $\varvec{x}_{\varvec{s}} = \varvec{x}_{\varvec{s}'}$. Analogously, shape functions are computed as ${\phi }^a \, \doteq \, \sum _{(i,\varvec{s},K) \sim a} \ell _{\varvec{s}}^{k \varvec{1}} \mathbf {\varvec{e}}_i$; $\mathbf {\varvec{e}}_i$ represents the *i*-th canonical basis vector of $\mathbb {R}^d$. We proceed analogously for n-simplices.

The verification that two nodes are in the same position is not straightforward. First, for every node $\varvec{s}$ in *K*, we can assign an n-face owner *F* (e.g., a vertex, edge, face, or cell); cell DOFs are not replicated. Given a node $\varvec{s}\in {\mathcal {N}}^{k \varvec{1}}$ of cell *K* that belongs to the n-face *F*, it can be determined by an index $\varvec{s}_F$ with respect to *F* and *K*. Analogously, another node that belongs to the same n-face but cell $K'$, is represented by $\varvec{s}_F'$. On the other hand, one can define a permutation mapping

15 $$\begin{aligned} \mathtt {p}_F(F,K,K';\cdot ), \end{aligned}$$

that, given the local index of a node within the n-face *F* with respect to *K*, it provides the index in the n-face *F* with respect to $K'$ (see Sects. 3.13 and 3.16 for more details). Thus, $\varvec{x}_{\varvec{s}} = \varvec{x}_{\varvec{s}'}$ iff $\mathtt {p}_F(F,K,K';\varvec{s}_F) = \varvec{s}_F'$.

### Div-Conforming FEs

We present the so-called Raviart-Thomas FEs for vector fields [5]; the implementation of Brezzi-Douglas-Marini FEs is analogous. In this case, the order being used is different at every space dimension. Let us start with Raviart-Thomas FEs on n-cubes. In 2D, the space reads as ${\varvec{\mathcal {V}}}_{k} \, \doteq \, {\mathcal {Q}}_{(k+1,k)} \times {\mathcal {Q}}_{(k,k+1)}$, whereas in 3D it reads as ${\varvec{\mathcal {V}}}_{k} \, \doteq \, {\mathcal {Q}}_{(k+1,k,k)} \times {\mathcal {Q}}_{(k,k+1,k)} \times {\mathcal {Q}}_{(k,k,k+1)}$; the Raviart-Thomas element can in fact be considered for any dimension. The basis for $\Sigma$ in 3D is composed of two types of DOFs, boundary and interior DOFs, defined as

16 $$\begin{aligned}&\frac{1}{\Vert {\hat{F_0}} \Vert } \int _{{\hat{F}}_0} \varvec{v}\cdot \varvec{n}\circ \varvec{\Phi }_{\hat{F}}\, q \mathrm{d}\Gamma , \quad q \in {\mathcal {P}}_{k}, \quad \frac{1}{\Vert {\hat{K}} \Vert }\int _{\hat{K}} \varvec{v}\cdot \ \varvec{q}\mathrm{d}\Omega , \quad \varvec{q}\in {\mathcal {Q}}_{(k-1,k,k)} \nonumber \\&\quad \times {\mathcal {Q}}_{(k,k-1,k)} \quad \times {\mathcal {Q}}_{(k,k,k-1)}, \end{aligned}$$

respectively^6^; the 2D case is straightforward, replacing the space of shape functions for the interior moments by ${\mathcal {Q}}_{(k-1,k)} \times {\mathcal {Q}}_{(k,k-1)}$. The definition of the boundary facets involves mappings from a reference facet ${\hat{F}}_0$ to all facets ${\hat{F}}$ of the FE *K*, i.e., $\varvec{\Phi }_{\hat{F}}: {\hat{F}}_0\rightarrow {\hat{F}}$. Every boundary moment can be associated to a function in a Lagrangian space, and thus, a node index. As a result, the boundary DOFs can be indexed with a node in ${\mathcal {N}}^{k\varvec{1}}$ (for $d=2$) on the corresponding facet *F*, i.e., $\mathcal {M}_h^\partial \, \doteq \, \{ (F,\varvec{s},K) \, : \, F \hbox { are facets of } K, \, \varvec{s}\in {\mathcal {N}}^{k \varvec{1}}, K \in \mathcal {T}_h\}$. We say that $(F,\varvec{s},K) \sim (F',\varvec{s}',K')$ iff $F = F'$ and $\varvec{x}_{\varvec{s}} = \varvec{x}_{\varvec{s}'}$. To check whether $\varvec{x}_{\varvec{s}} = \varvec{x}_{\varvec{s}'}$ holds, we can proceed similarly as for Lagrangian elements. The shape functions are built as in Sect. 3.5. We consider a Lagrangian pre-basis for $\mathcal {V}$, and compute the shape functions via a change-of-basis. The function mapping reads as follows:

17 $$\begin{aligned} {\hat{\Psi }}_K(\varvec{v}) \, \doteq \, \frac{1}{|\varvec{J}_K|} \varvec{J}_K\varvec{v}; \end{aligned}$$

the mapping ${\hat{\Psi }}_K \circ \varvec{\Phi }^{-1}_K$ is the so-called contravariant Piola transformation. One can check that the definition of this mapping together with the assembly defined above leads to a global FE space that is div-conforming; i.e., its functions have continuous normal component across inter-cell facets. Thus, $\mathcal {X}_h\subset H(\mathrm{div}, \Omega )$ [5].

On n-simplices, the reference FE space is ${\varvec{\mathcal {V}}}_{k} \, \doteq \, [{\mathcal {P}}_{k}]^d \times \varvec{x}{\mathcal {P}}_{k}$, for $k = 0, 1, 2, \ldots$, and the basis for $\Sigma$ is composed of the following boundary and interior DOFs:

$$\begin{aligned} \frac{1}{\Vert {\hat{F_0}} \Vert } \int _{{\hat{F}}_0} \varvec{v}\cdot \varvec{n}\circ \varvec{\Phi }_{\hat{F}}\, q \mathrm{d}\Gamma , \quad q \in {\mathcal {P}}_{k}, \quad \frac{1}{\Vert {\hat{K}} \Vert }\int _{\hat{K}} \varvec{v}\cdot \ \varvec{q}\mathrm{d}\Omega , \quad \varvec{q}\in [{\mathcal {P}}_{k-1}]^d. \end{aligned}$$

In this case, the generation of the pre-basis is not a Lagrangian FE space of functions, but it can easily be expressed as the span of vector functions with components in a selected subset of ${\mathcal {P}}_{k+1}$.

### Curl-Conforming FEs

The weak formulation of electromagnetic problems involve the functional space $H(\mathbf{curl},\Omega )$. Conforming FE spaces for $H(\mathbf{curl},\Omega )$ must preserve the continuity of the tangential component of the field. The so-called edge elements (or Nédélec elements) are curl-conforming FEs [72]. As Raviart-Thomas elements, the edge elements pre-basis on n-cubes involves different orders per dimension and per component. In 2D, the space reads as ${\varvec{\mathcal {V}}}_{k} \, \doteq \, {\mathcal {Q}}_{(k-1,k)} \times {\mathcal {Q}}_{(k,k-1)}$, whereas in 3D it reads as ${\varvec{\mathcal {V}}}_{k} \, \doteq \, {\mathcal {Q}}_{(k-1,k,k)} \times {\mathcal {Q}}_{(k,k-1,k)} \times {\mathcal {Q}}_{(k,k,k-1)}$. The basis for $\Sigma$ is composed of three types of DOFs (in 3D), namely edge, face, and interior DOFs, defined as:

$$\begin{aligned}&\frac{1}{\Vert {\hat{E_0}} \Vert } \int _{{\hat{E}_0}} ( \varvec{v}\cdot \varvec{\tau } ) \circ \varvec{\Phi }_{\hat{E}} q \, \mathrm{d} \Lambda , \quad \forall q \in \mathcal {P}_{k-1}, \\&\frac{1}{\Vert {\hat{F}_0} \Vert } \int _{{\hat{F}_0}} ( \varvec{J}_{\hat{F}}^T (\varvec{v}\times \varvec{n}) ) \circ \varvec{\Phi }_{\hat{F}}\cdot \varvec{q} \,\mathrm{d}\Gamma , \quad \forall \varvec{q} \in \mathcal {Q}_{(k-2,k-1)}\times \mathcal {Q}_{(k-1,k-2)}, \\&\frac{1}{\Vert \hat{K} \Vert } \int _{\hat{K}} \varvec{v}\cdot \varvec{q} \, \mathrm{d} \Omega , \quad \forall \varvec{q} \in \mathcal {Q}_{(k-1,k-2,k-2)}\times \mathcal {Q}_{(k-2,k-1,k-2)} \times \mathcal {Q}_{(k-2,k-2,k-1)}, \end{aligned}$$

respectively, where the edge map $\varvec{\Phi }_{\hat{E}}$ is defined as the one for the face. The boundary DOFs can be indexed by a triplet $(F,\varvec{s},K)$, where *F* can be an edge or a face in 3D, following the same ideas as for Raviart-Thomas elements. In this case, the function mapping reads as follows:

18 $$\begin{aligned} \hat{\Psi }_K(\varvec{v}) \, \doteq \, \varvec{J}_K^{-T} \varvec{v}; \end{aligned}$$

the mapping ${\hat{\Psi }}_K \circ \varvec{\Phi }^{-1}_K$ is the so-called covariant Piola transformation, which leads to a global FE space that is curl-conforming [72], i.e., its functions have continuous tangential component across inter-cell facets.

On n-simplices, the space reads as:

19 $$\begin{aligned} {\varvec{\mathcal {V}}}_{k} \, \doteq \, [ {\mathcal {P}}_{k} ]^d + \varvec{\mathcal {S}}_{k}, \, \hbox { where } \, \varvec{\mathcal {S}}_{k} \, \doteq \, \{ \varvec{v} \in [{\mathcal {P}}_{k+1}]^d \, : \, \varvec{v} (\varvec{x}) \cdot \varvec{x}= 0 \, \forall \, \varvec{x}\in {\hat{K}}\}. \end{aligned}$$

The basis for $\Sigma$ in 3D is composed of the following boundary and interior DOFs:^7^

$$\begin{aligned}&\frac{1}{\Vert {\hat{E_0}} \Vert } \int _{{\hat{E}_0}} ( \varvec{v}\cdot \varvec{\tau } ) \circ \varvec{\Phi }_{\hat{E}} q \, \mathrm{d} \Lambda , \quad \forall q \in \mathcal {P}_{k-1}, \\&\frac{1}{\Vert {\hat{F}_0} \Vert } \int _{{\hat{F}_0}} ( { \varvec{J}}^T_{\hat{F}} (\varvec{v}\times \varvec{n}) ) \circ \varvec{\Phi }_{\hat{F}}\cdot \varvec{q} \,\mathrm{d}\Gamma , \quad \forall \varvec{q} \in [ \mathcal {P}_{k-2} ]^2 \\&\frac{1}{\Vert \hat{K} \Vert } \int _{\hat{K}} \varvec{v}\cdot \varvec{q} \, \mathrm{d} \Omega , \quad \forall \varvec{q} \in [ \mathcal {P}_{k-3} ]^3. \end{aligned}$$

In 2D, only the first two types of DOFs are required, where the first one is now related to facets (edges in 2D) and the second one are interior DOFs owned by the cell. As for Raviart-Thomas elements, the pre-basis functions are not Lagrangian shape functions, but they can again be expressed as the span of vector functions with components in a selected subset of ${\mathcal {P}}_{k+1}$. We refer to [75] for a discussion about the actual generation of a pre-basis for the space (19) in FEMPAR.

### Cartesian Product of FEs for Multi-field Problems

Many problems governed by PDEs involve more than one field, e.g., the Navier-Stokes equations or any multi-physics problem. Let us consider a PDE that involves a set of unknown fields $(\varvec{u}_1 , \ldots , \varvec{u}_n) \in \mathcal {X}^1 \times \ldots \times \mathcal {X}^n$, defined as the Cartesian product of functional spaces. We can proceed as above, and define a FE space for every field space separately, leading to a global FE space $\mathcal {X}^1_h \times \ldots \times \mathcal {X}^n_h$ defined by composition of FE spaces. To define the global numbering of DOFs in the multi-field case, we consider that two DOFs are equivalent if they are related to the same field and satisfy the equivalence relation of the FE space of this field.

The Cartesian product of FE spaces is enough to define volume-coupling multi-physics problems governed on the same physical domain, i.e., the different physics are defined on the whole domain and coupled through volume terms in the formulation. However, many multi-physics problems are interface-based, i.e., the coupling between different physics that are defined on different subdomains is through transmission conditions on the interface. This is the case, e.g., of fluid-structure problems (see, e.g., [76–79]). In these cases, different FE spaces could be defined on different parts of the global mesh, i.e., one must describe the set of subdomains $( \Omega _1, \ldots , \Omega _n )$ of the whole domain $\Omega$ in which the corresponding FE spaces are defined.

### Non-conforming Methods

Up to now, we have considered a global FE space that is conforming, i.e., $\mathcal {X}_h \subset \mathcal {X}$. Alternatively, one can consider FE schemes that are not conforming. Since the original bilinear form has no sense in general for a non-conforming FE space $\mathcal {X}_h$, one shall consider a *stabilized* bilinear form $a_h$ that is well-posed (stable and continuous) in the discrete setting. In general, these schemes replace the required inter-cell continuity for conformity by a weak imposition of such continuity. Thus, the inter-cell continuity is imposed weakly through penalty-like terms. DG methods are schemes of this type [71].

In one sense, non-conforming FE spaces are simpler than conforming ones, since the conformity is not required; one has more flexibility in the definition of local DOFs and the equivalence class concept is not needed, since a DOF never belongs to more than one element. However, the bilinear form usually requires the integration of facet terms, i.e., terms of the type:

$$\begin{aligned} \sum _{F \in \mathcal {F}_h} \int _F \varvec{\mathcal {F}}(\varvec{x}) \mathrm{d}\Omega . \end{aligned}$$

The integration of facet terms is far more complicated than cell terms.

Let us first briefly illustrate a simple application of non-conforming methods, namely the FE discretization of the Poisson problem using the so-called interior penalty (IP) family of DG formulations [71]. Dirichlet boundary conditions constraints, say $u(x)=u_{\mathrm{D}}(x)$ on the whole boundary ${\Gamma }$ of the domain $\Omega$, are to be weakly imposed, as it is natural in such kind of formulations. The global discrete trial space $\mathcal {X}_h$ is composed of functions that are continuous within each cell, but discontinuous across cells, i.e., $\mathcal {X}_h= \{ u_h \in L_2(\Omega ): u_h|_K \in \mathcal {X}_h|_K \subset H^1(K),\ K \in \mathcal {T}_h\}$, and the discrete test space $\mathcal {Y}_h=\mathcal {X}_h$. If we denote $\mathcal {F}^{\Omega }_{h}$ and $\mathcal {F}^{{\Gamma }}_{h}$ as the set of interior and boundary facets of $\mathcal {T}_h$, respectively, the discrete weak form underlying this family of methods reads as: find $u_h \in \mathcal {X}_h$ such that

20 $$\begin{aligned} \begin{aligned}&\sum _{K\in \mathcal {T}_h} \int _K {\varvec{\nabla }}u_h \cdot {\varvec{\nabla }}v_h - \sum _{F\in \mathcal {F}^{\Omega }_{h}} \int _F[\![v_h ]\!] \cdot \{\! \!\{ {\varvec{\nabla }}u_h\}\! \!\} - \tau \sum _{F\in \mathcal {F}^{\Omega }_{h}} \int _F[\![u_h ]\!] \cdot \{\! \!\{ {\varvec{\nabla }}v_h\}\! \!\} \\&\quad + \sum _{F\in \mathcal {F}^{\Omega }_{h}} \gamma |F|^{-1} \int _F[\![u_h ]\!] \cdot [\![v_h ]\!] \\&\quad - \sum _{F\in \mathcal {F}^{{\Gamma }}_{h}} \int _Fv_h {\varvec{\nabla }}{u_h} \cdot \varvec{n}- \tau \sum _{F\in \mathcal {F}^{{\Gamma }}_{h}} \int _Fu_h {\varvec{\nabla }}{v_h} \cdot \varvec{n}+ \sum _{F\in \mathcal {F}^{{\Gamma }}_{h}} \gamma |F|^{-1} \int _Fu_h v_h \\&\quad = \sum _{K\in \mathcal {T}_h} \int _K f v_h - \tau \sum _{F\in \mathcal {F}^{{\Gamma }}_{h}} \int _Fu_{\mathrm{D}} {\varvec{\nabla }}{v_h} \cdot \varvec{n}+ \sum _{F\in \mathcal {F}^{{\Gamma }}_{h}} \gamma |F|^{-1} \int _Fu_{\mathrm{D}} v_h \quad \quad \quad \forall v_h \in \mathcal {Y}_h, \end{aligned} \end{aligned}$$

where $\tau$ is a fixed constant that characterizes the particular method at hand, $\gamma$ is a facet-wise positive constant referred to as penalty parameter, and $|F|$ denotes the surface of the facet; $\tau$ and $\gamma$ should be suitably chosen such that the bilinear form $a_h(u_h,v_h)$ on the left-hand side of (20) is well-posed (stable and continuous) in the discrete setting, and the resulting FE formulation enjoys optimal rates of convergence [71]. Finally, if we denote as $K^+$ and $K^-$ the two cells that share a given facet, then $\{\! \!\{ w_h\}\! \!\}$ and $[\![w_h ]\!]$ denote mean values and jumps of $w_h$ across cells facets:

21 $$\begin{aligned} \{\! \!\{ w_h\}\! \!\} = \frac{w_h^++w_h^-}{2}, \quad \quad [\![w_h ]\!] = w_h^+ \varvec{n}^+ + w_h^- \varvec{n}^-, \end{aligned}$$

with $\varvec{n}^+$, $\varvec{n}^-$ being the facet outward unit normals, and $w_h^+$, $w_h^-$ the restrictions of $w_h$ to the facet, both from either the perspective of $K^+$ and $K^-$, respectively.

The computation and assembly of DOFs related to interior nodes is straightforward. With regard to the facet terms, assuming that we are sitting on an interior facet $F\in \mathcal {F}^{\Omega }_{h}$, four facet-wise matrices, namely $\mathbf {A}^{F}_{K^+ K^+}$, $\mathbf {A}^{F}_{K^+ K^-}$, $\mathbf {A}^{F}_{K^- K^+}$, and $\mathbf {A}^{F}_{K^- K^-}$, are computed. (The case of boundary facets $F\in \mathcal {F}^{{\Gamma }}_{h}$ is just a degenerated case of the one corresponding to interior facets where only a single facet-wise matrix $\mathbf {A}^{F}_{K^+ K^+}$ has to be computed; we omit this sort of facets from the discussion in order to keep the presentation short.) These hold all partial contributions of the facet to the corresponding global entries of the coefficient matrix. The entries of, e.g., $\mathbf {A}^{F}_{K^+ K^-}$, are defined (for our particular problem at hand) as:

22 $$\begin{aligned} \left( \mathbf {A}^{F}_{K^+ K^-}\right) _{ab} = -\int _F[\![\phi ^{b}_{K^-} ]\!] \cdot \{\! \!\{ {\varvec{\nabla }}\phi ^{a}_{K^+}\}\! \!\} - \tau \int _F[\![\phi ^{a}_{K^+} ]\!] \cdot \{\! \!\{ {\varvec{\nabla }}\phi ^{b}_{K^-}\}\! \!\} + \gamma |F|^{-1} \int _F[\![\phi ^{a}_{K^+} ]\!] \cdot [\![\phi ^{b}_{K^-} ]\!], \end{aligned}$$

with indices *a* and *b* ranging from 1 to the number of shape functions ${\mathcal {N}}_\Sigma$ of $K^+$ and $K^-$, respectively.

### Facet Integration

As mentioned in Sect. 3.7 for the case of cell integrals, facet integrals involved in the computation of the facet-wise matrix (22) cannot be in general computed analytically. These are instead computed using quadrature rules. In general, the bilinear form that contains the facet terms can be stated as

$$\begin{aligned} {a}_{F}({\phi ^{b}_{K^{+}}},{\psi ^{a}_{K^{-}}}) = \int _F \varvec{\mathcal {F}}(\varvec{x}) \mathrm{d}F. \end{aligned}$$

We can consider a reference facet ${\hat{F}}$, and a mapping $\varvec{\Phi }_F : {\hat{F}} \rightarrow F$ from the reference to the physical space. Let us represent the Jacobian of the geometrical mapping with $\varvec{J}_F\, \doteq \, \frac{\partial {\varvec{\Phi }}_F}{\partial \varvec{x}}$, which has values in $\mathbb {R}^{(d-1) \times d}$. We can rewrite the facet integral in the reference facet, and next consider a quadrature rule $\mathrm{Q}$ on ${\hat{F}}$ defined by a set of points/weights $(\hat{\varvec{x}}_\mathrm{gp}, \mathrm{w}_\mathrm{gp})$, as follows:

23 $$\begin{aligned} \left( \mathbf {A}^{F}_{K^+ K^-}\right) _{ab}&= \int _F \varvec{\mathcal {F}}(\varvec{x}) \mathrm{d} \Omega = \int _{\hat{F}} \varvec{\mathcal {F}}\circ \varvec{\Phi }_F(\varvec{x}) |\varvec{J}_F| \mathrm{d}F \nonumber \\&= \sum _{{\hat{\varvec{x}}_\mathrm{gp} \in \mathrm {Q}}} \varvec{\mathcal {F}}\circ \varvec{\Phi }_F(\hat{\varvec{x}}_\mathrm{gp}) \mathrm{w}(\hat{\varvec{x}}_\mathrm{gp}) |\varvec{J}_F(\hat{\varvec{x}}_\mathrm{gp})|. \end{aligned}$$

$|\varvec{J}_F|$ is defined as:

24 $$\begin{aligned} |\varvec{J}_F|=\left\| \frac{\mathrm{d}\varvec{\Phi }_F}{\mathrm{d} x} \right\| _2 \ \text{ and } \ |\varvec{J}_F|=\left\| \frac{\partial \varvec{\Phi }^1_F}{\partial \hat{\varvec{x}}} \times \frac{\partial \varvec{\Phi }_F^2}{\partial \hat{\varvec{x}}} \right\| _2, \end{aligned}$$

for $$d=2,3$$, respectively.

The expression of the shape functions and their gradients in the physical space in terms of the ones in the reference space are computed by using the cell-wise maps. Thus, two mappings $\varvec{\Phi }_{K^+}$ and $\varvec{\Phi }_{K^-}$ among the reference cell ${\hat{K}}$ and the cells $K^+$ and $K^-$ in physical space, respectively, are involved in the numerical evaluation of interior facet integrals. We can also consider the reference facet ${\hat{F}}$ and a map $\varvec{\Phi }_F$ from this reference facet to *F* (analogously as $\varvec{\Phi }_K$ and *K* but in one dimension less in the reference space). We can define a quadrature rule $(\hat{\varvec{x}}_\mathrm{gp},\mathrm{w}_\mathrm{gp})$ in ${\hat{F}}$. We can also define the reference facet ${\hat{F}}^\pm$ of ${\hat{K}}$ such that $\varvec{\Phi }_{K^\pm }( {\hat{F}}^\pm ) = F$, and the map $\varvec{\Phi }_{\hat{F}^\pm }$ from ${\hat{F}}$ to ${\hat{F}}^\pm$. With this map, we can define the quadrature $(\hat{\varvec{x}}_\mathrm{gp}^\pm \, \doteq \, \varvec{\Phi }_{\hat{F}^\pm }(\hat{\varvec{x}}_\mathrm{gp}), \mathrm{w}_\mathrm{gp})$ with respect to the reference cell ${\hat{K}}$.

However, the same facet *F* has (in general) a different orientation depending on the cell used as reference, and so, a different index might be assigned to the same facet quadrature points from the perspective of either cell, i.e., $\varvec{\Phi }_{K^+}(\hat{\varvec{x}}_\mathrm{gp}^+) \ne \varvec{\Phi }_{K^-}(\hat{\varvec{x}}_\mathrm{gp}^-)$ in general. We adopt the convention that facet quadrature points identifiers are in the local numbering space of $K^+$, and these local identifiers are translated into the local numbering space of $K^-$. This is represented by the permutation $\Pi (\mathrm{gp})$ such that

$$\begin{aligned} \varvec{\Phi }_{K ^-}(\hat{\varvec{x}}_{\Pi (\mathrm{gp})}^-) = \varvec{\Phi }_{K ^+}(\hat{\varvec{x}}_\mathrm{gp}^+) = \varvec{\Phi }_F(\hat{\varvec{x}}_\mathrm{gp}). \end{aligned}$$

**Fig. 2:**
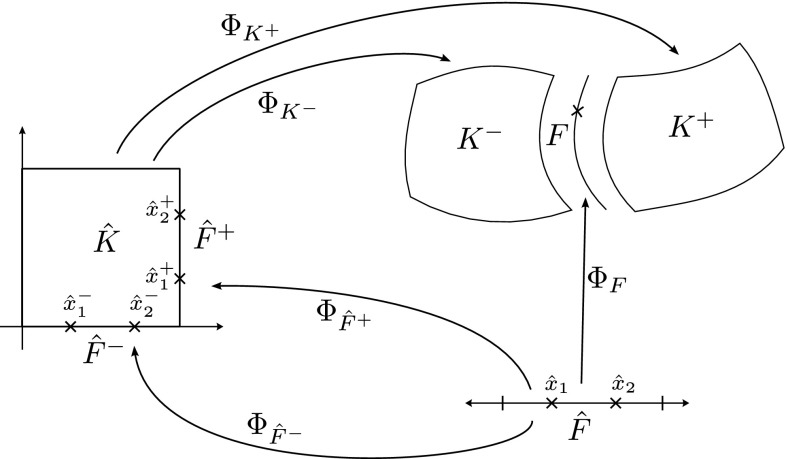
Mappings required for facet integration. The (only) quadrature point shown in the physical space is located at $\varvec{x}=\varvec{\Phi }_F(\hat{\varvec{x}}_\mathrm{1})=\varvec{\Phi }_{K ^+}(\hat{\varvec{x}}_\mathrm{1}^+) = \varvec{\Phi }_{K ^-}(\hat{\varvec{x}}_\mathrm{2}^-)$, that is, $\Pi (1)=2$ in this case

The logic underlying this translation is equivalent to the one discussed in Sect. 3.16; see Fig. 2 for an explanatory illustration. As a result, we have

$$\begin{aligned} \varvec{\Phi }_{K^{+}}^{-1} \circ \varvec{\Phi }_F (\hat{\varvec{x}}_{\mathrm{gp}}) = \hat{\varvec{x}}_{\mathrm{gp}}^+, \qquad \hbox {and} \qquad \varvec{\Phi }_{K^{-}}^{-1} \circ \varvec{\Phi }_F (\hat{\varvec{x}}_{\mathrm{gp}}) = \hat{\varvec{x}}_{\Pi (\mathrm{gp})}^-. \end{aligned}$$

Let us consider the evaluation of zero and first order derivatives on facets, i.e., the evaluation of $\phi ^{b}_{K^\alpha } \circ \varvec{\Phi }_{K^{\alpha }}(\hat{\varvec{x}}_\mathrm{gp})$ and ${\varvec{\nabla }}\phi ^{b}_{K^\alpha } \circ \varvec{\Phi }_{K^{\alpha }}(\hat{\varvec{x}}_\mathrm{gp})$ for $\alpha \in \{+,-\}$, where the quadrature points belong to a quadrature in the reference facet ${\hat{F}}$. We note that the introduction of ${\hat{\Psi }}$ is not needed for non-conforming methods, since there is no continuity to be enforced, and we will consider it to be the identity operator for simplicity. The values of the shape functions (times the geometrical mapping) on the facet quadrature points is evaluated as follows:

$$\begin{aligned} \phi ^{b}_{K^{\alpha }} \circ \varvec{\Phi }_F (\hat{\varvec{x}}_\mathrm{gp})= \hat{\phi }^{b} \circ \varvec{\Phi }_{K^{\alpha }}^{-1} \circ \varvec{\Phi }_F (\hat{\varvec{x}}_\mathrm{gp}), \end{aligned}$$

whereas shape function gradients are computed as:

$$\begin{aligned} {\varvec{\nabla }}\phi ^{b}_{K^{\alpha }} \circ \varvec{\Phi }_F(\hat{\varvec{x}}_\mathrm{gp}) = {\varvec{\nabla }}_{\hat{\varvec{x}}} \hat{\phi }^{b}(\hat{\varvec{x}}_\mathrm{gp}) \varvec{J}_K^{-1}\circ \varvec{\Phi }_{K^{\alpha }}^{-1} \circ \varvec{\Phi }_F (\hat{\varvec{x}}_\mathrm{gp}). \end{aligned}$$

Without loss of generality, let us focus on the first integral in (22). Replacing the mean value and jump operators by their definition in (21), and taking into account that $\phi ^{b}_{K^-}$ and ${\varvec{\nabla }}\phi ^{a}_{K^+}$ vanish on $K^+$ and $K^-$ (by construction of $\mathcal {X}_h$ and $\mathcal {Y}_h$), respectively, we end up with the following integral to be computed numerically:

$$\begin{aligned} -\frac{1}{2} \int _F \phi ^{b}_{K^-} \varvec{n}^- \cdot {\varvec{\nabla }}\phi ^{a}_{K^+} \mathrm{d} F\ . \end{aligned}$$

This integral is first mapped back to the reference facet ${\hat{F}}\subset \mathbb {R}^{\text{ d-1 }}$, and then it is approximated by the following sum over quadrature points:

25 $$\begin{aligned} \begin{aligned}&-\frac{1}{2} \int _F(\phi ^{b}_{K^-} \varvec{n}^-) \cdot {\varvec{\nabla }}\phi ^{a}_{K^+} \mathrm{d} F\\&\quad = -\frac{1}{2} \int _{{\hat{F}}} \hat{\phi }^{b}_{K^-} \circ \varvec{\Phi }_{K^{-}}^{-1} \circ \varvec{\Phi }_F (\hat{\varvec{x}}_\mathrm{gp}) \varvec{n}^- \circ \varvec{\Phi }_F (\hat{\varvec{x}}_\mathrm{gp}) \cdot {\varvec{\nabla }}_{\hat{\varvec{x}}} \hat{\phi }^{b}(\hat{\varvec{x}}_\mathrm{gp}) \varvec{J}_K^{-1}\circ \varvec{\Phi }_{K^{+}}^{-1} \circ \varvec{\Phi }_F (\hat{\varvec{x}}_\mathrm{gp}) |\varvec{J}_F| \mathrm{d} {\hat{F}}\\&\quad \approx -\frac{1}{2} \sum _{gp \in \mathrm{Q} } \hat{\phi }^{b}_{K^-}(\hat{\varvec{x}}^{-}_{\Pi (\mathrm{gp})}) \varvec{n}^-(\varvec{x}_\mathrm{gp}) \cdot {\varvec{\nabla }}_{\hat{\varvec{x}}} \hat{\phi }^{b}(\hat{\varvec{x}}_\mathrm{gp}^+) \varvec{J}_K^{-1}(\hat{\varvec{x}}^{+}_{q}) |\varvec{J}_F(\hat{\varvec{x}}_\mathrm{gp})| \mathrm{w}_\mathrm{gp}. \end{aligned} \end{aligned}$$

Using these ideas, we can compute all the terms related to facet integrals. Furthermore, outward normals on facets can be computed as:

26 $$\begin{aligned} \varvec{n}^\alpha = (-1)^{o_\alpha } \frac{ \frac{\mathrm{d}\varvec{\Phi }_F}{\mathrm{d} x} }{\left\| \frac{\mathrm{d}\varvec{\Phi }_F}{\mathrm{d} x} \right\| _2 } \ \text{ and } \ \varvec{n}^\alpha = (-1)^{o_\alpha } \frac{ \frac{\partial \varvec{\Phi }^1_F}{\partial \hat{\varvec{x}}} \times \frac{\partial \varvec{\Phi }_F^2}{\partial \hat{\varvec{x}}} }{ \left\| \frac{\partial \varvec{\Phi }^1_F}{\partial \hat{\varvec{x}}} \times \frac{\partial \varvec{\Phi }_F^2}{\partial \hat{\varvec{x}}} \right\| _2 }, \end{aligned}$$

for $d=2,3$, respectively, and $\alpha \in \{+,-\}$; *o* is 0 or 1 and is used to enforce the normal to be outwards. Tangent vector(s) for a given facet can be easily computed out of the normal vector.

### Polytopes

One of the motivations of FEMPAR is to develop a framework that can deal with arbitrary space dimensions. It permits to readily implement space-time formulations, which are posed in 4D. Other higher-dimensional applications include systems of PDEs posed in the phase space, e.g., the 7D (including time) Vlasov-Maxwell equations for the simulation of plasmas.

In this section, we provide the mathematical abstraction of cell topologies based on the concept of *polytope*. This abstract concept is of practical importance, because it allows us to develop algorithms and codes that can be applied to any topology that fits into the framework. The framework developed herein is very general and includes triangles and quadrilaterals in 2D, and tetrahedra, hexahedra, prysms, and pyramids in 3D. Furthermore, it can also be extended to arbitrary dimensions, to define not only n-cubes and n-simplices but many other topologies. A polytope is mathematically defined as the convex hull of a finite set of points. As a consequence, a polytope is a polyhedron. In the frame of FEMPAR, we consider polytopes that can be expressed as the image of the composition of two operators. The definition of topologies for reference FEs based on this idea can be found in [25].

The main topological information consumed by FE codes is the description of the *d*-dim polytope boundary as the assemble of $(d-1)$-dim polytopes, proceeding recursively till 0-dim objects are obtained (vertices); we use the contraction *k*-*dim* object to say object of dimension *k*. These lower-dimensional entities describing the polytope boundary are denoted herein as *n-faces*. Usually, the nomenclature used to describe n-faces in FEs is restricted to 3D problems. In FEMPAR and in the following exposition, we use a dimension-independent nomenclature in order to accommodate higher-dimensional problems. We consider the space dimension $d \in \mathbb {N}^+$ and a *d*-dim polytope. We define the *d*-face as the polytope itself. The set of $(d-1)$-dim polygons that compose the boundary of the polytope are its $(d-1)$-faces; $(d-1)$-faces are usually denoted as *facets*. We can proceed recursively, i.e., defining the $(k-1)$-faces of the polytope as the set of facets of its *k*-faces till reaching 0-faces. In 3D, 3-faces are called *cells*, 2-dim faces are *faces*, 1-dim faces are *edges*, and 0-dim faces are *vertices*. Herein, we use the term n-faces to denote all these objects. In this work, we denote by *vefs* the set of n-faces of dimension lower than the space dimension, e.g., it only includes vertices, edges, and faces in 3D.

Let us introduce some notation. We represent the set of bitmaps of size *m* with $\mathbb {B}^m$. The bitmaps $(1,1,\ldots ,1)$ and $(0,0,\ldots ,0)$ are represented with ${\mathtt {1}}$ and ${\mathtt {0}}$, respectively. Given a domain $\square \subset \mathbb {R}^d$ we use the notation $\alpha \square + \varvec{b}$, $\alpha \in \mathbb {R}$, $\varvec{b}\in \mathbb {R}^d$ to denote the domain $\{ \alpha \varvec{x}+ \varvec{b}\, : \, \varvec{x}\in \square \}$. $\mathbf {\varvec{e}}_j$ represents the *j*-th canonical basis vector of $\mathbb {R}^d$.

Let us define first the *directional extrusion* ${\square }_{(j;\alpha ,\beta )}$ of $\square$ with respect to the direction ${\mathbf {\varvec{e}}_j}$ of type $(\alpha ,\beta )$. $\alpha$ determines the topology of the extrusion, namely a prysm-type extrusion (1) or a pyramid-type extrusion (0) (see also [25]). $\beta$ determines whether we want to perform the $\alpha$-extrusion (1) or do-nothing (0). Based on this, we have the following definition.

#### **Definition 3.3**

(*Directional extrusion*) Given a domain $\square \subset \mathbb {R}^{d}$, we define ${\square }_{(j;\alpha ,\beta )} \subset \mathbb {R}^{d}$, with $\beta , \, \alpha \in \{0,1\}$ and $j = 1, \ldots , d$, as

$$\begin{aligned} {\square }_{(j;\alpha ,0)} \, \doteq \, \square , \qquad {\square }_{(j;0,1)} \, \doteq \, \{ (1-z)\square + z \mathbf {\varvec{e}}_j \, : \, z \in [0,1] \}, \qquad {\square }_{(j;1,1)} \, \doteq \, \{ \square + z \mathbf {\varvec{e}}_j : \, z \in [0,1] \}. \end{aligned}$$

**Fig. 3:**
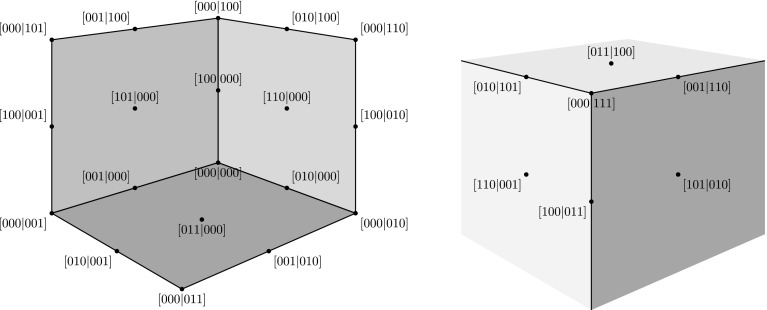
${\mathtt {e}}$ and ${\mathtt {v}}$ values for all the n-faces (with the exception of the volume) of the 3-cube, with topology ${\mathtt {t}}= (111)$

**Fig. 4:**
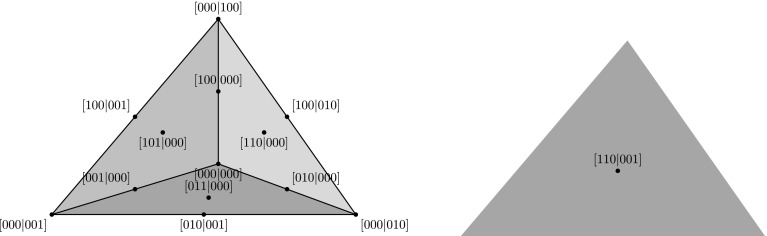
${\mathtt {e}}$ and ${\mathtt {v}}$ values for all the n-faces (with the exception of the volume) of the 3-simplex, with topology ${\mathtt {t}}= (000)$

The directional extrusion can be used recursively to define polytopes and their n-faces. An n-face is determined by a topology ${\mathtt {t}}\in \mathbb {B}^d$, an extrusion ${\mathtt {e}}\in \mathbb {B}^d$, and an anchor vertex $\varvec{v}\in \mathbb {R}^d$, using a recursive procedure as follows. The use of directional extrusions to get different polytopes and n-faces is illustrated in Figs. 3 and 4. One can observe how all the lower dimensional n-faces after directional extrusion lead to one dimension larger n-faces for different values of $\alpha$.

#### **Definition 3.4**

(*n-face*) Given ${\mathtt {t}}, \, {\mathtt {e}}\in \mathbb {B}^d$ and $\varvec{v}\in \mathbb {R}^d$, we can define the n-face $\square$ in a recursive way as follows. Let $\square ^0 \, \doteq \, \{ \varvec{v}\}$; we define $\square \, \doteq \, \square ^d$ based on the following recursion:

27 $$\begin{aligned} \square ^0 \rightarrow \square ^1 \, \doteq \, {\square ^0}_{(1;{\mathtt {t}}(0),{\mathtt {e}}(0))} \rightarrow \ldots \rightarrow \square ^{i+1} \, \doteq \, {\square ^{i}}_{(i+1;{\mathtt {t}}(i),{\mathtt {e}}(i))} \rightarrow \ldots \rightarrow \square ^d \, \doteq \, {\square ^{d-1}}_{(d;{\mathtt {t}}(d-1),{\mathtt {e}}(d-1))}. \end{aligned}$$

For our purposes, the anchor vertex $\varvec{v}$ has only 0/1 entries, and thus, it can be represented as an element ${\mathtt {v}}$ of $\mathbb {B}^d$. As a result, an n-face can be uniquely represented with $({\mathtt {t}};{\mathtt {e}},{\mathtt {v}})$. Based on this definition, we can define a set of *d*-dim polytopes by recursion. *d*-dim polytopes are given by ${\mathtt {t}}$, and represented as n-faces with $({\mathtt {t}}, {\mathtt {1}}, {\mathtt {0}})$, i.e., using the origin $\varvec{0}$ as anchor vertex and performing extrusions in all directions. On the other hand, a vertex $\varvec{v}$ (with only 0/1 coordinates) is an n-face with $({\mathtt {t}},{\mathtt {0}},{\mathtt {v}})$. Some examples of n-face constructions using this procedure can be found in Figs. 3 and 4. Furthermore, in these figures we show all n-faces of the 3-cube and 3-simplex, with all the ${\mathtt {e}}$ and ${\mathtt {v}}$ values. In our implementation of polytopes, we use Hasse diagrams based on the composition of extrusion and anchor vertex bitmaps to label the different n-faces of a polytope.

In codes, like in FEMPAR , the topology can be coded with the bitmap ${\mathtt {t}}$ (e.g., one 32-bit integer). FEMPAR can use any geometry that can be defined this way, for an arbitrary space dimension. This polytope definition leads to the following geometries. The 1-dim line segment topology is ${\mathtt {t}}= (0)$ or (1); this ambiguity in 1D is inherited to higher dimensions. In 2D, the triangle topology is ${\mathtt {t}}= (00)$ (or (01)) and the quadrilateral topology ${\mathtt {t}}= (10)$ (or (11)). In 3D, cubes are represented by ${\mathtt {t}}= (1,1,0)$ (or (1, 1, 1)), tetrahedra ${\mathtt {t}}= (0,0,0)$ (or (0,0,1)), prysms by ${\mathtt {t}}=(1,0,0)$ (or (1,1,1)), and pyramids by ${\mathtt {t}}=(0,1,0)$ (or (0,1,1)). Cosserats in 4D are represented by ${\mathtt {t}}=(1,1,1,0)$ (or (1,1,1,1)). In general, $2^{k-1}$ types of *k*-dim topologies are possible. n-cubes are expressed by ${\mathtt {t}}= {\mathtt {1}}$ and n-simplices by ${\mathtt {t}}= {\mathtt {0}}$.

Given a bitmap ${\mathtt {t}}$ and a bit $\alpha$, we define the bit operation that modifies the *j* bit of ${\mathtt {t}}$ to $\alpha$ with ${\mathtt {t}}.o_j(\alpha )$. Given the chain on n-faces (27), let us assume that $\square _{i-1}$ is represented by $({\mathtt {t}},{\mathtt {e}}',{\mathtt {v}})$. The extrusion $\square _{i} \, \doteq \, {\square _{i-1}}_{(i;*,\alpha )}$ is defined by $({\mathtt {t}},{\mathtt {e}}'.o_{i-1}(\alpha ),{\mathtt {v}})$. Thus, the chain (27) can be represented as follows. Given a topology ${\mathtt {t}}$, an extrusion ${\mathtt {e}}$, and an anchor vertex ${\mathtt {v}}$, we start with $({\mathtt {t}},{\mathtt {e}}',{\mathtt {v}}) \, \doteq \, ({\mathtt {t}},{\mathtt {0}},{\mathtt {v}})$ and proceed recursively:

28 $$\begin{aligned} ({\mathtt {t}},{\mathtt {e}}',{\mathtt {v}})&\rightarrow ({\mathtt {t}},{\mathtt {e}}'.o_0({\mathtt {e}}(0)),{\mathtt {v}}) \rightarrow \ldots \nonumber \nonumber \\&\rightarrow ({\mathtt {t}},{\mathtt {e}}'.o_{i}({\mathtt {e}}(i)),{\mathtt {v}}) \rightarrow \ldots \rightarrow ({\mathtt {t}},{\mathtt {e}}'.o_{d-1}({\mathtt {e}}(d-1)),{\mathtt {v}}) \equiv ({\mathtt {t}},{\mathtt {e}},{\mathtt {v}}). \end{aligned}$$

E.g., in 3D, the polytope itself (or 3-face) is determined by ${\mathtt {t}}= (1,1,1)$ and $({\mathtt {e}},{\mathtt {v}}) = ((1,1,1),(0,0,0))$. The chain (28) in this case reads as follows: (We omit ${\mathtt {t}}$ in the chain since it is the same for all elements in the recursion.)

$$\begin{aligned} ((0,0,0),(0,0,0)) \rightarrow ((0,0,1),(0,0,0)) \rightarrow ((0,1,1),(0,0,0)) \rightarrow ((1,1,1),(0,0,0)). \end{aligned}$$

Using the definition of the n-face, every element of the chain has a geometrical representation. We start with the vertex $\varvec{0}$, next obtain the line segment $\{(x,0,0) \, : \, x \in [0,1]\}$, next the square $\{(x,y,0) \, : \, x, \, y \in [0,1]\}$, and finally the unit cube. The previous definition is not only useful to represent *d*-dim objects *but all its n-faces*. See Figs. 3 and 4.

For a given n-face $\square \equiv ({\mathtt {t}},{\mathtt {e}},{\mathtt {v}})$, we want to define the set $\mathcal {S}_{\square }$ of all n-faces of $\square$. In order to do so, we introduce the following concepts.

#### **Definition 3.5**

(*Oriented set extrusion*) Given a set $\mathcal {S} = \{ \square : \square \in \mathbb {R}^d \}$, we define ${\mathcal {S}}_{(j;\alpha ,\beta )}$, with $\beta , \, \alpha \in \{0,1\}$ and $j = 1, \ldots , d$ as:

$$\begin{aligned} {\mathcal {S}}_{(j;\alpha ,0)} \, \doteq \, \mathcal {S}, \qquad {\mathcal {S}}_{(j;0,1)} \, \doteq \, \{ \square , \varvec{0}+{\mathbf {\varvec{e}}_j}, {\square }_{(j;0,1)} \, : \, \square \in \mathcal {S} \}, \qquad {\mathcal {S}}_{(j;1,1)} \, \doteq \, \{ \square , \square + {\mathbf {\varvec{e}}_j}, {\square }_{(j;1,1)} \, : \, \square \in \mathcal {S} \}. \end{aligned}$$

#### **Definition 3.6**

(*Set of n-faces*) Given an n-face $({\mathtt {t}},{\mathtt {e}},{\mathtt {v}})$, we can obtain all its n-faces recursively as follows. Let $\mathcal {S}^0 \, \doteq \, \{ \varvec{v}\}$; we define $\mathcal {S} \, \doteq \, \mathcal {S}^{d}$ based on the following recursion:

29 $$\begin{aligned} \mathcal {S}^0 \rightarrow \mathcal {S}^1&\, \doteq \, {\mathcal {S}^0}_{(1;{\mathtt {t}}(0),{\mathtt {e}}(0))} \rightarrow \ldots \rightarrow \mathcal {S}^{i+1} \nonumber \\&\, \doteq \, {\mathcal {S}^i}_{(i+1;{\mathtt {t}}(i),{\mathtt {e}}(i))} \rightarrow \ldots \rightarrow \mathcal {S}^{d} \, \doteq \, {\mathcal {S}^{d-1}}_{(d;{\mathtt {t}}(d-1),{\mathtt {e}}(d-1))}. \end{aligned}$$

All the resulting n-faces can also be written with the $({\mathtt {t}},{\mathtt {e}},{\mathtt {v}})$ notation commented above. In order to define this chain as in (28) (i.e., only based on the bitmap notation), we note the following. Given the n-face $\square \equiv ({\mathtt {t}},{\mathtt {e}},{\mathtt {v}})$, the n-face $\square + {\mathbf {\varvec{e}}_j}\equiv ({\mathtt {t}},{\mathtt {e}},{\mathtt {v}}.o_j(1))$. With this ingredient, we can implement the generator of all n-faces of an n-face using the bitmap notation.

We also want to know the facets of an n-face. We use the following statement. Given an n-face $\square \equiv ({\mathtt {t}},{\mathtt {e}},{\mathtt {v}})$ and its corresponding chain (28), the *i*-th element boundary $\partial \square ^{i} \, \doteq \, \partial {\square ^{i-1}}_{(i;{\mathtt {t}}(i-1),{\mathtt {e}}(i-1))}$ is the following:

30 $$\begin{aligned} \partial \square ^i&= \partial \square ^{i-1}, \hbox { if } {\mathtt {e}}(i-1) = 0,\nonumber \\ \partial \square ^i&= \{ \square ^{i-1}, \partial {\square ^{i-1}}_{(i;0,1)} \}, \hbox { if } {\mathtt {t}}(i-1) = 0, \, {\mathtt {e}}(i-1) = 1, \nonumber \\ \partial \square ^i&= \{ \square ^{i-1}, \square ^{i-1} + {\hat{\mathbf{{e}}}}_i, \partial {\square ^{i-1}}_{(i;1,1)} \} \hbox { if } {\mathtt {t}}(i-1) = 1, \, {\mathtt {e}}(i-1) = 1, \end{aligned}$$

with $$\partial \square ^1 = \{ \square ^0, \square ^0 + {\hat{\mathbf{{e}}}}_1 \}$$.

Using this definition of facets for the 3D cube, we get the following faces: ((1, 1, 0); (0, 0, 0)) and ((1, 1, 0); (0, 0, 1)) faces ($x=0$ and $x=1$ faces), ((1, 0, 1); (0, 0, 0)) and ((1, 0, 1); (0, 1, 0)) faces ($y=0$ and $y=1$ faces), ((0, 1, 1); (0, 0, 0)) and ((0, 1, 1); (1, 0, 0)) faces ($z=0$ and $z=1$ faces), having 6 faces in total. For every one of these faces, we can use the same definition above, to obtain the $(d-2)$-faces that are in the boundary of every $(d-1)$-face. All these ideas can be used for any polytope, not only n-cubes. The only difference is the type of extrusion being used in every case.

### Node Generation and Indexing

FE spaces are polynomial spaces, e.g., Lagrangian polynomials. (Let us note that div- and curl-conforming FEs also rely on Lagrangian polynomials for the definition of the pre-bases and the definition of the equivalence classes.) In order to express these polynomials, one must define a set of points (nodes). In the following, we define a node generator for a given order on an arbitrary polytope, using lexicographical notation.^8^

#### **Definition 3.7**

(*Set of nodes*) Let us consider a polytope $\square \in \mathbb {R}^d$ represented by $({\mathtt {t}},{\mathtt {1}},{\mathtt {0}})$. Its set ${\mathcal {N}}^k$ of equidistant Lagrangian nodes of order *k*, in lexicographical notation, are generated recursively as follows: ${\mathcal {N}}^k \, \doteq \, {\mathcal {N}}_{d}^k$, where

31 $$\begin{aligned} {\mathcal {N}}_{m+1}^p = \{ ({\varvec{\alpha }},\beta ) : {\varvec{\alpha }}\in {\mathcal {N}}_{m}^{p- \beta (1-{\mathtt {t}}(m))}, \beta \in {\mathcal {N}}_1^p \}, \quad \hbox {with } \, {\mathcal {N}}_1^q = \{ \alpha \in \mathbb {N}^+ : \alpha \le q \}. \end{aligned}$$

Given a node ${\varvec{\alpha }}\in \mathbb {N}^d$ in lexicographical notation and assuming an equidistant distribution of nodes, its space coordinates $x_{\varvec{\alpha }}\in \mathbb {R}^d$ can readily be obtained, $x_{\varvec{\alpha }}\, \doteq \, {\varvec{\alpha }}/k$. We note that for n-cubes we recover the typical tensor product definition of nodes and the corresponding truncated subset of nodes for n-simplices. Other node generators can also be considered, especially for very high-order elements (e.g., Fekete points).

It is basic in FE analysis to have an *ownership* relation between n-faces and nodes. In particular, it is basic to enforce continuity between FEs by enforcing continuity of nodal values. In order to generate the nodes of the polytope that belong to an n-face, we use the following construction.

First, we generate the local set of nodes, using the definition above, for the n-face. Given a *k*-face $({\mathtt {t}},{\mathtt {e}},{\mathtt {v}})$ in $\mathbb {R}^d$, we consider the *reference* *k*-dim polytope $({\mathtt {t}}',{\mathtt {1}},{\mathtt {0}})$, where ${\mathtt {t}}'$ is the restriction of ${\mathtt {t}}$ to the components that are extruded, i.e., ${\mathtt {t}}' \, \doteq \, {\mathtt {t}}\circ m_{\ell g}$ with the mapping $m_{\ell g} : \{1, \ldots , k\} \rightarrow \{ { j \in \{1, \ldots , d\}: {\mathtt {e}}(j) = 1} \}$. Next, we define the local nodes of the n-face as the nodes of the reference polytope. It defines the n-faces nodes and their local coordinates. Finally, we define the linear mapping from the reference *k*-dim polytope to the *k*-face. The map can be defined with $k+1$ independent conditions. It can be defined by enforcing that the mapping maps the anchor vertex of the reference polytope to the one of the *k*-face and the same for the extrusion of the anchor vertex to all directions:

$$\begin{aligned} m(\varvec{0}) = \varvec{v}, \qquad m({\mathbf {\varvec{e}}_s}) = \mathbf {\varvec{e}}_{m_{\ell g}(s)}, \, \hbox { if } {\mathtt {t}}'(s) = 0, \, \qquad m({\mathbf {\varvec{e}}_s}) = \varvec{v}+ \mathbf {\varvec{e}}_{m_{\ell g}(s)}, \, \hbox { if } {\mathtt {t}}'(s) = 1. \end{aligned}$$

Since the mapping is linear, it can be written as:

$$\begin{aligned} m(\varvec{x}) = \varvec{\alpha }_0 + x_1 \varvec{\alpha }_1 + \ldots + x_k \varvec{\alpha }_k. \end{aligned}$$

Form the first constraints we get that $\varvec{\alpha }_0 = \varvec{v}$. For the other constraints, we get:

$$\begin{aligned} m({\mathbf {\varvec{e}}_s}) = \varvec{v}{\mathtt {t}}'(s) + \varvec{e}_{m_{\ell g}(s)} = \varvec{v}+ \varvec{\alpha }_s \longrightarrow \varvec{\alpha }_s = \varvec{v}({\mathtt {t}}'(s)-1) + \varvec{e}_{m_{\ell g}(s)}. \end{aligned}$$

Thus, we get:

32 $$\begin{aligned} m(\varvec{x}) = \varvec{v}+ \sum _{s=1}^k x_s \varvec{v}({\mathtt {t}}'(s)-1) + x_s \varvec{e}_{m_{\ell g}(s)} + x_s, \end{aligned}$$

and thus:

$$\begin{aligned} m(\varvec{x})_i = v_i (1 - \sum _{\begin{array}{c} \{ s = 1, \ldots , k : \\ {\mathtt {t}}'(s) = 0 \} \end{array} } x_s) + x_{m_{\ell g}^{-1}(i)}. \end{aligned}$$

We could also obtain the expression for the inverse of the mapping *m* analogously. We can readily use the mapping for lexicographical coordinates. As a result, given a *k*-face, we can define its nodes with a local numbering based on the lexicographical label of the reference *k*-face. The local-to-global lexicographical label (where global is the label of the *d*-dim base polytope) is obtained by applying the mapping (32).

### Global DOF Numbering and Conformity

A basic ingredient in FE analysis is the imposition of continuity among FEs in order to build conforming global FE spaces. This process is mathematically defined with equivalence classes on DOFs (see Sect. 3.6). For example, functions in the Lagrangian FE space are related to geometrical nodes, and to impose continuity of a function among FEs is equivalent to impose continuity of nodal values in the same spatial position (see Sect. 3.8). In the following, we provide a mechanism to identify nodes in two different cells that share the same position to implement the required equivalence class. The situation is slightly more involved for div-conforming and curl-conforming FE spaces. In these cases, one can still determine a DOF with a node plus n-face ownership (see Sects. 3.9 and 3.10, respectively). Thus, the equivalence class in these situations can be formulated as in Lagrangian FEs (determine nodes with the same position) at every n-face separately.

**Fig. 5:**
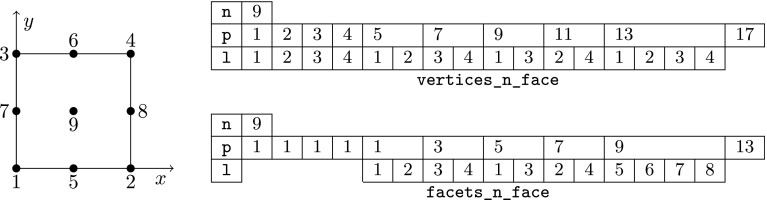
Numbering convention for n-faces with ${\hat{K}}$ a quadrilateral (left) and the status of vertices_n_face and facets_n_face corresponding to that numbering (right). n, p(n+1), and l(p(n+1)-1) are private member variables of type(list_t) storing the number of entities, the start position in l(:) of the list associated to each entity, and the identifiers associated of all lists gathered in a single array, respectively

**Fig. 6:**
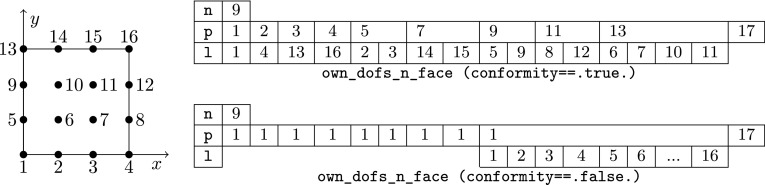
Numbering convention for the DOFs of an (scalar-valued) bi-cubic Lagrangian FE on top of a quadrilateral (left) and the status of own_dofs_n_face for this reference_fe_t in its CG (right, top) and DG forms (right, bottom)

Following Sect. 3.6, a node within a cell of our triangulation can be represented as (*b*, *K*), where *b* is the local cell-wise index of the node and *K* is the cell global index. Given an n-face *F* of the cell, the same node can be represented with $(b',F,K)$, where $b'$ is an n-face-based local index. For example, node 8 (cell-wise local index) in the cell of Fig. 6 can also be determined as the node 1 (facet-wise local index) of the n-face 8 (see Fig. 5). This facet-wise local index is determined by the coordinate system being used at the n-face. For example, the nodes of n-face 8 in Fig. 6 are ordered as (8, 12) (i.e., first 8 and then 12). On the other hand, node indices are represented with the coordinates in a lexicographical coordinate system, as presented in (31). For example, node with $b=8$ ($b'=1$ in n-face 8) is represented with the coordinates $\varvec{s}=(4,1)$ ($\varvec{s}'=(1)$ in the n-face).

Let us consider an n-face *F* in our triangulation, two cells $K^{+}$ (source cell) and $K^{-}$ (target cell) sharing the n-face, and nodes $(\varvec{s}_+',F,K^{+})$ and $(\varvec{s}'^{-},F,K^{-})$ (with n-face-wise local indices). The question that must be answered is: are nodes $(\varvec{s}_+',F,K^{+})$ and $(\varvec{s}'^{-},F,K^{-})$ in the same spatial position? This question can be answered with the map $\mathtt {p}_F$ in (15) that, given the position of the node in the coordinate system of *F* in $K^{+}$, provides the one in $K^{-}$.

We note that this mapping is trivial when using structured (possibly locally adapted) n-cube meshes, since the local ordering of nodes in an n-face based on increasing local index leads to the same ordering for all cells containing that n-face; we say that the mesh is *properly oriented* in this case. However, 2D or 3D unstructured mesh generators might not return properly oriented meshes, and thus the FE code has to deal with the explicit construction and application of permutations. We also note that one can always end up with oriented meshes for n-simplices by simple cell-wise permutations (see, e.g., [72, Sect. 5.5] and [80]). After reading n-simplex meshes, these meshes are always properly oriented in FEMPAR before proceeding to any computation. While this is also true for 2D n-cube meshes, 3D n-cube meshes cannot be properly oriented in general [81].

Let us consider the reference polytope ${\hat{K}}$ associated to $K^{+}$ and $K^{-}$. In general, the n-face *F* has a different n-face local index with respect to the two cells; its corresponding reference n-face is represented with ${\hat{F}}^+$ and ${\hat{F}}^{-}$ for $K^{+}$ and $K^{-}$, respectively. In general, the map between nodes of these two n-faces can be defined by using (32), which is invertible (since it is linear and full rank). Using this approach, the map can be generated for arbitrary dimension and polytope topology. However, for the particular case of 2D/3D n-cube meshes, we have implemented this procedure in a more computationally efficient manner. In particular, the required permutations (mappings) are expressed in terms of a set of tables, which are stored and set up (filled) by the so-called reference_fe_t abstract data type in FEMPAR. We refer to Sect. 6.3 for detailed implementation details. (Recall that n-simplices meshes do not actually require this procedure as they can always be properly (re)oriented.)

Let us consider the case of 3D n-cube meshes. Vertices are trivial because there is only one node and no permutation is needed. For edges and faces, we rely on the three following concepts:

- Rotation index: Provides the local index of the anchor vertex of $F^{-}$ with respect to the coordinate system of $F_+$. When FEs are sharing two edges, we have the following situations. The edge can have the same anchor vertex seen from both elements, or not. For faces, the anchor vertex can be in 4 positions. It is called *rotation* because it represents a map that keeps invariant the reference face ${\hat{F}}^{-}$ and makes the anchor vertices of the source and target cells coincide.
- Orientation index: Given two cells sharing an n-face with the same anchor vertex, the orientation index codes the map from the coordinate system of the n-face with respect to the first cell to the one with respect to the second one.^9^ For edges, this map is always the identity, because two cells sharing an edge with the same anchor node provide the same edge-wise node coordinates to its nodes. For faces, the situation is more complex, because it involves 2 different possible situations. The orientation index is equal to 0 for the identity permutation and 1 when we have to swap indices. We denote the base face as the face with the lowest local index (face [011|000] in Fig. 3). Next, we consider two cubes that share a face, restricted to the following scenario: (1) the face is the base face in at least one of the cubes; (2) the face has the same anchor vertex in the two cubes. It is trivial to compute the orientation index in these cases. The orientation index in the more general case of two cubes sharing a face only restricted to (2), i.e., two arbitrary faces with the same anchor vertex, can be obtained by composition as follows. If two faces have the same orientation index with the base face, they have an orientation index equal to 1, and 0 otherwise.
- Permutation index: An index obtained by composition of the rotation and orientation indices (i.e., it ranges from 1 and 2, and 1 and 8 for edges and faces, respectively), that codifies the final mapping between coordinates of two cells as the composition of a rotation and a orientation map. We note that the composition of all possible rotations and orientations cover all the possible relative positions of cells for a conforming mesh.

## Implementation of polytope_t and Related Data Types

In FEMPAR, the reference FE cell geometry is defined by the polytope_t data type; see Listing 1. The input needed to define the polytope is the space dimension num_dimensions and the topology ${\mathtt {t}}$ in the 32-bit integer topology.

**Figure:**
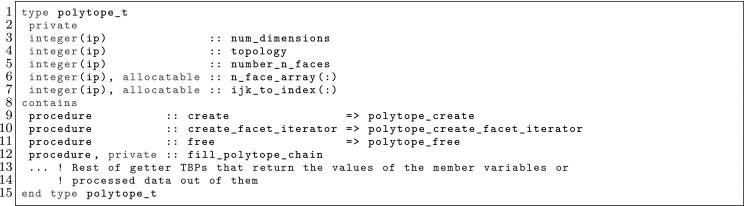
Listing 1. The polytope_t data type.

Using the ideas in (27), (28), and (29), we create the set of all n-faces of the polytope $({\mathtt {t}},{\mathtt {e}},{\mathtt {v}})$ in the (private) fill_polytope_chain TBP, which is in turn invoked by the (public) create TBP. All n-faces of the polytope have the same topology, and can be uniquely determined by a 32-bit integer that represents the composition of $({\mathtt {e}},{\mathtt {v}})$. We note that the ordering of n-faces based on $({\mathtt {e}},{\mathtt {v}})$ mixes n-faces of different dimensions and it is non-consecutive in general. Thus, we consider an ordering based first on the n-face dimension, and next by $({\mathtt {e}},{\mathtt {v}})$. The set of all n-faces generated by the recursion (29) are stored in n_face_array, an array of size number_n_faces. This array in particular provides the $({\mathtt {e}},{\mathtt {v}})$ associated to each n-face. The inverse mapping (from $({\mathtt {e}},{\mathtt {v}})$ to the actual numbering) is stored in the ijk_to_index array.

It is also possible to iterate over facets of an n-face, based on (30). The create_facet_iterator TBP of polytope_t creates a facet_iterator_t instance for a given n-face. facet_iterator_t is defined in Listing 2. The n-face $({\mathtt {e}},{\mathtt {v}})$ is stored in root, the topology can be extracted from its polytope pointer member variable. The iterator over facets is described by two integers, component and coordinate, using the ideas in (30). The complexity of the traversal over facets is coded in facet_iterator_next and facet_iterator_has_finished.

**Figure:**
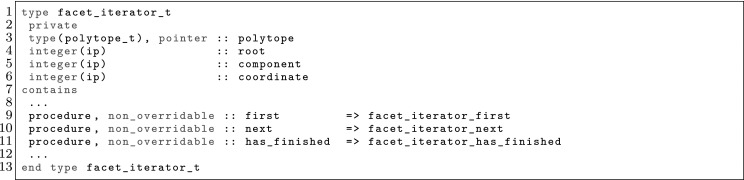
Listing 2. The facet_iterator_t data type.

With regard to the implementation of nodes within FEMPAR, we provide the node_array_t data type to represent the set of nodes defined in (31); see Listing 3. It is constructed from a polytope and the order. It provides a create TBP, where we perform (31) and fill all the resulting nodes in the node_array array member variable. We number the nodes using a consecutive numbering with increasing lexicographical index. The node array provides the lexicographical label in one integer. The inverse is stored in ijk_to_index. The total number of nodes is stored in num_nodes. Finally, the space coordinates of nodes are stored in coordinates.

**Figure:**
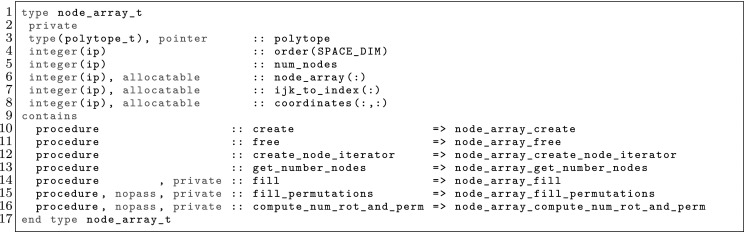
Listing 3. The node_array_t data type.

We also provide the node_iterator_t object (see Listing 4), which iterates over the nodes of an n-face (stored in n_face) using (31) and (32). It has a pointer to the node_array of the base polytope. Internally, it goes through the nodes of n_face (using (31)) (the current node being stored in displacement), which can be translated to the base polytope node numbering using (32) (stored in coordinate); the coordinate is computed on demand by calling the TBP node_iterator_current_ijk. The own_boundary logical allows one to iterate over the nodes considering the n-face as an open or closed set. We note that the create TBP of node_array_t relies on node_iterator_t.

**Figure:**
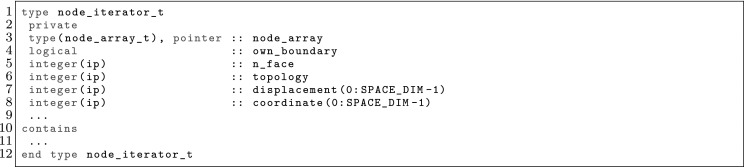
Listing 4. The node_iterator_t data type.

## The polynomial_t Abstraction

In FEMPAR, the definition of shape functions is not hard-coded, as usually done in most FE codes. Such approach has severe limitations: (1) it is not practical for high order discretizations, and the code cannot be written for an arbitrary order; (2) it involves a huge number of code lines with the analytical expression of shape functions for a given set of available orders (see the discussion in [82]); and (3) it does not allow for dimension-independent code. Instead, we consider a framework based on the concepts in Sect. 3.5, in which one considers a pre-basis, defines the moments, and performs a change of basis. The pre-basis is defined using the product of 1D functions (e.g., the Cartesian product), and the 1D function generator is written in terms of the (arbitrary) order. Our machinery for the generation of 1D functions has been restricted for the moment to polynomial functions in one variable, namely Lagrangian polynomials, monomials, and B-splines, but the implementation can be extended to other choices. The product of 1D functions can be a Cartesian product of 1D Lagrange polynomials (or monomials), to define ${\mathcal {Q}}_{\varvec{k}}$ spaces on n-cubes, or a reduced combination of monomials to define ${\mathcal {P}}_k$ spaces on n-simplices.

The definition of the reference FE functional space relies on the polynomial_t data type in Listing 5, which represents a polynomial in one variable, i.e., $p(x) = \sum _{i=0}^k a_ix^k$. Thus, a 1D polynomial is defined in terms of its order *k* and a set of $k+1$ coefficients $\{a_i\}_{i=0}^k$, stored in order and the coefficients array, respectively. Different type extensions of polynomial_t have been considered so far, namely lagrange_polynomial_t and monomial_t. The first one generates a Lagrangian polynomial as in Sect. 3.4, in which the coefficients array has in its first order entries the coordinates of the nodes and in the last entry the coefficient $\frac{1}{ \Pi _{n \in {\mathcal {N}}_k \setminus \{m\} } (x_m - x_s) }$ in (7). The monomial_t extension represents $x^k$ where *k* is its order. It is just a trivial case of polynomial_t for optimization purposes that is uniquely defined by the order (the coefficients array is not needed). We also consider the polynomial_basis_t data type, which is just a set (array) of (polymorphic) polynomials.

**Figure:**
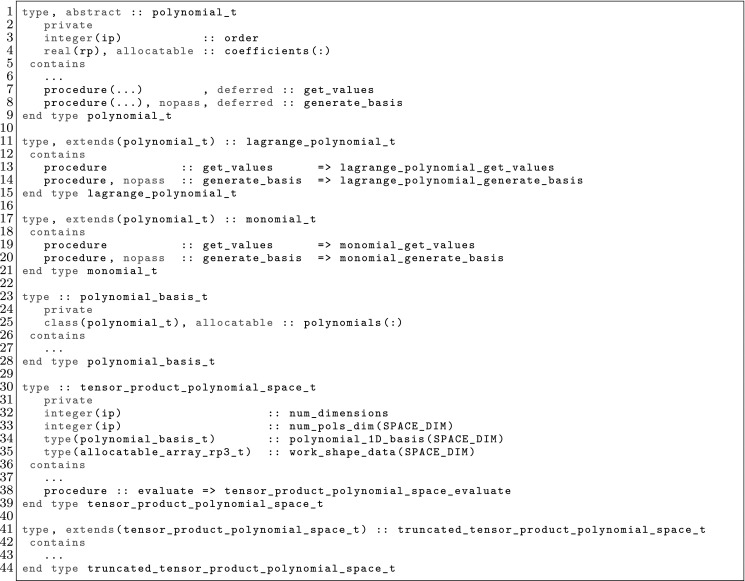
Listing 5. The polynomial_t data type and related data types.

Up to this point, we have defined Lagrange polynomials and monomials in one variable. lagrange_polynomial_t and monomial_t also provide the binding generate_basis that generates a Lagrangian and monomial basis of polynomials, for a given order *k*. The result of this subroutine is a polynomial_basis_t that includes as many polynomials as the polynomial space dimension. In the case of the Lagrangian basis, it implements the basis $\mathcal {L}^k$ in Sect. 3.4, whereas the binding for monomials simply implements $\{ x^i \}_{i=0}^k$.

The next step is to generate higher dimensional spaces. We consider two types of spaces. The first one is a space that can be generated as the Cartesian product of 1D spaces, implemented in the data type tensor_product_polynomial_space_t. This data type is defined through the number of space dimensions and as many polynomial_basis_t as space dimensions. This data type can be applied to any combination of 1D spaces. e.g., In the case of 1D Lagrange bases (possibly with different order and nodes per dimension), it leads to the multi-dimensional basis in (8). Thus, with this data type and Lagrangian 1D bases we generate the Lagrangian FE spaces on top of n-cube cells, i.e., the ${\mathcal {Q}}_{{\varvec{k}}}$ space of polynomials.^10^

Furthermore, we also consider the truncated_tensor_product_polynomial_space_t extension that generates Lagrangian FE spaces on n-simplices, i.e., the ${\mathcal {P}}_k$ space of polynomials. In this case, the generate_basis TBPs of monomial_t should be used to create the monomial 1D bases per direction and the order should also be the same for all directions. Otherwise, the resulting multi-variable function would have no sense. Next, the combination of 1D monomials only involves terms such that $| \varvec{\alpha }| \le k$ (see Sect. 3.4), to generate a pre-basis for FE spaces on tetrahedra, i.e., the ${\mathcal {P}}_k$ space of polynomials.

We note that with these abstract representations of polynomial spaces one can define the reference FE local space. However, unless one considers 1D Lagrangian basis and tensor product polynomials on n-cubes, the resulting basis is not the shape functions basis. Even in the case of Lagrangian n-simplices, a change-of-basis is needed, using the procedure in Sect. 3.5 taking nodal values as moments. In Sect. 9.5, we show how we can define the shape function basis for the case of div-conforming FEs of arbitrary order. The same ideas apply for grad-conforming Lagrangian FEs on n-simplices and curl-conforming FEs in general, but are not included for the sake of brevity.

## The reference_fe_t Abstraction

In this section, we introduce the reference_fe_t data type. This data type is the OO representation of the standard mathematical definition of a reference FE presented in Sect. 3.3, namely, a reference cell geometry ${\hat{K}}$, a functional space $\hat{\mathcal {V}}$, and a set of DOFs ${\hat{\Sigma }}$ defined on top of it. The reference_fe_t is a central abstraction in a FE library and must be judiciously designed to be extensible and reusable. In particular, it must not only accommodate Lagrangian FEs, but also other (more involved/general) spaces like Raviart-Thomas or edge FEs, DG methods, and B-spline patches. An extensible and reusable design of reference_fe_t should allow one to, e.g., easily incorporate new local functional spaces that were not originally considered, and to do so without having to rewrite (and thus recompile) any code that is grounded on the set of methods provided by reference_fe_t. To this end, in FEMPAR, reference_fe_t is an *abstract* data type that serves as a template equipped with a set of member variables and deferred bindings that subclasses have to set up and implement (i.e., override), respectively, in order to complete the description of the concrete FE space at hand. The definition of the reference_fe_t data type, a classification of its member variables into three different categories (corresponding to the three ingredients in Ciarlet’s definition), and an enumeration of its most relevant regular and deferred bindings, are shown in Listing 6.

**Figure:**
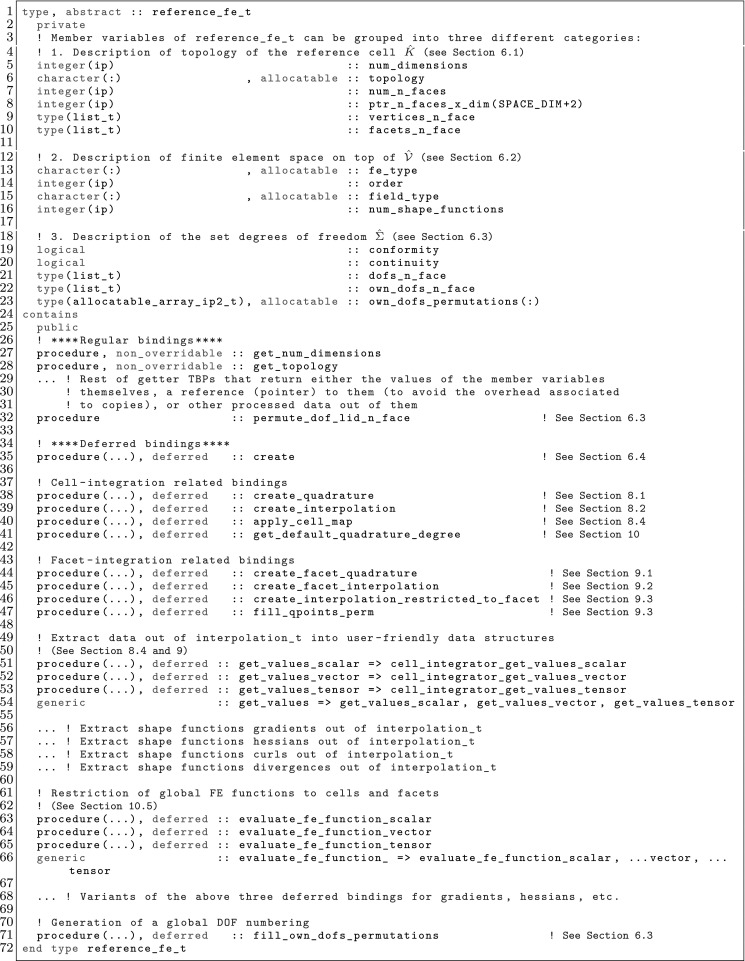
Listing 6. The reference_fe_t abstract type, a classification of its member variables, and an enumeration of its most relevant regular and deferred bindings.

This section is structured as follows. The member variables in each of the three aforementioned categories are covered in detail in Sects. 6.1–6.3, respectively. In Sect. 6.4, we discuss the OO design pattern chosen in FEMPAR for the creation of reference_fe_t polymorphic instances, and describe the arguments that uniquely define a subclass of this data type; these are in line with its mathematical definition. In Sect. 6.5, we enumerate and briefly describe the subclasses of reference_fe_t currently available in FEMPAR. We note that the section is not self-contained as most of the deferred bindings of reference_fe_t are not covered here. These involve interactions with other data types in our OO design, and will be described in the sections in which these interactions are exposed. Code comments in Listing 6 serve as a table of contents with the article sections in which these deferred bindings are covered.

### The Reference Cell Topology

The reference cell ${\hat{K}}$ is a polytope. Therefore, following Sect. 3.14, it can be described with the topology, coded as a set of *d* bits, where *d* is the dimension of the polytope. The reference cell topology is generated using polytope_t described in Sect. 4, which offers methods like composition and local numbering of n-faces. Polytope topologies include triangles and quadrilaterals in 2D, and tetrahedra, hexahedra, prysms, and pyramids in 3D. The member variables in charge of the description of the reference cell topology ${\hat{K}}$ are shown in Lines 5–10 of Listing 6. The user must provide the topology and dimension of the polytope to define ${\hat{K}}$, stored in the member variables topology and num_dimensions, respectively. A set of *getters* return this basic information, and other related data that can be generated out of them, e.g., the number of n-faces in the boundary of the cell is stored in the num_n_faces member variable. The list of vertex identifiers per each n-face and the list of facets (of dimension $n-1$) per each n-face are stored in vertices_n_face and facets_n_face, respectively; see Fig. 5 for an illustration of these member variables and the data type list_t used in FEMPAR to store and traverse lists.

The FEMPAR data type list_t stores a set of (variable-sized) lists of integer identifiers, one per each entity; in this particular scenario, entities are n-faces. As shown in Fig. 5, the current implementation of this data type uses a compressed storage layout as, e.g., in compressed storage formats for sparse graphs. In order to preserve encapsulation and data hiding, list_t offers a rich set of TBPs that lets users to set up (step by a step) a new list_t instance; this type also provides a list_iterator_t type that lets them to sequentially read/write each of the integer identifiers of the list associated to an entity. The code snippet in Listing 7 illustrates how to iterate and print the identifiers of those vertices belonging to the n-face with identifier n_face_lid.

**Figure:**
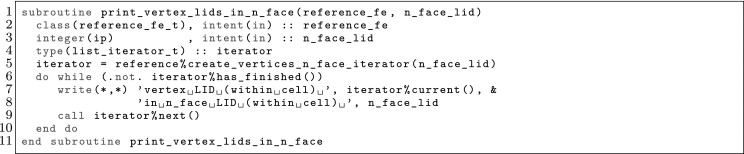
Listing 7. User-level code that illustrates how to print to screen those (local within cell) vertex identifiers belonging to n-face with (local within cell) identifier n_face_lid.

The number of n-faces of any dimension can be easily computed from ptr_n_faces_x_dim. We note that ptr_n_faces_x_dim is not a list_t instance, since we adopt the convention that n-faces are numbered from the lowest to highest dimension, and thus only the $\texttt {p}$ array of the list is actually needed (see Fig. 5). In the example in Fig. 5, the value of this array is $\{1,5,9,10\}$, since we have 4 vertices (dimension 0), 4 facets or edges (dimension 1), and 1 cell (dimension 2).

### The Reference FE Space

For a given cell topology, different definitions of functional spaces and sets of DOFs are possible, e.g., the ones of the nodal Lagrangian grad-conforming reference FE in Sect. 3.8, the Raviart-Thomas div-conforming reference FE in Sect. 3.9, or the curl-conforming Nédélec reference FE in Sect. 3.10. The member variables of reference_fe_t required to describe the functional space $\hat{\mathcal {V}}$ with support on ${\hat{K}}$ are encompassed within Lines 13–16 of Listing 6.

The local FE space $\hat{\mathcal {V}}$ is determined by the member variables fe_type, (in some cases) field_type, and order. fe_type uniquely identifies the concrete FE space at hand. Possible values are provided by means of the public parameter constants fe_type_lagrangian, fe_type_raviart_thomas, and fe_type_nedelec corresponding to the reference_fe_t implementors currently supported in FEMPAR; see Sect. 6.5 for additional details on those. field_type identifies the “type” of physical field being discretized, i.e., whether it is scalar, vector-valued, etc. There are FE spaces that are inherently vector-valued such as, e.g., Raviart-Thomas and edge FEs. However, Lagrangian FEs can be either used to discretize scalar, vector, or tensor-valued fields, and field_type must be provided. We assume that $\hat{\mathcal {V}}$ can be parameterized with respect to an order, which is stored in order. Out of these values, we can generate additional data, e.g., the number of shape functions is stored in num_shape_functions. For example, for (scalar-valued) bi-quadratic (2D) and tri-quadratic (3D) Lagrangian FEs, the field_type is scalar, num_components is equal to 1, order is equal to 2, and num_shape_functions is equal to 9 and 27, respectively.

### The Set of Local DOFs

Additional data is required to describe the set of DOFs ${\hat{\Sigma }}$ for $\hat{\mathcal {V}}$. In particular, the member variables encompassed within Lines 19–23 of Listing 6 serve this purpose.

The conformity member variable determines whether the global FE space $\mathcal {X}_h$ is conforming with respect to the infinite-dimensional space $\mathcal {X}$, i.e., whether $\mathcal {X}_h\subset \mathcal {X}$ or not. It is used to describe the n-face that owns every DOF, which is required to enforce conformity of the global FE space through equivalence classes (see Sect. 3). e.g., For Lagrangian FEs, setting it to .true. results in a grad-conforming global FE space, whereas setting it to .false. it results in a discontinuous space for DG methods. It is conceptually possible to set it to .true. on some cells and false on others, leading to the CDG method in [83]. On the other hand, the continuity member variable is only determined by $\mathcal {X}$, and tells us whether $\mathcal {X}$ admits a trace operator. Roughly speaking, it tells us whether we must enforce some type of continuity at the discrete level to preserve conformity, e.g., full, tangential, or normal traces for $H^1(\Omega )$, $H(\mathbf{curl},\Omega )$, and $H(\mathrm{div},\Omega )$, respectively. The value of continuity is .false. when $\mathcal {X}= L^2(\Omega )$, since no continuity is required. When continuity is .false., conformity must be .true.. continuity is barely used (see discussion in next paragraph).

The value of conformity is used to generate the own_dofs_n_face member variable of type list_t. This member variable stores, for every n-face, the DOFs it owns; see Fig. 6. For CG methods, the notion of ownership is related to the geometrical location. For DG FEs, although node functionals are still geometrically located on the boundary of the cell, they are nevertheless owned by the cell, and considered as interior DOFs, since there is no global conformity to be enforced. This array is heavily used to generate the global DOF numbering.^11^ On the other hand, the dofs_n_face member variable, determines, for a given n-face, the set of DOFs such that their respective shape functions are non-zero on the n-face. The continuity member variable is (currently) only used for DG methods in parallel distributed-memory environments. In particular, in order to decide whether to associate or not a global DOF identifier to nodes on the interface facets of ghost cells (and thus to be able to define non-singular sub-assembled matrices for the DD methods in [84] for DG discretizations). The dofs_n_face member variable is used when continuity is .true. and a global DOF numbering is to be generated, and also might be used by triangulation subclasses (see Sect. 7) in order to extract the coordinates of those nodes on top of a vertex, edge, or face (using the dofs_n_face member variable of the reference_fe_t instance that describes the geometry of the cell). For example, in Fig. 6, the list corresponding to n-face with identifier 8 in dofs_n_face is {4,8,12,16}.

The reference_fe_t data type plays a crucial role in the algorithm in charge of assigning global DOF identifiers to node functionals distributed over the interior of the triangulation cells and their boundary n-faces. (This algorithm, which is is covered in detail in Sect. 10, is grounded on the notion of equivalence classes introduced in Sect. 3.) In particular, the function-like (regular) binding referred to as permute_dof_lid_n_face (see Line 32 of Listing 6) implements the mapping $\mathtt {p}_F$ in (15). This function takes as input the so-called permutation index in Sect. 3.16, the local index of a node within an n-face of given dimension (e.g., in 3D, either 0 for vertices, 1 for edges, and 2 for faces) from the perspective of a source cell, and returns the local index of a node within that n-face from the perspective of the target cell.^12^ This is in particular the transformation that we have to apply when global DOF identifiers have been already assigned to n-face nodes in the source cell, and we want to transfer them to n-face nodes in the target cell; see Sect. 10.3. This binding, implemented in reference_fe_t, ultimately relies on its own_dof_permutations(:) member variable; see Line 23 in Listing 6. This allocatable array is indexed with the n-face dimension (i.e., 1 for edges, and 2 for faces). For each n-face dimension larger than 0, it contains a rank-2 allocatable array (i.e., type(allocatable_array_ip2_t) is the base type of the array), which serves as a lookup table for the implementation of the aforementioned transformation. In particular, the rows are indexed with the local index of the node identifier on top of the n-face from the perspective of the source cell, and the columns with the permutation index; see Sect. 3.16. The entry in the corresponding row and column of the table provides the local index of the node within the n-face from the perspective of the target cell. These lookup tables are filled within the fill_own_dofs_permutations deferred binding of reference_fe_t. We note that this latter binding, and permute_dof_lid_n_face, are declared as overridable bindings in Listing 6 on purpose. This lets, e.g., subclasses of reference_fe_t to be used in conjunction with (*properly oriented*; see Sect. 3.16) n-simplex meshes to implement the former such that the own_dof_permutations(:) member variable is not allocated nor filled, and the latter such that always returns the identity transformation.

### Creating reference_fe_t Polymorphic Instances

Central to any OO software system relying on abstract data types is the approach chosen to create polymorphic instances at runtime. For simplicity, FEMPAR follows the so-called simple factory design pattern [85]. It takes the form of a single stand-alone function, called make_reference_fe, which selects the dynamic type of the polymorphic instance to be returned at runtime based on the values of its dummy arguments topology and fe_type. (For example, assuming the topology of an hexahedron and fe_type_lagrangian, then it will select its dynamic type to be hex_lagrangian_reference_fe_t, i.e., the concrete data type implementing Lagrangian-type FE spaces on top of n-cubes.) Before returning, it calls a deferred binding of reference_fe_t, called create, which is responsible to leave the reference_fe_t in a fully functional state. The interface of this deferred binding is shown in Listing 8.

**Figure:**
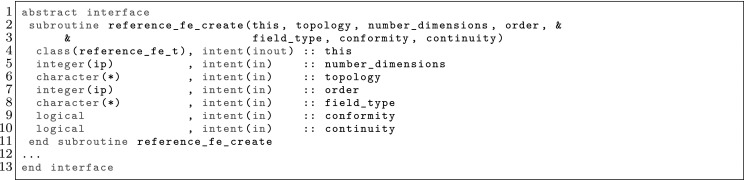
Listing 8. The signature of the create binding of reference_fe_t.

We remark that field_type is only a free parameter for Lagrangian FEs (i.e., for a particular reference_fe_t subclass). In other words, it must be field_type_vector for Raviart-Thomas and edge elements. We note that despite its fix set of dummy arguments interface, it has been proven to be sufficient to fully describe all subclasses currently available in FEMPAR; see Sect. 6.5. However, in the event that it is needed, and with extensibility in mind, a single parameter dictionary of ${<}key, value{>}$ pairs might have been used instead; FEMPAR indeed relies on an implementation of this data type where *key* is a string (typically denoting the name of the parameter), and *value* a scalar or arbitrary rank array of intrinsic or even user-defined types.^13^

### Enumeration of reference_fe_t Subclasses

There is a rather complex data type hierarchy rooted at reference_fe_t in FEMPAR, which has been judiciously designed with code re-use as the main driver. (For example, Lagrangian FE spaces on top of n-cubes and n-simplices share member variables and code that can be gathered into a common base data type.) For the sake of brevity, in this work we do not cover in full detail the implementation of the data types in this hierarchy (except those details given in Sects. 5 and 9.5). However, for completeness, it is convenient to enumerate those reference_fe_t subclasses that, at present, are available in this hierarchy. These subclasses, which lay at the leaves of the hierarchy, are the following ones:

- hex and tet_lagrangian_reference_fe_t. Space of polynomials of arbitrary degree *k* on top of n-cubes (i.e., tensor-product like spaces ${\mathcal {Q}}_{\varvec{k}}$) and n-simplices (i.e., ${\mathcal {P}}_{\varvec{k}}$), respectively, for the discretization of either scalar-valued, vector-valued or tensor-valued fields; see Sect. 3.8. By selecting the ownership relationship among node functionals and n-faces appropriately (see Sect. 6.3), this FE space can be either globally continuous, or entirely discontinuous across cell boundaries.
- hex and tet_raviart_thomas_reference_fe_t. The vector-valued Raviart-Thomas FE of arbitrary degree *k* on top of n-cubes, and n-simplices, resp., suitable for the mixed Laplacian problem and some fluid flow problems. Global FE functions of this space (in its conformal variant) have continuous normal components across cell faces; see Sect. 3.9 for details.
- hex and tet_nedelec_reference_fe_t. The vector-valued curl-conforming Nédélec FE of arbitrary degree *k* on top of n-cubes, and n-simplices, resp., suitable for electromagnetic problems. Global FE functions of this space (in its conformal variant) have continuous tangential components across cell faces; see Sect. 3.9 for details.
- void_reference_fe_t. A software artifact that represents a FE space with no DOFs at all, neither at the cell interiors, nor at their boundary n-faces. This sort of software resource has been proven extremely efficient for: (1) the numerical solution of a PDE on a subdomain of our original discretized domain (which thus has to be aligned with the cells boundaries); (2) the numerical solution of a PDE using XFEM-like discretization techniques (which are grounded on FE spaces that do not assign DOFs to cells exterior to the embedded domain); (3) to simplify the implementation of discretization methods for PDE problems that involve coupling at the interface level, e.g., fluid-structure interaction.

Apart from these reference_fe_t subclasses, there are already concluded developments within this hierarchy in a beta version of the code, such as B-splines [8], and other scheduled developments, such as div-conforming FEs [7].

## The Description of the Physical Domain: The triangulation_t Abstraction

A central abstraction in all FE numerical simulation codes is the one that describes the triangulation/mesh $\mathcal {T}_h$ of the physical domain $\Omega \subset \mathbb {R}^d$ in which our problem is posed. (In practice, the mesh generation for $\Omega$ introduces a geometrical error, and the mesh is in fact over an approximated domain $\Omega _h$). In FEMPAR, this abstraction is called triangulation_t. With flexibility, and code reuse in mind, this is an abstract data type. In Sect. 7.1, we introduce triangulation_t, and the mechanism that it provides to its subclasses in order to preserve encapsulation and data hiding, while still letting subclasses to store and access to data efficiently. For completeness, in Sect. 7.2, we introduce details underlying the implementation of a particular concrete subclass of triangulation_t.

### An Abstract Triangulation Representation and Its Software Implementation

In this section, we present an abstract (conceptual) representation of a triangulation that FEMPAR exposes to user-level applications and other library software abstractions that are grounded on it (see, e.g., Sect. 10). This conceptual representation is provided by a set of abstract derived data types (and the methods bounded to them) to which we have converged as a result of our experience in accommodating a wide range of state-of-the-art FE discretizations and solver techniques within a single framework, from desktops/laptops, to high-end distributed-memory supercomputers (see Sect. 2).

For the sake of brevity, in this work we restrict ourselves to a subset of this representation that only provides support to the implementation of high-order conforming and non-conforming FE discretizations grounded on *conforming meshes* in a serial computing environment. We stress, however, that the actual (complete) representation also incorporates concepts to express the mesh in a distributed-memory environment (e.g., the set of cells of a subdomain is divided into local cells and a layer of cells owned by remote subdomains, which we denote as *ghost cells*). It also provides support to the implementation of high-order *hp*-adaptive (i.e., on locally refined, non-conforming meshes) conforming and non-conforming FEs (using hanging node constraints [82] and subface integration over a facet between cells of different refinement level, respectively) and to the implementation of XFEM-type techniques (see [60] and references therein); provided an implicit representation of the geometry of the domain, a background mesh is able to know whether a cell is interior, exterior or cut by the domain, and in the latter case, to provide the coordinates of the intersection points. This extra expressivity comes in the form of additional data types and an extended set of methods for those data types that are covered in this section. We stress, however, that neither the former nor the latter ones will be covered in this section.

Although our abstract representation of a triangulation has been proven to have high expressivity, we do not claim, however, that our triangulation representation is universally applicable to the implementation of arbitrary numerical discretization and solver techniques. It indeed has been designed such that extra extensions are foreseen to satisfy further requirements.

The triangulation representation encompasses both topological and geometric data. A triangulation is conceived as a partition of $\Omega$ into a set of cells (*d*-faces). Each cell is uniquely identified by a global identifier in the range $\texttt {cell\_gid}=1,\ldots ,\texttt {num\_cells}$.^14^ Apart from the cells, a triangulation is also composed by a set of lower dimensional objects, i.e., a set of *k*-faces, for $k=0,\ldots ,d-1$. We will also refer to elements in this set as “vefs”, provided that in the $d=3$ case, it is composed of vertices, edges, and faces. Each of the objects in this set is uniquely identified by a global identifier in the range $\texttt {vef\_gid}=1,\ldots ,\texttt {num\_vefs}$.^15^

Apart from the cells and vefs, a triangulation also encompasses adjacency data. This sort of data describes how n-faces in a mesh are related to each other. We denote by *F* the set of all n-faces in the mesh, by $F^k$ the set of all *k*-faces, and by $F_i$ and $F^k_i$ the *i*-th n-face (of arbitrary dimension) and the *i*-th *k*-face (of fixed dimension *k*), respectively. In conforming meshes, there are mainly two relevant types of adjacency relationships, namely *composition* (*m*-faces that are part of a *k*-face for $m<k$) and *neighbourhood* (*m*-faces around a given *k*-face for $m>k$). Following [87], the set of *m*-faces adjacent to $F^k_i$, is denoted by $F^k_i \langle F^m \rangle$ (i.e., the operator $\langle \cdot \rangle$ selects from the set the *m*-faces adjacent to the one in the left). A triangulation conforming with FEMPAR abstract representation should be able to provide the composition data $F^3_i \langle F\rangle$, and the neighbourship data $F_i \langle F^3 \rangle$, that is, n-faces that compose each cell and cells around n-faces.

A triangulation also includes geometry data. Cell geometries are represented by a map $\varvec{\Phi }_K$ of a polytope ${\hat{K}}$ in the reference space to the physical space (see Sect. 3). This map is represented as a function of a *scalar* FE space (e.g., grounded on high-order Lagrangian FEs or B-splines), with its DOF values being the vectors of node coordinates (i.e., point_t instances) in the physical space.

At the core of the software design in charge of providing the triangulation-related data covered so far is an abstract data type named triangulation_t. (The rationale behind this data type being abstract will be made clear in the course of this section.) This data type is defined as shown in Listing 9. triangulation_t is conceived as a template to which all subclasses have to conform. On the one hand, it is composed by a (*minimal*) set of member variables encompassing data common to any triangulation. In particular, any triangulation is embedded in a num_dimensions-dimensional space, and is composed of a total number of num_cells (num_dimensions-dimensional) cells and num_vefs vefs, respectively; see Lines 3–5 of Listing 9, respectively. On the other hand, triangulation_t is equipped with a set of *deferred* methods that the subclasses of triangulation_t must implement; see Lines 11–18. The rationale underlying these methods requires further elaboration, to be discussed in the sequel.

**Figure:**
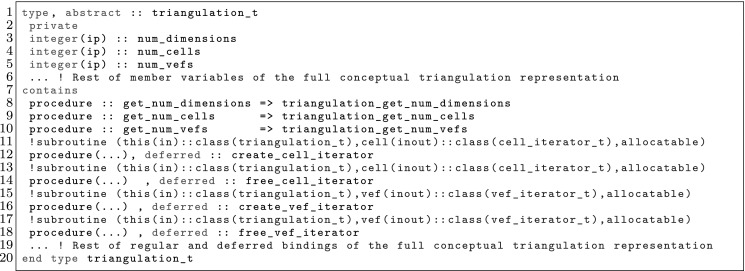
Listing 9. The triangulation_t abstract data type.

In order to construct a conceptual view of triangulation_t suitable for the user (and library) code needs, FEMPAR relies on the so-called *iterator* OO design pattern [88]. Iterators are data types that provide sequential traversals over the *full sets of objects* that all together (conceptually) comprise triangulation_t as a mesh-like container. There are several different iterators available, each one related to a different set of objects to be traversed. For example, cell_iterator_t provides traversals over the set composed of all cells, while vef_iterator_t over the one composed of all vefs.^16^ In our software design, iterators are created and freed by a set of public TBPs provided by triangulation_t; see Lines 11–18 of Listing 9. Thus, for example, the expression call triangulation%create_cell_iterator(cell) creates an iterator on the cell client-space instance, while call triangulation%free_cell_iterator(cell) frees it. Iterators sequentially traverse objects in increasing order by their global identifiers. However, we note that triangulation_t subclasses are completely free to decide how to internally label these objects.^17^

As the reader might have already noted from the minimal set of member variables in Listing 9 (among others), our software design is such that we want to provide *complete flexibility* to concrete subclasses of triangulation_t with respect to how do they internally layout the (topology and geometry) data to be provided. To this end, triangulation_t is an abstract class that defers this decision to its subclasses. There is a clear separation among how the data is handled (i.e., stored and accessed) by the *private data structures* (member variables) underlying triangulation_t subclasses, and the conceptual/abstract view of triangulation_t exposed to FEMPAR users. This view renders triangulation_t easily accessible and understandable. Whereas the public interface of triangulation_t being used by client codes is designed to be stable over time, the internals of triangulation_t subclasses, however, are allowed to (and are subject to) change over time (e.g., in order to accommodate further optimizations, additional requirements, etc.). At the price of dynamic run-time polymorphism, triangulation_t subclasses might be designed such that they strongly strive to preserve encapsulation and data hiding while *still storing and accessing to data efficiently*. Thus, e.g., a triangulation_t subclass in charge of handling structured/uniform meshes of simple domains may decide to not explicitly store the cell-wise global vef identifiers, nor the vertex coordinates of the mesh, but instead to provide them implicitly on demand as a function of the global cell identifier.

Apart from encompassing the logic underlying the actual traversal over objects of the set at hand, iterators also have the following crucial responsibility. Following the software concept of “accessors” presented in [17], they are able to tease out the data related to the current object on which they are seated from the global arrays and rest of private data structures that comprise the internals of the corresponding triangulation_t subclass. They therefore do not explicitly store, e.g., the global vef identifiers of the current cell. Instead, they know how to fetch them from the corresponding triangulation_t subclass into data structures suitable for the user needs. Provided that it is the responsibility of triangulation_t subclasses to decide how to internally layout data, iterators are abstract data types as well, and most of its TBPs are deferred/virtual. This also justifies why the methods in the Lines 11–18 of Listing 9 are deferred, and why the corresponding iterator dummy arguments, polymorphic allocatable. It is ultimately the responsibility of the concrete subclass of triangulation_t to decide on execution time the dynamic type of the polymorphic variable being created.

Let us next discuss the rationale underlying the design of iterators over cells and vefs. These data types are defined in Listing 10, where set must be actually replaced by the corresponding name uniquely identifying the set of objects to be traversed by the iterator at hand, i.e., either cell or vef. In Fig. 7, we illustrate the implementation of a partial (selected) subset of the bindings of these data types.

**Figure:**
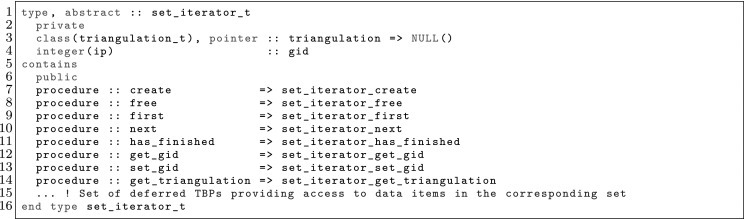
Listing 10. triangulation_t “set” (either cell or vef) iterators.

The create binding of set_iterator_t takes as input a polymorphic triangulation_t instance to be traversed, and leaves the iterator positioned in the first object of the set, i.e., in a state ready to start the sequential traversal over all of its objects; see Fig. 7. This method (like free) is not intended to be directly called by the user. Instead, triangulation_t clients should rely on the deferred bindings of triangulation_t presented in Listing 9. The init, next, and has_finished bindings let clients to position the iterator on the first object of the set, move to its next object, and check whether all of its objects have been already traversed or not, respectively; see Fig. 7.

**Fig. 7:**
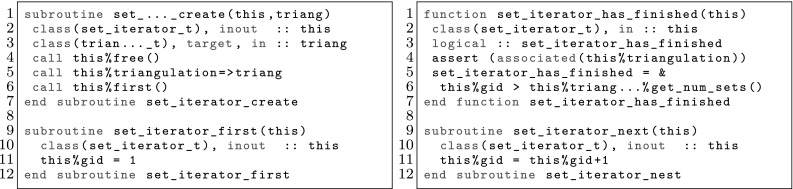
Implementation of a partial (selected) subset of the bindings of set_iterator (see Listing 10)

The actual set of (deferred) TBPs of a triangulation_t iterator highly depends on the type of object being pointed. We now briefly discuss those TBPs in the set corresponding to cell and vef iterators that provide support to the subset of the triangulation conceptual representation we are focusing on. These are in particular enumerated in Listing 11.

**Figure:**
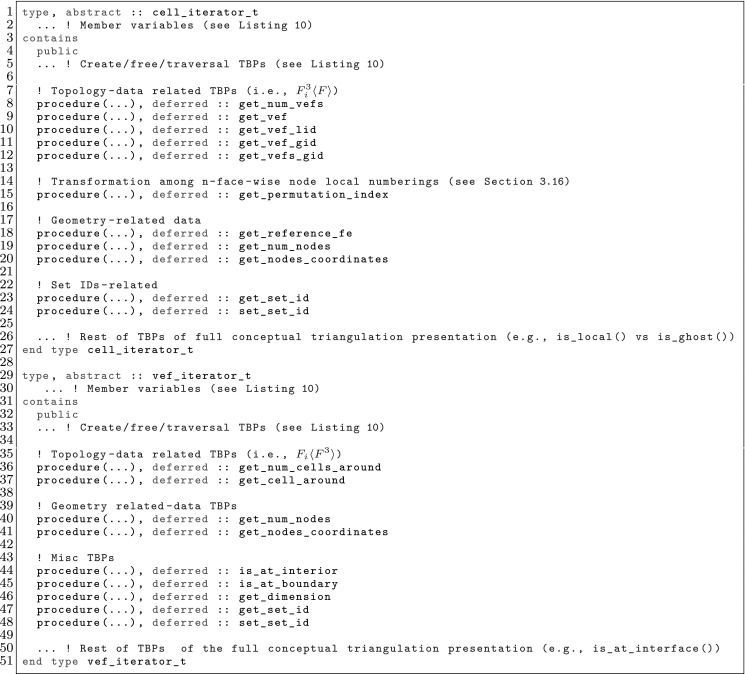
Listing 11. A subset of the deferred TBPs of the cell_iterator_t and vef_iterator_t data types (follow-up to Listing 10).

The TBPs in Lines 8–12 of Listing 11 are in charge of providing data related to the composition relationship $F^3_i \langle F\rangle$. In particular, the get_num_vefs binding returns the number of vefs on the boundary of the mesh (i.e., the cardinality of the composition relationship). Given the local index of a vef in a cell (within the range $1,\ldots ,\texttt {num\_vefs}$), get_vef positions the vef_iterator_t instance on input such that it points to this vef, while get_vef_gid, returns its global identifier; get_vef_lid performs the inverse translation to the one of get_vef_gid. Finally, get_vefs_gid let the client obtain the global identifier of all vefs of the current cell in one shot provided a user-space pointer to integer array. The semantics of this last TBP are such that subclasses of cell_iterator_t are not allowed to allocate the provided pointer, but to associate it with existing (internal) memory (for increased performance and memory leaks avoidance).

The TBP in Line 15 of Listing 11 provides support to the implementation of the transformation procedure described in Sect. 3.16. In particular, this binding has to be invoked on a cell_iterator_t instance positioned in the source cell, and given a cell_iterator_t positioned on the target cell, and the n-face local identifier within the former and latter cells, returns the permutation index; see Sect. 3.16. We stress that both the rotation and orientation indices can be always computed using the TBPs in the previous paragraph. For example, in order to determine the rotation index, one can extract the global id of the anchor vertex of the n-face in the target cell (by calling get_vef_gid), and then searching for this global id in the set of vertices that comprise the n-face in the target cell (using an iterator over the corresponding sublist in vertices_n_face; see Sect. 6.1). However, we preferred to provide a specialized deferred binding for such purpose in order to leave room for optimizations in triangulation_t subclasses. For example, in the case of a subclass that works with oriented meshes, then get_permutation_index may be implemented such that it always returns the permutation index corresponding to the identity transformation. In the case of a subclass of triangulation_t that is intended to remain static (or to be adapted very infrequently) during the course of the simulation process (see, e.g., Sect. 7.2), then it might be beneficial for performance to precalculate all possible permutation indices during set up into lookup tables, and re-use them all the way through without having to perform the aforementioned searches over and over again.

The TBPs in Lines 18–20 are in charge of providing the cell geometry related-data. In particular, get_reference_fe returns a polymorphic pointer to the reference_fe_t instance that describes the space of functions to which the mapping $\varvec{\Phi }_K$ belongs. get_num_nodes and get_nodes_coordinates return the number of nodes describing the geometry of the cell, and its associated coordinates in physical space, respectively. Instead of a pointer to an user-space array to be associated with internal storage (as get_vef_gids), get_nodes_coordinates takes a user-space (pre-allocated) array of type point_t instances, and fills it (because of reasons made clear in Sect. 8.3). Assuming that reference_fe_t is a bi-linear Lagrangian FE on a quadrilateral, then get_num_nodes would return 4 (one node per cell-vertex), while get_nodes_coordinates the coordinates in physical space of its vertices.

Any triangulation_t subclass should let its clients to classify the cells into sets. Each set is globally identified by an integer number, named set_id. The methods get_set_id and set_set_id let the caller to associate a set to the current cell, or to retrieve the set to which the cell is currently associated. Cells set identifiers are primarily (although not only) used by fe_space_t during its set-up; see Sect. 10. In particular, they instruct the latter to determine which reference_fe_t instances to use on top of the cells belonging to the same set. For example, assuming that we want to solve a scalar, single-field PDE problem on a subdomain of our original domain (that we assume to be aligned with the cells boundaries), we would use two different sets. The first for the cells that are interior to the subdomain, and the second for those that are exterior. Then we could associate e.g., a linear Lagrangian reference FE to cells in the first set, and void_reference_fe_t on those cells of the second set; see Sect. 6.5.

Sitting on a given vef, the TBPs in Lines 36–37 are in charge of providing data related to the adjacency relationship $F_i \langle F^3\rangle$. In particular, get_num_cells_around returns its cardinality, while get_cell_around returns a cell in this set. To be more precise, the latter TBP positions the instance of cell_iterator_t on input such that it points to a cell in this set identified with an index within the range $1,\ldots ,\texttt {get\_num\_cells\_around()}$. The order in which the cells around a vef are listed can be arbitrary, so that codes relying on triangulation_t should not assume, e.g., that they are ordered increasingly by their global cell identifiers. On the other hand, get_num_nodes and get_nodes_coordinates return the number of points on top of the vef (including those on top of the lower-dimensional ones on its boundary), and its associated coordinates in physical space, respectively; see Lines 40–41. We adopt the convention that these nodes are (locally) labeled (within the input/output array of point coordinates to be filled) according to the reference coordinate system of the *first cell* around the vef, i.e., the cell obtained as vef%get_cell_around(1,cell).

The TBPs in Lines 44–48 let the client to determine whether the vef is at the interior of the domain or on its boundary, the vef dimension (e.g., in 3D, it would return 0, 1, and 2 for vertices, edges, and faces, respectively) and to retrieve the set to which the vef is currently associated, or associate a new set to it, respectively. Sets in the case of vefs are primarily used to codify the boundary conditions of the PDE problem at hand, as discussed in Sect. 10.4.

At this point we are already in position to show user-level code that exploits the software design covered so far. In particular, Listing 12 splits the whole set of triangulation cells into two disjoint sets, those that are in contact to the boundary of the domain, and those that are in its interior.

**Figure:**
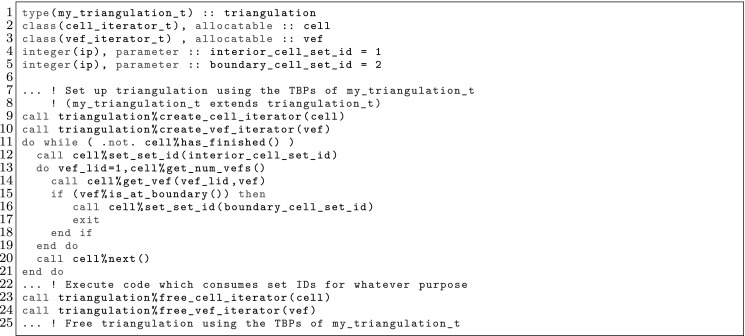
Listing 12. User-level code illustrating the usage of the data types and its associated TBPs supporting FEMPAR conceptual triangulation representation.

### An Example triangulation_t Subclass and Rationale

In this section, we discuss how a particular subclass of triangulation_t is internally organized in order to efficiently provide triangulation-related data by means of the software abstractions presented in Sect. 7.1. This subclass is static_triangulation_t. A static_triangulation_t codifies a *conforming mesh*, which is set up from scratch at the beginning of the simulation, and remains unaltered during the whole process. On the other hand, static_cell_iterator_t and static_vef_iterator_t are two non-abstract data type extensions of cell_iterator_t and vef_iterator_t, respectively. By overriding the set of deferred methods of the former ones, the latter ones tease out the data related to the current object on which they are seated from the global arrays and rest of private data structures that comprise the internals of static_triangulation_t.

There is no single approach to layout the data within a given triangulation subclass. The seek of an acceptable trade-off among memory consumption, computational time required to set up, update (if it applies), access to triangulation data, and the frequency on which these operations are performed should guide its internal organization. For example, in [87], two storage layouts are presented, and its memory and computational cost for the computation of any possible adjacency relationship is evaluated in 3D. The first one, called *one-level* representation, is defined by $F^1_i \langle F^0 \rangle$, $F^2_i \langle F^1 \rangle$, and $F^3_i \langle F^2 \rangle$, and by $F^0_i \langle F^1 \rangle$, $F^1_i \langle F^2 \rangle$, and $F^2_i \langle F^3 \rangle$ (neighbourhood information). In other words, it stores vertices of each edge, edges of each face, and faces of each cell, together with edges around vertices, faces around edges, and cells around faces. The second one, called *circular* representation, is defined by the composition information $F^1_i \langle F^0 \rangle$, $F^2_i \langle F^1 \rangle$, $F^3_i \langle F^2 \rangle$ (as above), together with the neighbourhood information $F^0_i \langle F^3 \rangle$ (cells around vertices). An important property of these two storage layouts is their *completeness*, i.e., the possibility to determine any adjacency without a loop over the entire mesh. The storage requirements for a uniform mesh of a cube domain with $N_c$ cells are $48 N_c$ (for hexahedra) and $24 N_c$ (for tetrahedra) in the former, and $32 N_c$ (for hexahedra) and $16 N_c$ (for tetrahedra) in the latter. However, the operation count for determining some adjacencies, although independent of $N_c$, is high. For example, in the case of the one-level representation, to obtain the cells around a vertex requires 48 (for hexahedra) and 140 (for tetrahedra) operations, whereas only one operation is needed to obtain cells around facets. In the case of the circular representation, these queries involve one and 148 (for hexahedra) or 299 (for tetrahedra) operations, respectively [87]. (We recall that both kind of adjacencies are required by FEMPAR as presented in Sect. 7.1.)

Another quite different storage data layout is the one followed by the triangulation in the deal.II library [17], essentially defined by the composition data $F^1_i \langle F^0 \rangle$, $F^2_i \langle F^1 \rangle$, and $F^3_i \langle F^2 \rangle$ (referred as hierarchical cell representation by the authors of the library), and the neighbourhood data $F^3_i \langle F^2 \rangle$ *stored cell-wise* (i.e., a given cell stores the identifiers of its cell neighbours across each face within the cell). Besides, the (potentially non-conforming) triangulation in this library is conceived (and explicitly represented) as a collection of trees, where the cells of a coarsest conforming mesh (generated by deal.II itself for simple domains, or read from a file from several file formats) form the roots, and the children branch off their parent cells, thus forming binary-trees, quad-trees and oct-trees in $d=1,2,$ and 3 spatial dimensions, respectively [17]. While both the ancestors (i.e., the so-called “inactive” cells) and leaf cells of the tree (i.e., the so-called “active” cells) are stored, only the latter ones actually form the partition of the domain. Apart from a hierarchy of cells, the deal.II triangulation also maintains a hierarchy of *k*-faces for $k=1,\ldots ,d-1$. Such quite complex data structure is justified by the authors for two reasons. First, it allows for an efficient implementation of adaptive mesh adaptation (including coarsening and refinement). The hierarchy of n-faces aids in the process of handling the so-called hanging node constraints required to build conforming FE spaces on top of non-conforming meshes. The second reason is the implementation of (geometric) multigrid preconditioners grounded on the adaptivity tree. In particular, such preconditioners require that DOFs are also associated to inactive cells. Thus, also inactive n-faces have to explicitly exist in the triangulation. In any case, such structure is hard to generate and maintain, and does not fit well when integrated with parallel octree libraries [89], like p4est [90]. The whole hierarchy must be generated from scratch on each mesh adaptivity step. However, based on our own experience, *such hierarchy is not really needed for an efficient implementation of adaptive refinement*. The second reason, i.e., the implementation of a serial hierarchical multigrid solver in deal.II, would probably be more complicated without such a hierarchical representation of the mesh.

While the hierarchical cell representation in deal.II has been proven to be successful in the implementation of highly complex *hp*-adaptive FE discretization [82] and reduces memory consumption over $F^3_i \langle F\rangle$, the restriction of the global vef identifiers to a cell (a very frequent operation in FE codes), becomes significantly more expensive in this storage layout as this operation requires permutations among the reference coordinate system of the cell that owns the vef to the one to which we are restricting to; the same applies to the restriction of global DOF identifiers to a cell when the DOFs are stored n-face-wise. Furthermore, it is a *non-complete* storage layout. In particular, neighbourship data $F_i \langle F^3 \rangle$ has to be computed by the user by means of a loop over all cells. Besides, it prevents library support to loops over the facets of the mesh, and access to the neighbouring cells, a natural operation in the implementation of DG methods. In our experience, facet-loop based integration of DG terms (versus cell-loop based) leads to a software that is significantly easier to use, as it might be designed such that most of the complexity underlying facet integration can be hidden to the user (see Sect. 9). Finally, although it is very efficient for hierarchical and local mesh adaptation (within each subdomain), the most severe drawback is its costly set up (from scratch) for a given initial conforming coarse mesh (this can be mitigated by reducing the coarse mesh resolution, at the price of potentially losing geometry modelling accuracy), and, in a distributed-memory environment, the even more costly regeneration of an adapted non-conforming forest of trees after a re-distribution step among processes for dynamic load-balancing [90]. Indeed, in  [89], the latter is reported as the second more costly operation in the simulation pipeline, only below the linear solver step.

The static_triangulation_t data type *explicitly* stores the composition data $F^3_i \langle F\rangle$, and the neighbourship data $F_i \langle F^3 \rangle$ within its internal (private) member variables.^18^ The memory consumption of such *complete* storage layout is $52 N_c$ (hexahedra) and $28 N_c$ (tetrahedra), which is less than twice the one of the one-sided and circular representations [87]. At the price of this increased memory consumption, static_triangulation_t is able to provide the required adjacency data with $\mathcal {O}(1)$ arithmetic complexity. Besides, the cell-based storage of the composition relationship is perfectly suited for its migration in parallel distributed-memory environments. On the other hand, the amount of *permanent storage* of this data layout can be reduced if one exploits the fact that neighbourship data is only required in very specific parts of the code. For example, unstructured mesh generators usually provide only the composition data $F^3_i \langle F^0 \rangle$. In such a case, static_triangulation_t requires the neighbourship data $F^0_i \langle F^3 \rangle$ (plus the reference cell topology data encompassed within the reference_fe_t instance mapped to each cell; see Sect. 6.1) in order to set up the composition data $F^3_i \langle F^1 \rangle$ and $F^3_i \langle F^2 \rangle$. It is also needed in triangulation_t subclasses suitable for distributed-memory computers, among others, to set up the data structures required to perform nearest neighbour exchanges of DOFs nodal values among subdomains. (We stress that this process requires to globally identify interface DOFs consistently among subdomains sharing such DOFs .) In this latter scenario, this adjacency data is only required for n-faces that lay on the inter-subdomain interface (and not for those on the interior). The evaluation of facet integrals (as designed in FEMPAR, see Sect. 9) also requires at least $F^2_i \langle F^3 \rangle$ and $F^1_i \langle F^2 \rangle$, in 2D and 3D, respectively. The use of the full adjacency data can be needed for the implementation of advanced numerical discretization schemes, e.g., for the implementation of nodal-based *shock detectors* for monotonic FEs [58, 59]. Due to the aforementioned reasons, we decided to design static_triangulation_t such that it permanently stores such data, but we stress that our software design is such that a triangulation subclass is always free to offer methods that set up and destroy these data on demand to reduce the amount of permanent data storage.

The static_triangulation_t data type, together with a selected set of its bindings, is defined as shown in Listing 13. Before going into more detail, there are two main points to remark with respect to how this type internally layouts its data. First, it relies all the way through on intrinsic Fortran allocatable arrays. These sort of data structures are perfectly suited for the particular case of static_triangulation_t, due to its static nature. We stress, however, that more efficient data structures (i.e., able to mitigate the effect of frequent/costly allocatable array re-allocations) would be convenient if it also had to support mesh adaptation (e.g., a linked list, or even better for data locality, a data structure with semantics close to std:vector of the C++ standard template library, which in fact is already in FEMPAR but not included for brevity). Second, for increased data locality during cell and vef sequential traversals (and thus a more efficient on the memory hierarchy of modern computer architectures) the data is not stored into cell-wise or vef-wise local arrays, but into global arrays that are indexed either by the global cell or vef identifiers.

**Figure:**
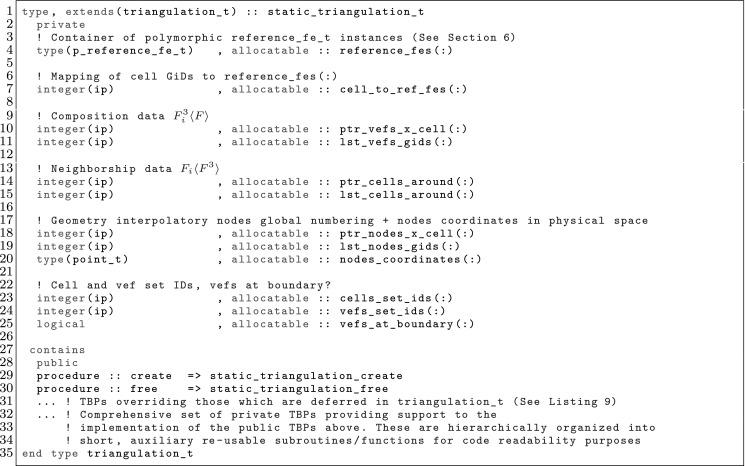
Listing 13. The internals of static_triangulation_t and a selected set of its bindings.

A collection of reference_fe_t polymorphic instances is stored in the reference_fes(:) array (see Line 4 of Listing 13). These instances are uniquely identified (within the local scope of static_triangulation_t) by their position in this array. For a given cell with global identifier cell_gid, the FE space of functions to which the cell mapping $\varvec{\Phi }_K$ belongs, is described by the reference_fe_t instance with identifier cell_to_ref_fes(cell_gid) in the collection; see Line 7. The member variables used to store the composition data $F^3_i \langle F\rangle$ are encompassed within Lines 10–11 of Listing 13. As stated above, the global vef identifiers are stored cell-wise, in the lst_vefs_gids(:) array, which is in turn (indirectly) addressed by the ptr_vefs_x_cell(:) array. In particular, the ones assigned to the vefs on cell cell_gid start and end in position ptr_vefs_x_cell(cell_id) and ptr_vefs_x_cell(cell_id+1)-1 of lst_vefs_gids(:), respectively. Thus, e.g., the implementation of the (overridden) get_num_vefs TBP in static_cell_accessor (see Listing 12), just determines the number of vefs on the boundary of the current cell as ptr_vefs_x_cell(cell_id+1)-ptr_vefs_x_cell(cell_id). On the other hand, the member variables used to store the adjacency data $F_i \langle F^3\rangle$ are encompassed within Lines 14–15 of Listing 13. The global identifiers of the cells around a vef vef_gid start and end in position ptr_cells_around(vef_gid) and ptr_cells_around(vef_gid+1)-1 of lst_cells_around(:), respectively.

The geometry-related data is handled by the member variables in Lines 18–20. In particular, during the set up of static_triangulation_t a global numbering of the nodes of the global FE space describing the geometry of the mesh is internally built. (The process that generates such numbering is identical to the one described in Sect. 10.3, so that we omit it here to keep the presentation short.) In particular, the global node identifiers restricted to cell cell_gid start and end in position ptr_nodes_gids(cell_id) and ptr_nodes_gids(cell_id+1)-1 of lst_nodes_gids(:), respectively. These global node identifiers are used to (indirectly) address the global array of nodes coordinates in Line 20. The cells_set_ids(:) and vefs_set_ids(:) arrays are used to store the user-provided cell and vef set identifiers (see Sect. 7.1), respectively, while vefs_at_boundary(:), whether the corresponding vef lays on the boundary of the domain or not.

Finally, the static_triangulation_create binding sets up a new static_triangulation_t instance. There are two options for creating a static_triangulation_t in FEMPAR, depending on whether the mesh is structured or unstructured. In the first case, FEMPAR provides the machinery for the automatic generation of a triangulation on simple domains (e.g., a unit cube), currently of brick (quadrilateral or hexahedral) cells. This function is implemented exploiting a tensor product structure of the space, numbering cells and vefs using lexicographical order. The second way to create a static_triangulation_t instance is from a mesh data file, e.g., using the GiD mesh generator [91].

## Evaluation of Cell Integrals

In this section, we describe the data structures required to perform the numerical integration of the local matrices. In order to compute cell integrals (12), one needs (among others) functionality to evaluate the shape functions and their derivatives at the quadrature points in the physical cell and the determinant of the Jacobian at the quadrature points in the reference cell. In turn, the evaluation of the shape functions and derivatives in the physical cell rely on their evaluation (and possibly the evaluation of the Jacobian) in the reference cell (see, e.g., (13) and (14)). We note that the evaluation of ${\hat{\Psi }}$ does not require any additional information; it is the identity for Lagrangian elements and only requires the Jacobian in the reference cell for vector-valued shape functions (see (17) and (18)). In the following, we present a set of data types that contain all this information.

The evaluation of cell integrals involves the data type quadrature_t that represents the quadrature $\mathrm{Q}$, interpolation_t, that stores the values of the shape functions and its first derivatives (either in the reference or physical space) at the quadrature points of $\mathrm{Q}$, and a cell_map_t that describes the mapping from a reference to a physical cell $\varvec{\Phi }_K$ (e.g., Jacobian-related data). Additionally, the data type cell_integrator_t provides the machinery to compute the interpolation_t corresponding to the physical space from the one at the reference space and the cell_map_t at every cell of the triangulation. In the following sections, we cover in detail these software abstractions.

### Numerical Quadrature

The data type that in FEMPAR represents an arbitrary quadrature rule is called quadrature_t and is defined as shown in Listing 14.

**Figure:**
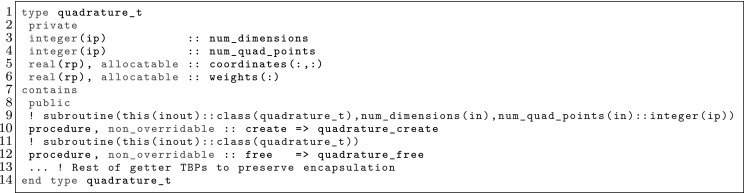
Listing 14. The quadrature_t data type.

In Listing 14, coordinates(:,gp) and weights(gp) store, respectively, $\hat{\varvec{x}}_{gp} \in {\mathbb {R}}^{\mathtt{num\_dims}}$ and $\mathrm {w}_{gp}$, for $gp=1,\ldots ,\mathtt{num\_quadrature\_points}$. It might readily be observed from the interface of its create binding that quadrature_t is designed to be simply a placeholder for the quadrature points coordinates and its associated weights. Indeed, this binding essentially allocates coordinates(:,:) and weights(:). The code that ultimately decides how to distribute the quadrature points over ${\hat{K}}$ and set up its associated weights is actually bounded to the reference_fe_t implementors through the deferred binding with interface shown in Listing 15.

**Figure:**
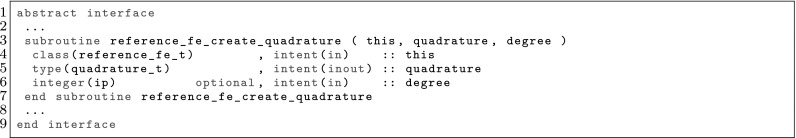
Listing 15. The interface of the create_quadrature deferred binding of reference_fe_t.

All reference_fe_t subclasses currently available in FEMPAR select by default a Gaussian quadrature that exactly integrates mass matrix terms (within their implementation of the binding in Listing 15) by invoking fill_*_gauss_legendre methods at lines Lines 13 and 14 in Listing 14. This quadrature can be solely determined from the attributes of the reference_fe_t implementor at hand (its topology and order).^19^ However, in other more demanding situations, e.g., the integration of a trilinear weak form, the user can provide the desired quadrature degree through the degree optional dummy argument. If more general scenarios to the ones currently covered (e.g., a non-Gaussian quadrature) are to be addressed, then the interface might be modified such that an optional parameter dictionary is passed instead.

### Evaluation of Reference Cell Shape Functions

As commented in the introduction of this section, to compute cell integrals (12), one needs to evaluate shape functions and their derivatives in the physical cell, which in turn rely on their evaluation in the reference cell (see, e.g., (13) and (14)). The values of the shape functions and their first derivatives at a set of quadrature points provided by a quadrature_t instance are stored in the interpolation_t data type presented below. The same data type can be used to store this data in the reference or physical space.

Let us start with the evaluation of shape function in the reference space. The local FE space on top of ${\hat{K}}$ actually depends on the particular reference_fe_t implementor at hand. Consequently, this functionality has to be offered through a deferred binding of this abstract type. The interface of this binding is declared in Listing 16. The subroutine overriding it in concrete subclasses is in charge of computing the shape functions values and derivatives at quadrature points in the reference space and stores them in a raw-data container of type interpolation_t (to be discussed later in this section).

**Figure:**
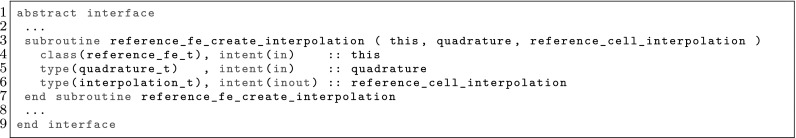
Listing 16. The interface of the create_interpolation deferred binding of reference_fe_t.

Let us remark several points related to this interface. First, this binding is typically called only once, and the data pre-computed and stored within the passed interpolation_t dummy argument is repeatedly re-used when transforming these values to an actual cell; see Sect. 8.4. Second, this binding is designed such that all functions are evaluated at all quadrature points within a single call, instead of following a (much) finer granularity approach in which only one function is evaluated at a quadrature point per call.^20^ Third, we stress that the actual implementation of this deferred binding in FEMPAR computes shape functions values and first derivatives in the reference space, whereas it lets the caller to selectively decide whether to compute or not the second derivatives of the shape functions, provided that they are expensive to compute and only required in very particular scenarios; see Sect. 3.7. Indeed, the code implementation of this feature is of cross-cutting nature, being reflected in several interfaces and data types in which the cell (and face) integration functionality is split. We will nevertheless omit here (and in the rest of sections) details regarding second derivatives (and its optional computation) in order to keep the presentation simple.

Let us now discuss on the rationale underlying interpolation_t. This data type is not exposed at all to the user of FEMPAR. It is instead used as an internal low-level container that lets the data types involved in the implementation of cell integrals exchange the sort of data subject to consideration. It is ultimately the responsibility of the concrete reference_fe_t subclass to decide how the data is actually laid out within the member variables of interpolation_t. Thus, reference_fe_t is the only data type that can access or modify interpolation_t. In its current flavour, interpolation_t is a concrete (i.e., non-abstract) data type with a fixed set of multi-rank allocatable array member variables for storing shape function values and derivatives. For example, the one storing shape function values is a 3-rank array, where a reference_fe_t implementor may choose its indices, from left to right, to refer to the component of the shape function, the shape function, and the quadrature point, respectively. The reference_fe_t subclass is, however, completely free to lay out the data in these arrays, and it is in this flexibility where the extensibility of the software design to accommodate several FE space realizations resides. This, indeed has been proven to be sufficient to (efficiently) implement all FE spaces currently available in FEMPAR, including scalar, vector, and tensor-valued Lagrangian FEs (where higher-rank spaces are determined as the tensor product of the scalar spaces, and shape functions have only one non-zero component), and genuinely vector-valued FE spaces (where more than one component of the shape function may be non-zero).

### Geometrical Mapping

A basic building block is the mapping $\varvec{\Phi }_K$ among the reference cell ${\hat{K}}$ coordinate system and the one corresponding to an actual cell *K* of the triangulation in the physical space; see Sects. 3.2 and 3.3. For example, we are able to pull back the gradients of the shape functions from the reference to the physical space in (14) using the Jacobian of the transformation evaluated at quadrature points, or to evaluate the source term at quadrature points in real space. The Jacobian is also required to the transform the integral from the physical to the reference space in (12) and to compute the Piola transformations in div and curl-conforming FE spaces (see (17) and (18)). The derived type cell_map_t in FEMPAR is designed to be a placeholder for the data required to provide this sort of services. It is declared as shown in Listing 17. The rationale underlying the inheritance relationship among cell_map_t and base_map_t will be made clear in Sect. 9.

**Figure:**
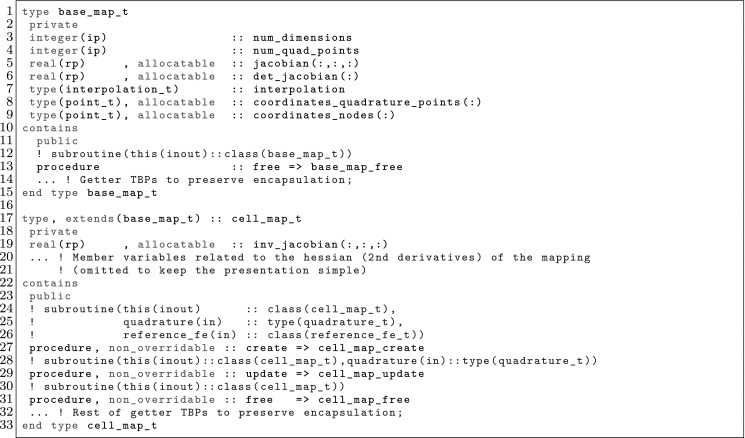
Listing 17. The cell_map_t data type.

The create binding of cell_map_t takes as input a quadrature_t instance with a set of integration points where $\varvec{J}_K(\hat{\varvec{x}}_{gp})$, $\varvec{J}_K^{-1}(\hat{\varvec{x}}_{gp})$, and $|\varvec{J}_K(\hat{\varvec{x}}_{gp})|$ are to be evaluated (see Listing 17). These geometry-related data are stored in the jacobian(:,:,gp), inv_jacobian(:,:,gp), and det_jacobian(gp) allocatable array member variables of cell_map_t, respectively, and allocated during a call to this binding. Apart from a quadrature_t instance, cell_map_t also requires a description of the (discrete) space of functions to which $\varvec{\Phi }_K$ belongs. FEMPAR supports mappings $\varvec{\Phi }_K$ belonging to abstract FE spaces (e.g., high-order polynomial FE spaces or spline-based spaces). The reference_fe dummy argument of polymorphic type reference_fe_t serves the purpose. (We note that dynamic run-time polymorphism in this particular context let us re-use cell_map_t, e.g., with an arbitrary cell topology.) It turns out that the only information that reference_fe_t has to provide to cell_map_t are its shape functions, first derivatives, and (on demand) second order derivatives at the quadrature points (in the reference space). The interpolation member variable (see Listing 17) is used by reference_fe to exchange this sort of data with cell_map_t via a call to the create_interpolation binding of the former (see Listing 16) during a call to the $\texttt {create}$ binding of the latter.

While the create TBP of cell_map_t is designed to be called once, the update TBP of cell_map_t is, however, designed to be called multiple times, once per every cell *K* of the triangulation. A pre-condition of update is that the nodes_coordinates(:) scratch member variable (see Listing 17) has been loaded with the coordinates in real space of the nodes describing the geometry of *K* (stored into point_t instances). Once this pre-condition is fulfilled, $\varvec{\Phi }_K$ can be expressed as a linear combination of the reference_fe_t shape functions with nodes_coordinates(:) being the corresponding coefficients in the expansion. At this stage, coordinates_quadrature_points(:), which stores the coordinates of quadrature points in real space, and jacobian(:,:,:), can be easily computed. Finally, inv_jacobian(:,:,:) and det_jacobian(:) can be computed from jacobian(:,:,:) using straightforward numerical algorithms.

### Evaluation of Shape Functions in the Physical Space

The user code that evaluates cell integrals in (12), may need the value, gradient, curl, and divergence of the shape functions at the integration points in the physical space, provided that we want to unburden FEMPAR users from the complexity of having to explicitly apply mapping transformations. As commented in Sect. 3, the mapping that transforms a shape function $\hat{\phi }^{a}(\hat{\varvec{x}})$ in the reference FE space into the one in the physical space $\phi ^{a}(\varvec{x}) = {\hat{\Psi }}_K(\hat{\phi }^{a}) \circ \varvec{\Phi }^{-1}_K$, depends on the particular FE space at hand; see Sects. 3.8, 3.9, and 3.10 for details. For this reason, the actual code that performs these transformations is not actually bounded to cell_map_t, but to reference_fe_t, through the deferred binding with interface declared in Listing 18.

**Figure:**
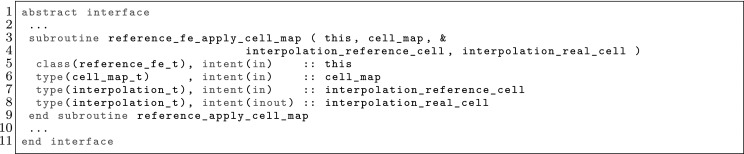
Listing 18. The interface of the apply_cell_map deferred binding of reference_fe_t.

The interpolation_reference_cell input dummy argument of apply_cell_map (see Listing 18) must have been obtained from a call to the binding in Listing 16 invoked on the same reference_fe_t instance. The output dummy argument interpolation_real_cell holds the shape functions and their derivatives evaluated at quadrature points in physical space (see (13) and (14)). It is also assumed that, on input, interpolation_real_cell already contains the data that does not have to be re-computed on each mesh cell, e.g., the value of the shape functions on integration points for Lagrangian FEs; see the discussion related to the update binding below for the strategy that we follow in order to fulfill this requirement. This leaves room for optimization in the implementation of this deferred binding (on subclasses), since these quantities do not have to be re-computed on each cell. The reference_fe_t subclass uses the cell_map_t instance (passed to the apply_cell_map binding, see Listing 18) as a placeholder for the data required to provide the mapping transformations required.

We stress, however, that interpolation_t is a low level structure that is not designed as a data type that FEMPAR users have to interact with, for reasons made clear in Sect. 8.2. Therefore, we need to introduce an additional data type in our software design, called cell_integrator_t, that, among other services, is able to fetch raw data from interpolation_t into field data types (i.e., scalars, vectors, and tensors) the user can be easily familiarized with. This data type is declared as shown in Listing 19.

**Figure:**
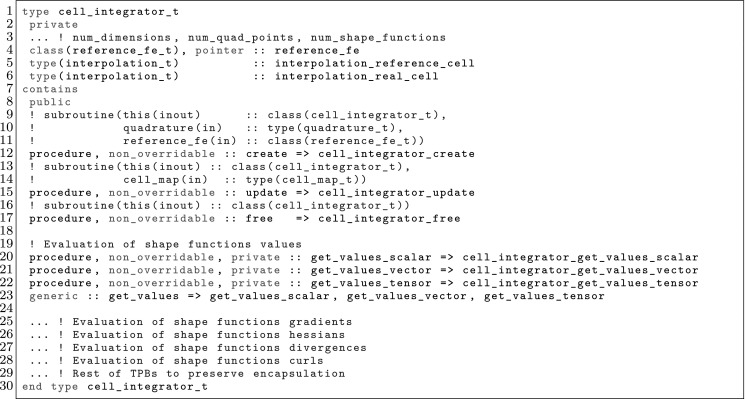
Listing 19. The cell_integrator_t data type.

An instance of cell_integrator_t is created from a quadrature rule (where the shape functions and their derivatives are to be evaluated) and a polymorphic reference_fe_t instance describing the reference FE space at hand; see interface of the create binding in Listing 19. During this stage, reference_fe creates the interpolation_reference_cell member variable of cell_integrator_t via create_interpolation; see Listing 16. It also clones interpolation_reference_cell into interpolation_real_cell, and copies the contents of the former into the latter. This lets cell_integrator_t to fulfill later on the pre-condition on the last dummy argument of apply_cell_map. The create binding also associates its polymorphic pointer reference_fe member variable to the reference_fe_t instance provided to it on input. This pointer is required later on by the update and get_* bindings (see discussion in the sequel).

The update binding of cell_integrator_t simply invokes apply_cell_map on its polymorphic reference_fe member variable, using the instance of cell_map_t provided on input to update, and the two interpolation_t member variables as actual arguments, respectively; see Listings 18 and 19. It leaves the cell_integrator_t instance on which it is invoked in a state such that it is able to provide the services it was primarily designed for. These are offered through the get_values, get_gradients, get_divergences, get_curls, etc., *generic bindings*. We note that cell_integrator_t is designed such that it can handle either scalar, vector, or tensor-valued reference_fe_t instances (see Sect. 6.2). With this purpose in mind, each of the aforementioned generic bindings are overloaded with subroutines that have appropriate interfaces for these three types of FEs. For example, the subroutine overloading get_gradients in the case of scalar-valued FEs is declared and implemented as shown in Listing 20, with vector_field_t representing a *d*-dimensional rank-1 tensor; the interface of the one corresponding to vector-valued FEs only differs from the one above on the base type of the gradients allocatable array dummy argument, which is of base type tensor_field_t (i.e., data type representing a *d*-dimensional rank-2 tensor).

**Figure:**
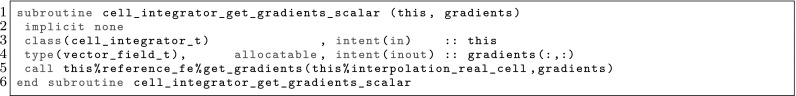
Listing 20. The code implementing the get_gradients_scalar binding of cell_integrator_t ultimately relies on a deferred binding of reference_fe_t with the same name.

Let us remark some important points with respect to the subroutines overloading the generic bindings of cell_integrator_t. First, we note that the actual argument passed in place of, e.g., the gradients(:,:) dummy argument in Listing 20, is intended to be actually declared in code written by the user of FEMPAR. Provided that FEMPAR can support variable degree FEs on top of different triangulation cells (see Sect. 10), the allocatable attribute of the gradients(:,:) dummy argument not only unburdens the user from the complexity of having to (pre)allocate this array, but even from the one associated to variable degree FEs. For example, if on input, the size of gradients(:,:) is not sufficient to hold the data to be provided by the cell_integrator_t instance corresponding to the reference_fe_t on top of the current triangulation cell, then it can be re-allocated to the appropriate size. Second, this binding is designed such that all functions are evaluated at all quadrature points within a single call, justifying why the dummy argument has to be a rank-2 allocatable array.^21^ At this point, let us note that all subroutines subject to consideration ultimately rely on (deferred bindings of) reference_fe_t; see, e.g., line 5 in Listing 20. We recall that reference_fe_t must mediate in any process that requires retrieving data from interpolation_t; see Sect. 8.2.

### Cell Integration User Code Example

At this point of the discussion, we are already in position to show user code that evaluates the entries of the (current cell) local matrix for the Example 3.1 presented in Sect. 3.1. This code is sketched in Listing 21. This code would be bounded to a subclass of the discrete_integration_t abstract data type presented in Sect. 11.2 suitable for the Galerkin discretization of the Poisson problem.

**Figure:**
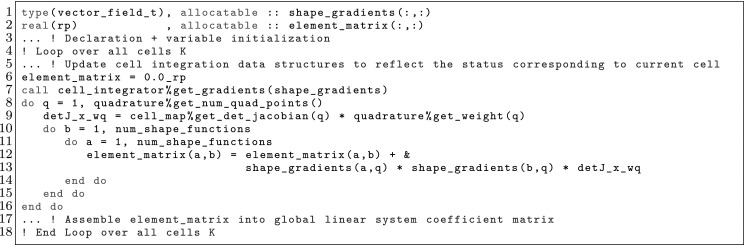
Listing 21. User-level code illustrating the usage of cell integration data structures in order to compute the element matrix for the Example 3.1 presented in Sect. 3.1.

The reader may note from Listing 21 that FEMPAR also offers an expression syntax that lets its users code weak forms in a way that resembles their mathematical expression. The user is in charge of explicitly writing the expression of the numerical integration in the reference cell, i.e., of explicitly implementing the quadrature point summation (loop) and handling the determinant of the Jacobian and the quadrature point weighting in (12). However, the evaluation of the shape function and their gradients, curls, etc., at the quadrature points in the physical space (e.g., expressions (13) and (14)) are completely hidden to the user. This can be achieved using a feature of modern programming languages called *operator overloading*. (We refer to [67] for a detailed exposition of this mechanism in Fortran2003.) Common (contraction) operations among tensors are provided by means of overloaded intrinsic and library-defined operators. For example, the operator(*) generic interface (corresponding to the * intrinsic operator) has to be overloaded with the single contraction of rank-1 tensors, and the multiplication of a rank-1 tensor by a scalar to let our code compile. A crucial design requirement in the seek of code efficiency is that no dynamic memory allocation/deallocation is involved as the partial evaluation of sub-expressions proceeds (in the order dictated by operator associativity and priority rules in Fortran). In order to fulfill this requirement, the data types representing vectors and tensors are declared such that their entries are stored in an array member variable *of size known at compilation time*. This size is stored in the library-level parameter constant SPACE_DIM, defined as the maximum number of space dimensions of the physical space in which the physical problem is posed. By default, FEMPAR is prepared to deal with 3D simulations, but the code is written such that a 2D simulation might also be performed if SPACE_DIM is equal to 3, at the price of extra storage and computation.^22^ Higher dimensional problems could be considered by compiling FEMPAR with a larger value for SPACE_DIM. Apart from avoiding dynamic memory allocation/deallocation during the evaluation of weak forms, this solution has the following advantages: (1) there is no need to explicitly have the number of dimensions as a member variable of the data types representing vectors and tensors; (2) the limits of the loops implementing tensor contraction operations are known at compilation time, enabling compiler optimizations. We finally stress that we preferred this solution over the usage of Fortran2003 parameterized data types [67] due to the lack of support of this feature in some of the most popular compilers widely available on high-end computing environments.

## Evaluation of Facet Integrals

This section covers the data types (and their interactions) in which the evaluation of integrals over the facets of the triangulation is grounded on. The integration of facet-wise matrices and vectors (see, e.g., (23)) involves the evaluation of shape functions and gradients of the neighbouring cells at the quadrature points within the facet in the physical space and the Jacobian of the facet map at the reference space. As described in Sect. 8, the former quantities are computed at every neighbouring cell from their values at the reference space and the Jacobian of the cell mapping. The evaluation of interior facet also requires the computation of the permutation $\Pi (\mathrm{gp})$ (see (25)) provided that the coordinate systems of the cells surrounding the facet might not be aligned in physical space.

In FEMPAR the assembly process of the global linear system underlying the discrete weak problem (20) involves two loops, over all cells and facets, respectively. In the former loop, a cell-wise matrix $\mathbf {A}^{K}$ and vector $\mathbf {f}^{K}$ are computed per each cell. These hold the partial contributions of the cell to the corresponding entries of the global coefficient matrix and right-hand side vector, respectively. The data structures involved in their efficient computation have been already covered in Sect. 8. In the latter loop, and assuming that we are sitting on an interior facet $F\in \mathcal {F}^{\Omega }_{h}$, four facet-wise matrices, namely $\mathbf {A}^{F}_{K^+ K^+}$, $\mathbf {A}^{F}_{K^+ K^-}$, $\mathbf {A}^{F}_{K^- K^+}$, and $\mathbf {A}^{F}_{K^- K^-}$ are computed (see Sect. 3.12).

**Fig. 8:**
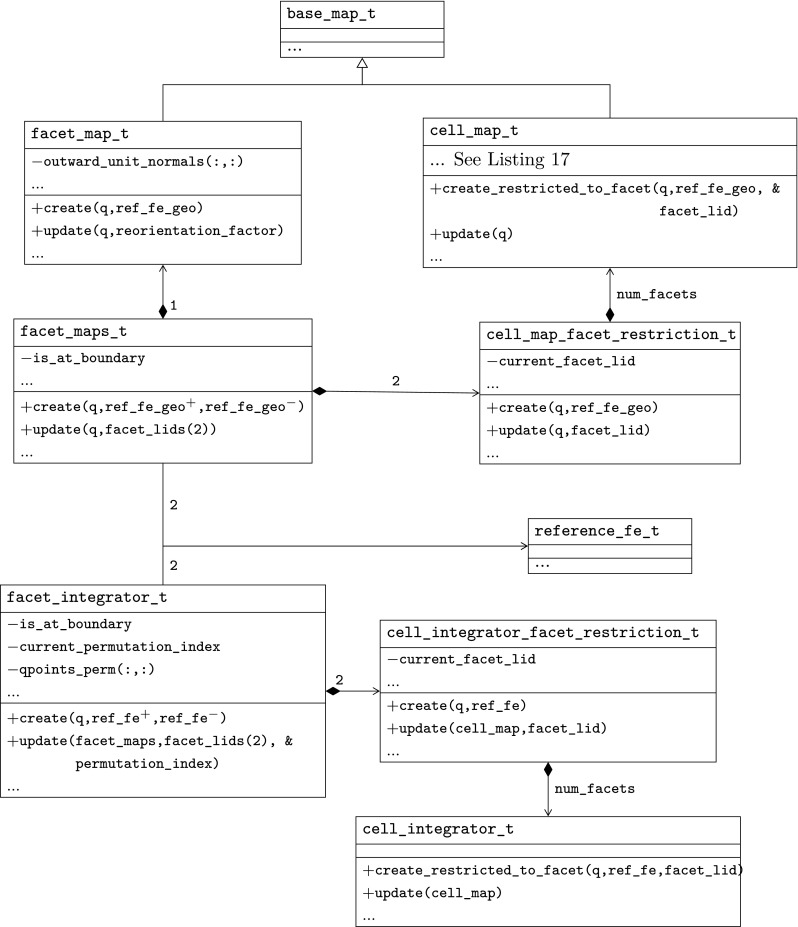
UML class diagram of the data types on which the numerical evaluation of facet integrals is grounded on

We depict in Fig. 8 a complete UML class diagram of the data types involved in the evaluation of facet integrals and their relationships. The data types the user has to ultimately interact with are $\texttt {quadrature\_t}$, which holds the facet quadrature points and weights, $\texttt {facet\_maps\_t}$, which handles (i.e., stores, updates, provides) all the geometrical related data of the facet and neighbouring cells $K^+$ and $K^-$, and, finally, $\texttt {facet\_integrator\_t}$, which stores and updates shape function values and first derivatives, and provides shape function values, gradients, curls, etc., of $K^+$ and $K^-$ evaluated at facet quadrature points in real space. The rest of data types in Fig. 8 are auxiliary data types, not exposed to the user, which aid the latter two in the implementation of their corresponding services. The reader might readily observe in Fig. 8 that our software design is such that the data types that provide support to the evaluation of cell integrals, i.e., quadrature_t, cell_map_t, and cell_integrator_t (see Sect. 8), can be re-used to a large extent for the evaluation of facet integrals. As we will see in the rest of the section, some of the methods to be invoked in order to control their respective life cycles in the context of facet integrals are nevertheless different from the ones to be invoked in the context of cell integrals; see, e.g., the signature of the create_restricted_to_facet binding of cell_integrator_t in Fig. 8 compared to that of its create binding in Listing 19.

### Numerical Quadrature

The data type quadrature_t is designed to be a placeholder for the *facet quadrature* points $\hat{\varvec{x}}_q$ and its associated weights $\mathrm{w}_{q}$. However, the code that ultimately decides how to distribute $\hat{\varvec{x}}_q$ over the reference facet ${\hat{F}}$ coordinate system, and set up $\mathrm{w}_{q}$, is bounded to reference_fe_t, in particular through the deferred binding with interface shown in Listing 22. We refer to Sect. 8.1 for the rationale underlying the degree optional dummy argument of this deferred binding.

**Figure:**
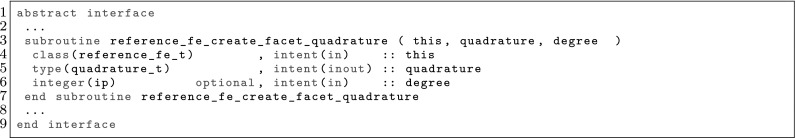
Listing 22. The interface of the create_facet_quadrature deferred binding of reference_fe_t.

### Geometrical Mappings

The facet_maps_t data type in Fig. 8 handles the geometrical facet mapping and the two geometrical cell mappings. The facet mapping is represented by facet_map_t, whereas the cell mappings by cell_map_t; see Sects. 9.2.1 and 9.2.2, respectively.

#### Facet Mapping

As illustrated in Fig. 8, facet_maps_t is composed, among others, of a single instance of type facet_map_t. The member variables (and associated code) that are common to facet_map_t and cell_map_t are factored into a superclass base_map_t (see Listing 17). facet_map_t handles all data related to the facet map $\varvec{\Phi }_F$, including the facet outward unit normals (see Fig. 8). An extra 2-rank real allocatable array member variable, outward_unit_normals(:,:), stores the facet outward unit normals (with respect to $K^+$ by convention) evaluated at facet quadrature points in real space, as required by (25); $\varvec{n}^-(\varvec{x}_\mathrm{gp})$ can be simply obtained as $\varvec{n}^-(\varvec{x}_{\mathrm{gp}})=-\varvec{n}^+(\varvec{x}_\mathrm{gp})$.

Let us now see how facet_maps_t controls the life cycle of its facet_map_t instance. The create binding of facet_map_t takes a quadrature_t instance with the facet quadrature points. $\varvec{J}_F(\hat{\varvec{x}}_\mathrm{gp})$ and $|\varvec{J}_F(\hat{\varvec{x}}_\mathrm{gp})|$ are evaluated at these quadrature points and stored in the jacobian and det_jacobian member variables, which are allocated during a call to this binding together with outward_unit_normals(:,:). Apart from a quadrature_t instance, facet_map_t also requires a description of the discrete, lower dimensional space of functions on top of the reference facet ${\hat{F}}$ to which $\varvec{\Phi }_F$ belongs. The ref_fe_geo dummy argument of create, of polymorphic type reference_fe_t, is provided for this purpose; in particular, facet_maps_t sends the reference_fe_t on top of $K^+$ as an actual argument to the ref_fe_geo dummy argument in order to comply with the above described convention for the normals. The interpolation_t member variable of facet_map_t (see Listing 17) is used by ref_fe_geo to exchange with facet_map_t the shape function values and their derivatives. To this end, reference_fe_t is equipped with the create_facet_interpolation deferred binding (see its signature in Listing 23) that computes these quantities on top of the reference facet ${\hat{F}}$.

**Figure:**
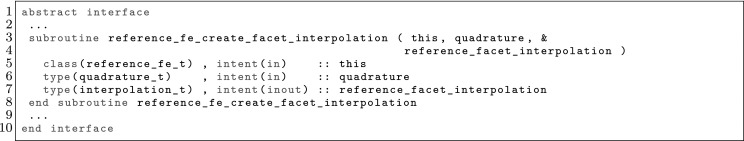
Listing 23. The signature of the create_facet_interpolation deferred binding of reference_fe_t.

The update binding of facet_map_t is intended to be called once per facet loop iteration, i.e., once per each facet of the triangulation. A pre-condition of this binding is that the nodes_coordinates(:) scratch member array of facet_map_t (see Listing 17) has been loaded with the coordinates in real space of the nodes that lay on the the facet.^23^ The update binding takes as input dummy arguments a quadrature_t instance and the real parameter reorientation_factor in order to adjust the sign of the facet normals (see (26)). Within update, quadrature_points_coordinates(:) and jacobian(:,:,:) can be easily computed from the basis shape functions and their first derivatives, respectively. On the other hand, det_jacobian(:) and outward_unit_normals(:,:) can be computed from jacobian(:,:,:). The former as stated in (24), while the latter as in (26).

#### Neighbouring Cells Mappings

The facet_maps_t data type is also composed by two instances of type cell_map_facet_restriction_t; see Fig. 8. These instances handle all data related to $\varvec{\Phi }_{K^\alpha }$, with $\alpha$ being either $+$ or −. Let us thus refer to these instances as cell_map_facet_restriction $^\alpha$, and to the polymorphic reference_fe_t instances on top of $K^\alpha$ as ref_fe_geo $^\alpha$. In turn, cell_map_facet_restriction $^\alpha$ are composed by as many cell_map_t instances as facets in $K^\alpha$. Provided that an actual facet $F$ can *potentially* have local identifier $F^\alpha$ in $K^\alpha$ within the range $F^\alpha =1,\ldots ,\text {num}\_\text {facets}(K^\alpha )$, having as many cell_map_t instances as facets per surrounding cell let us hold and (pre)calculate within these instances the result of evaluating the ${\hat{K}}^\alpha$ shape functions and their derivatives at the facet quadrature points for all facets in the reference system. To this end, the create binding of cell_map_facet_restriction $^\alpha$ is invoked (from the one corresponding to facet_maps_t) with the facet quadrature $\texttt {q}$ and ref_fe_geo $^\alpha$ as input actual arguments. It then walks over all possible local facet identifiers in the corresponding cell, and for each local facet identifier, invokes a specialized version of the create binding of the corresponding cell_map_t instance, named create_restricted_to_facet (that additionally requires the local facet identifier); see Fig. 8. The reference_fe_t is ultimately responsible to exchange this sort of data with cell_map_t. This service is in particular provided by the create_interpolation_restricted_to_facet deferred binding of reference_fe_t, with signature defined in Listing 24.

**Figure:**
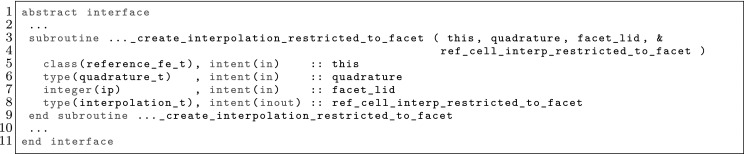
Listing 24. The signature of the create_interpolation_restricted_to_facet deferred binding of reference_fe_t.

As seen so far, the create binding of facet_maps_t is designed to be called right before the actual loop over all triangulation facets, and it sets up all the scratch data. It does so by covering all possible scenarios corresponding to potential values of local facet identifiers within the two surrounding cells (even if some of these scenarios are not actually exposed in the triangulation). The update binding of facet_maps_t, however, is intended to be called sitting on a particular facet $F$ of the triangulation, and it has to only update those two cell_map_t instances within cell_map_facet_restriction $^\alpha$ corresponding to the particular scenario at hand, i.e., to the particular combination of local facet identifiers $F^+$ and $F^-$ of the facet on which it is being updated. To this end, the update binding of facet_maps_t receives these local identifiers in facet_lids (see Fig. 8) and then calls the update binding of cell_map_facet_restriction $^+$ and cell_map_facet_restriction $^-$ with facet_lid=facet_lid(1) and facet_lid=facet_lid(2), respectively. The update binding of cell_map_facet_restriction_t picks up the cell_map_t corresponding to facet_lid and invokes the update binding of the latter. We stress that no specialized version of this binding is required in the context of facet integration, i.e., the same version discussed in Sect. 8.4 for cell integration can be re-used here.^24^ During the update process, cell_map_facet_restriction_t also registers in its current_facet_lid private member variable, the value supplied to the facet_lid dummy argument. This lets facet_maps_t to extract later on from cell_map_facet_restriction $^\alpha$ the *updated* cell_map_t instances; see discussion of facet_integrator_t in the sequel.

### Evaluation of Shape Functions in the Physical Space

The last data type that remains to be covered is facet_integrator_t; see Fig. 8. This data type is the counterpart of cell_integrator_t (see Sect. 8.4) for the case of facet integrals. In particular, it stores and updates shape function values and derivatives, and provides the values, gradients, curls, and divergences of the respective fields for both $K^+$ and $K^-$ evaluated at facet quadrature points in real space. As can be observed from Fig. 8, its overall design is very close to the one of facet_maps_t, with cell_integrator_facet_restriction_t and the cell_integrator_t instances it is composed of, playing the role of its counterparts in the scope of facet_maps_t (i.e., cell_map_facet_restriction_t and cell_map_t, respectively). There are, however, two major differences among these two. First, facet_integrator_t deals with (e.g., it is created from) the two polymorphic reference_fe_t instances (see ref_fe $^\alpha$ dummy arguments of its create binding in Fig. 8) on which the global FE spaces of functions $\mathcal {X}_h$, $\mathcal {Y}_h$ are grounded on. For example, the create binding of cell_integration_facet_restriction $^+$ invokes the create_restricted_to_facet binding of the cell_integrator_t for all facets $F^+$ within $K^+$. The latter computes at a given facet $\hat{\phi }^{a}_{K^+}(\hat{\varvec{x}}^{+}_\mathrm{gp})$, ${\varvec{\nabla }}\hat{\phi }^{a}_{K^+}(\hat{\varvec{x}}^{+}_\mathrm{gp})$ through the deferred binding create_interpolation...to_facet of reference_fe_t presented in Listing 24. Second, facet_integrator_t has to unburden the user from the complexity underlying the fact that the coordinate systems of $K^+$ and $K^-$ might not be aligned in real space. To this end, it is equipped with a private lookup permutation table, called qpoints_perm(:,:) in Fig. 8, that lets it translate facet quadrature points identifiers from the local numbering space of $K^+$ into the one of $K^-$. This table is allocated and filled during the create binding of facet_integrator_t, in particular by reference_fe_t through a deferred binding called fill_qpoints_permutations. Given the facet quadrature identifier $\texttt {gp}$ and the facet permutation index $\texttt {pi}$ (see Sect. 3.16), qpoints_perm(gp,pi) stores the value of $\Pi (\mathrm{gp})$ (see (25)). The permutation index is stored within the current_permutation_index of facet_integrator_t, extracted from the permutation_index dummy argument of the update binding. In turn, this parameter is extracted from the array facet_permutation_indices(:) of fe_space_t in Listing 27 (see Sect. 10). We note that for n-simplices, we consider a renumbering such that all facets have the same orientation on both cells that share it, as commented in Sect. 3.16. In this case, fill_qpoints_permutations fills the table with the identity permutation in all columns. We note that the re-orientation of the n-simplices can lead to mappings $\varvec{\Phi }_K$ such that $| \varvec{J}_K| < 0$, but this is not a problem as soon as one takes its absolute value, e.g., in (12).

### Facet Integration User Code Example

In order to grasp how the data structures covered so far are actually used together in practice, the Fortran pseudocode snippet at Listing 25 shows user’s space code in charge of evaluating the first integral in (22) for each interior facet in a loop over all facets. It would be bounded to a subclass of the discrete_integration_t abstract data type presented in Sect. 11.2 suitable for the non-conforming DG discretization of the Poisson problem.

**Figure:**
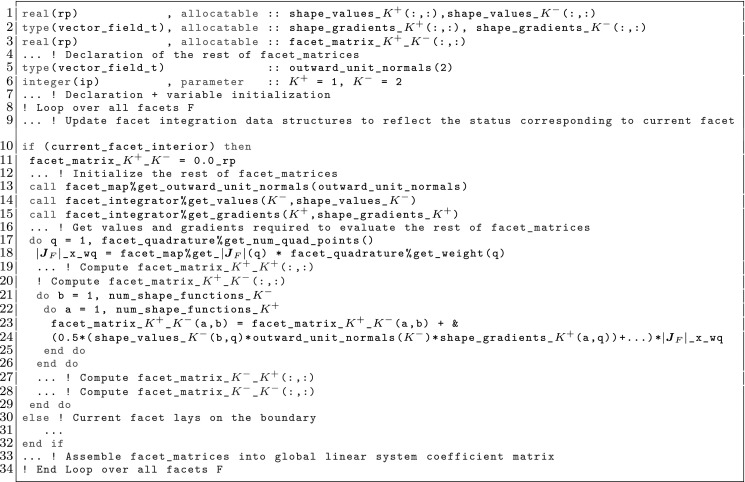
Listing 25. User-level pseudocode illustrating the usage of facet integration data structures in order to compute the first integral in (22) for each interior facet in a loop over all facets.

There are a pair of worth noting remarks about Listing 25. First, the call to the get_values() binding of facet_integrator_t in Line 14 already returns the permuted $K^-$ shape function values, i.e., shape_values_ $K^-$ (b,gp) actually stores $\phi ^{b}_{K^-}(\varvec{x}^{-}_{\Pi (\mathrm{gp})})$. Second, it is the so-called fe_space_t abstraction (to be covered in Sect. 10) the one in charge of creating the facet integration data structures on loop initialization and to update them at each facet loop iteration (see Line 9). Therefore, the user does not actually directly deals with all the data types bindings and their interactions illustrated in Fig. 8. In this example, it becomes evident that facet-loop based integration is very convenient for the implementation of DG methods, since it very much resembles the blackboard expressions (see, e.g., (20)).

### Change-of-Basis Implementation in a reference_fe_t Subclass

In this section, we provide a detailed presentation of how the change-of-basis required to compute the shape functions basis is implemented in a reference_fe_t subclass. In particular, we show the implementation for the Raviart-Thomas div-conforming FE on n-cubes in Sect. 3.5 (see also Sect. 3.9 for details). The pre-basis, e.g., ${\mathcal {Q}}_{(k+1,k,k)} \times {\mathcal {Q}}_{(k,k+1,k)} \times {\mathcal {Q}}_{(k,k,k+1)}$ in 3D, has to be generated before this subroutine is called; see, e.g., the evaluation of the pre-basis in Line 31 of Listing 26.

**Figure:**
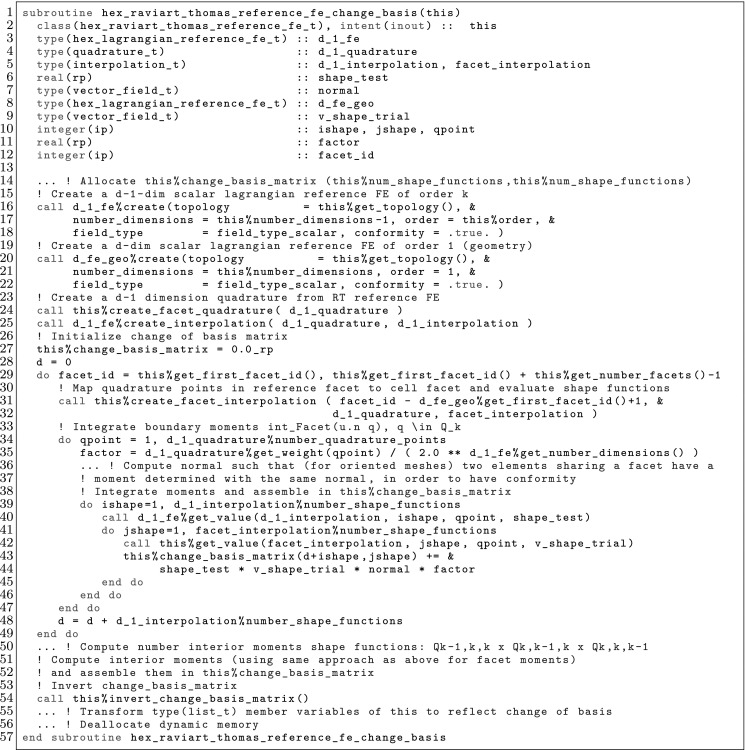
Listing 26. Implementation of the change-of-basis required for Raviart-Thomas divconforming FEs on n-cubes, following the procedure presented in Sect. 3.5.

We also present how to compute the boundary moments in (16) in Listing 26; interior moments are simpler and omitted for the sake of brevity. The implementation of the boundary moments requires: (1) to create the reference_fe_t that implements $[ {\mathcal {Q}}_{k \varvec{1}} ]^{d-1}$ in Line 16, (2) a facet quadrature on the reference facet in Line 24, and (3) the evaluation of the reference FE in the quadrature points in the interpolation_t in Line 25. We also require a Lagrangian (first order) FE that represents the geometry in Line 20. Next, we loop over all the facets of the cell and compute the values of the shape functions of the cell in the facet quadrature, stored in the interpolation_t instance in Line 31. With all these ingredients, we can compute the boundary moments for the pre-basis functions (see line 43) and assemble them in the change-of-basis matrix. After doing the same for interior moments, we just need to invert the change-of-basis matrix in Line 54. At this point, we have the shape functions basis as a linear combination of pre-basis functions. Thus, when one calls the fill_interpolation binding of the corresponding reference FE, it creates the pre-basis interpolation_t instance and next applies the change-of-basis matrix to compute the one for the shape functions basis, i.e., the placeholder where the evaluation of the shape functions and its derivatives (at the set of quadrature points for which the interpolation has been created) are stored. We note that the ownership of DOFs also changes in this process. The boundary moments (integrals of functions on facets) belong to the corresponding facet, whereas interior moments belong to the cell. Vertices and edges do not have DOFs in this case. The definition of the ownership is skipped for brevity.

## Integration and Global DOF Handling: The fe_space_t Abstraction

In this section, we introduce a software abstraction, referred to as fe_space_t, which represents (in the most general scenario) the mathematical concept of a global FE space $\mathcal {X}_h = \mathcal {X}^1_h \times \ldots \times \mathcal {X}^n_h$ obtained by means of the Cartesian product of global FE spaces $\mathcal {X}^{i}_h$ corresponding to each of the $i=1,\ldots ,n_\mathrm{field}$ field unknowns involved in a system of PDEs; see Sects. 3.6 and 3.11. Each $\mathcal {X}^{i}_h$ is described as a combination of: (1) an approximation $\Omega _h$ of the physical domain $\Omega$ provided by triangulation_t, i.e., a mesh-like container for the cells on which $\Omega _h$ is partitioned, their boundary lower-dimensional objects, and their adjacency relationships; see Sect. 7; (2) a description of the $n_\mathrm{field}$ reference FEs associated to each triangulation cell grounded on reference_fe_t; see Sect. 6.

These two basic building blocks equip fe_space_t with the tools required to provide the following two crucial services.^25^ On the one hand, it is in charge of handling (i.e., generating, storing, fetching) a *global* enumeration of the DOFs corresponding to each $\mathcal {X}^{i}_h$ taking into account the notion of conformity; see e.g., Sects. 3.6 and 6.2. On the other hand, it handles the data structures that are required to evaluate integrals over cells and facets (see Sects. 8 and 9, respectively). In particular, it judiciously sets up them, and orchestrates their respective life cycles and interactions, while unburdening the user (to a large extent) from the complexity (among others) inherent to high order FEs.

The OO design of fe_space_t (as the one of many other data types in FEMPAR, e.g., triangulation_t) strongly strives to preserve encapsulation and data hiding while still storing and accessing data efficiently (i.e., in a way that leverages data locality for the efficient exploitation of modern computer memory architectures). The user-friendly view of fe_space_t is implicitly (re)constructed by the data types (associated interfaces and interactions) that will be covered in Sect. 10.2. We now move on the approach that we follow for the internals of fe_space_t.

### The Internal Organization of fe_space_t

In this section, we *sketch* how the internals of fe_space_t are organized in order to efficiently deliver the two services outlined above. For simplicity, we restrict ourselves to a simplified version of fe_space_t that, to a large extent, captures the spirit of its actual counterpart in FEMPAR. The declaration of this simplified data type is shown in Listing 27.^26^

**Figure:**
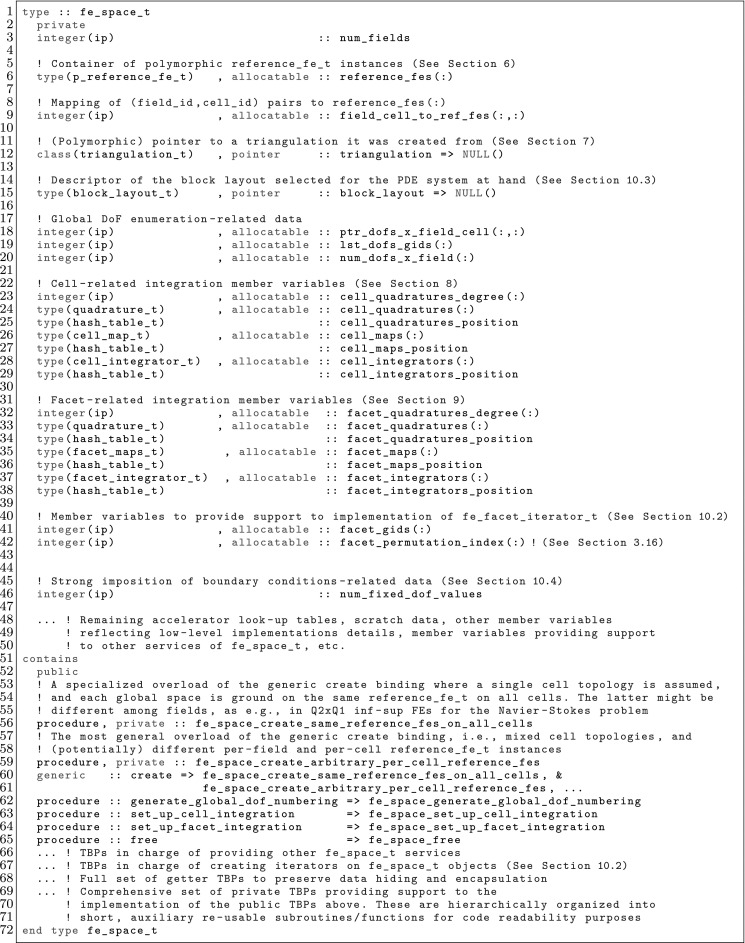
Listing 27. The internals of fe_space_t and a selected set of its bindings.

A collection of reference_fe_t polymorphic instances is stored in the reference_fes(:) array. These instances are uniquely identified (within the local scope of fe_space_t) by their position in this array. The global FE space corresponding to a given field, with identifier f_id in the range $1,\ldots ,{\texttt {num\_fields}}$ (with num_fields equal to $n_\mathrm{field}$ above), is described by: (1) the triangulation member variable (the rationale underlying it being polymorphic is made clear in Sect. 10.2; (2) its restriction to each cell provided by the reference FE space defined by the reference_fe_t instance with identifier field_cell_to_ref_fes(f_id,c_id) in the collection; c_id is assumed to be a positive integer in $1,\ldots ,\texttt {triangulation\%get\_num\_cells()}$ that uniquely identifies each cell.

The member variables used to handle the global DOF numbering are encompassed within Lines 18–27 of Listing 27. The global DOF identifiers are stored cell-wise, and field-wise within each cell, in the lst_dofs_gids(:) array, which is in turn (indirectly) addressed by the ptr_dofs_x_fe(:,:) array. In particular, the ones assigned to the local nodes related to field f_id on cell c_id start and end in position ptr_dofs_x_fe(f_id,c_id) and ptr_dofs_x_fe(f_id+1,c_id)-1 of lst_dofs_gids(:), respectively, if $\texttt {f\_id} < \texttt {num\_fields}$, and in position ptr_dofs_x_fe(f_id,c_id) and ptr_dofs_x_fe(1,c_id+1), respectively, if $\texttt {f\_id} = \texttt {num\_fields}$. The number of DOFs of the global FE space corresponding to each field (excluding those that are subject to strong boundary conditions) is stored in the num_dofs_x_field(:) array.

The member variable in Line 15 stores a reference to a data type that describes the block layout *currently selected* (i.e., it can be changed on demand) for the global matrix and right-hand side vector of the linear system (or a sequence of them) required for the solution of the PDE system at hand. The role of block_layout_t in the global DOF numbering generation process will be illustrated in Sect. 10.3.

The data structures that let fe_space_t handle the evaluation of cell integrals are declared in Lines 23–29 of Listing 27. The set_up_cell_integration binding sets up them. The method is intended to be called by the user’s program right before any cell integration loop. It ensures that any (scratch) data that can be computed on its final form in the reference cell is pre-computed *for any of the triangulation cells* while minimizing the number of integration data structures required for the particular scenario at hand. To this end, fe_space_t is equipped with three array containers of quadrature_t, cell_map_t and cell_integrator_t objects (see Lines 24, 26, and 28, respectively), which are indirectly addressed by the hash_table_t member variables with corresponding names.^27^ This is required because fe_space_t supports, e.g., non-conforming FE spaces with variable order per cell. A *unique identifier* (dynamically generated within the scope of fe_space_t) is assigned to each of the integration objects that must be created. The hash_table_t instances let fe_space_t transform these unique identifiers into container array positions from which the integration objects can be fetched.

The set_up_cell_integration method loops over all cells. Sitting on a cell, it determines an appropriate quadrature to be used on that cell and its associated unique identifier. (See discussion in the next paragraph for more details.) If this quadrature has not been generated yet (i.e., if the hash table lookup fails), then a new quadrature is created on the next free position of the cells_quadratures(:) array container, and a new identifier-position pair is inserted into the hash table. Otherwise, the quadrature is fetched from this array. The same process is repeated for the cell_map_t and cell_integrator_t instances. The former ones are uniquely determined by the combination of the unique identifier quadrature_t just created/fetched and that of the reference_fe_t instance on top of the current cell (see Sect. 7). On the other hand, a cell_integrator_t instance has to be associated to each field within the current cell; the cell_integrator_t instance corresponding to a field is uniquely determined by the unique identifier of the quadrature_t just created/fetched and the one of the reference_fe_t associated to that field (see Sect. 8.4). Therefore, the unique identifiers of the cell_map_t and cell_integrator_t instances required for the evaluation of cell integrals over the current cell can be easily determined combining the ones corresponding to the instances from which they are created. We recall that the unique identifier of the reference_fe_t instance on top of the current cell, c_id, for a given field, f_id, can be retrieved from reference_fe_id=field_cell_to_ref_fes(f_id,c_id), while the reference_fe_t instance itself from reference_fes(reference_fe_id).

The allocatable array member variable in line 23 (with as many entries as triangulation cells) can be used by the user in order to (optionally) determine the degree of the quadrature to be used on each triangulation cell. This member variable is allocated and initialized (during fe_space_t creation) to a reserved flag that instructs set_up_cell_integration to use an automatic (default) strategy to decide the degree of the quadrature to be used on each cell. This default strategy relies on a deferred binding of reference_fe_t, named get_default_quadrature_degree, which typically returns the quadrature degree for which mass matrix terms are integrated exactly (see Sect. 8.1).^28^ The strategy, in particular, walks over all reference_fe_t instances on top of the cell, and the one for which its (polynomial) reference cell functional space is of maximum order becomes ultimately responsible of creating the quadrature via an invocation to its create_quadrature deferred binding. Alternatively, the user may explicitly select the quadrature degree to be used on each cell. In such a case, create_quadrature is invoked to create a quadrature with the degree given by the corresponding entry in the cell_quadratures_degree(:); see Sect. 8.1. In any case (i.e., default or explicit quadrature degree), both the unique identifier of the reference_fe_t instance on top of the current cell and the quadrature degree are used to generate a unique identifier of the quadrature to be created/fetched.

On the other hand, Lines 32–38 of Listing 27 encompass those data structures required for the evaluation of (both boundary and interior) facet integrals; see Sect. 9. A very close rationale to the one underlying their cell counterparts is followed to set up these data structures. The set_up_facet_integration binding loops over all facets. Sitting on a facet, it determines an appropriate facet quadrature_t rule. The quadrature degree is either the default or a user-defined one (via the allocatable array member variable in Line 32). It also determines the unique identifier of the quadrature and of the rest of the facet-integration data structures, which are created as necessary, while handling their interactions. Both the topology of the two cells sharing the facet and the quadrature degree are used to generate a unique identifier of facet quadratures. The member variables in Lines 41–42 provide support to the implementation on the so-called fe_facet_iterator_t data type and will be covered in detail in Sect. 10.2. Finally, the member variable num_fixed_dofs in Listing 27 is used by fe_space_t to count how many DOFs are subject to strong boundary conditions; see Sect. 10.4.

### A Conceptual View of fe_space_t

Following the ideas presented in Sect. 7.1, fe_space_t offers a number of iterators to provide traversals over its objects, and uniform data access to its internals. Apart from iterators over cells and vefs, fe_space_t also provides traversals over facets by means of the so-called fe_facet_iterator_t data type. This iterator is essentially required to implement the evaluation of jump terms in, e.g., error estimators or DG methods in a user-friendly manner. For reasons made clear in the course of this section, a design goal to be fulfilled by fe_space_t iterators is that they are able to provide access to the same data as their counterpart triangulation_t iterators (see Sect. 7.1), and that they are able to do so *efficiently* while avoiding duplication of code bounded to the latter ones. For example, fe_cell_iterator_t should be designed such that it is also able to provide the coordinates (in physical space) of the nodes describing the geometry of the cell, apart from the global DOF identifiers on top of it.

Let us first discuss the design of iterators over cells and vefs (as the one of both follows the same lines). These data types are defined in Listing 28, where set must be actually replaced by either cell or vef.

**Figure:**
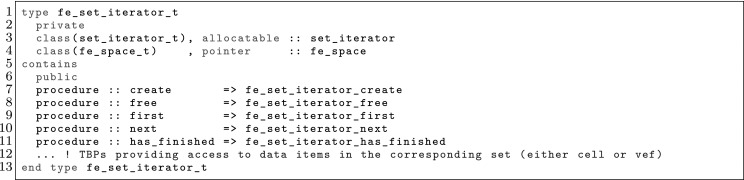
Listing 28. fe_space_t “set” (either cell or vef) iterators and the composition relationship with their counterpart triangulation_t iterators (set_iterator_t).

As shown in Listing 28, fe_set_iterator_t holds a *polymorphic* pointer to the fe_space_t instance to which it has to provide data access. Dynamic polymorphism is exploited here with extensibility and code reuse in mind. Any type extension of fe_space_t (e.g., the one suitable for distributed-memory environments), can also become the target of this polymorphic pointer, thus enabling reuse of data and code bounded to fe_set_iterator_t with these extensions. Of special relevance in Listing 28 is the composition relationship among the data type being defined and set_iterator_t, i.e., its triangulation_t iterator counterpart (see Sect. 7.1). This lets fe_set_iterator_t to fulfill the aforementioned design goal, i.e., to provide a superset of data over the class it is composed of, while still being able to access to any data stored within the triangulation scope. fe_set_iterator_t also reuses from set_iterator_t the code underlying the sequential traversal over all objects of the set. Indeed, as many other TBPs of fe_set_iterator_t, init, next, and has_finished TBPs of fe_set_iterator_t are simply implemented as wrappers of their counterparts in set_iterator_t. (We remark that this is possible provided that fe_space_t is deliberately set up such that it shares with triangulation_t a consistent global numbering for cells and lower-dimensional objects.)

At this point it is important to remark that the set_iterator_t instance that fe_set_iterator_t aggregates is also *polymorphic* (see Line 3 in Listing 28). As stated in Sect. 10.1 (in particular, see Line 12 of Listing 27), a fe_space_t instance is created from a polymorphic triangulation_t instance. The create binding of fe_set_iterator_t extracts the latter from fe_space_t, and then calls its create_cell_iterator binding (see Sect. 7.1), which becomes ultimately in charge of determining the dynamic type of the set_iterator_t member variable of fe_set_iterator_t (apart from leaving the iterator positioned in the first object of the set). This lets fe_space_t (and its associated iterators) to be re-used with any type extension of triangulation_t (e.g., the one suitable for distributed-memory computers and/or *h*-adaptivity). Likewise, the free binding of fe_set_iterator_t relies on the free_cell_iterator binding of triangulation_t in order to safely deallocate any dynamic memory allocation performed during creation. We stress that, as in the case of triangulation_t iterators, both the create and free TBPs are not intended to be directly called by the user. Instead, triangulation_t provides a set of (public) TBPs (as many as different iterators) for this purpose. For example, the expression call fe_space%create_fe_cell_iterator(fe_cell_iterator) creates an iterator on the *polymorphic* fe_cell_iterator client-space instance, while call fe_space%free_fe_cell_iterator(fe...) is in charge of safely deallocating this polymorphic instance.

The implementation of fe_facet_iterator_t is based on a very close rationale to the one of cell and vefs iterators, with subtle differences though; see Listing 29. Provided that fe_facet_iterator_t is a kind of fe_vef_iterator_t, it should provide the same set of data access methods of the latter (e.g., the cells sharing the facet). However, it should restrict the traversal to those vefs that are actually facets, and to be able to provide all data required for the implementation of jump terms over facets. As shown in Listing 29, fe_facet_iterator_t extends fe_vef_iterator_t. This automatically equips the former with the data access methods of the latter. On the other hand, it overrides those methods controlling the sequential traversals over the items in the set such that it restricts to facets, i.e., create/free/first/next/has_finished in Listing 29. The implementation of these methods relies on its member variable facet_gid, and the facet_gids(:) member variable of fe_space_t; see Line 41 of Listing 27. For a given facet with global identifier $\texttt {facet\_gid}$, facet_gids(facet_gid) holds the global vef identifier corresponding to the facet.

**Figure:**
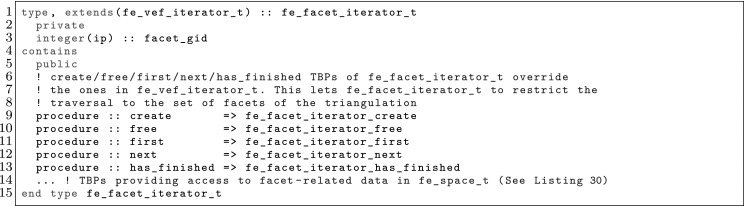
Listing 29. The fe_facet_iterator_t data type.

The actual set of TBPs of a fe_space_t iterator highly depends on the type of object being pointed to. For completeness, we now briefly discuss those TBPs in the set corresponding to cell and facet iterators, which provide support for the implementation of the two services of fe_space_t we are focusing on. These are in particular shown in Listing 30. This listing also includes the generic TBPs in Lines 35 and 68, although they will be discussed in Sect. 11.1.

**Figure:**
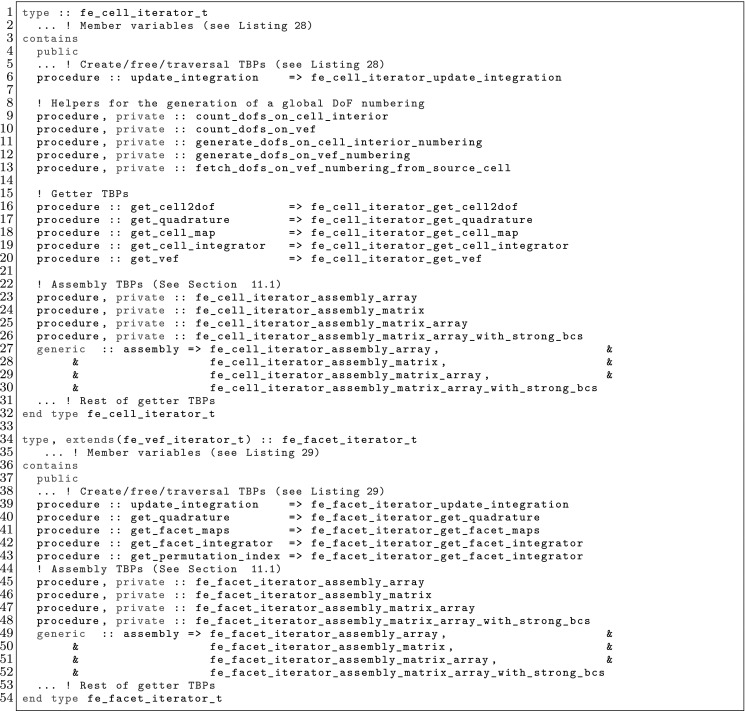
Listing 30. The fe_cell_iterator_t and its facet counterpart.

The TBPs in Lines 18–28, and 50–61 of Listing 30 let the user fetch from fe_space_t the integration data associated to the current cell and facet being pointed to, respectively. On the other hand, the update_integration bindings in Lines 6 and 47 perform those computations required to update these data structures such that they hold shape function values and derivatives evaluated at (current) cell and facet (quadrature points) in the physical space. The former binding is implemented as shown in Listing 31. Finally, the get_permutation_index TBP of fe_facet_iterator_t lets the caller to obtain the permutation index (see Sects. 3.16 and 9.3 for further details). The implementation of this method relies on the facet_permutation_indices(:) member variable of fe_space_t; see Line 42 of Listing 27. For a given facet with global identifier $\texttt {facet\_gid}$, facet_permutation_indices(facet_gid) holds the permutation index corresponding to the facet. We have decided to permanently store facet permutation indices for performance reasons. These can be reused over and over again (e.g., in a transient and/or nonlinear PDE problem) without the overhead associated to its computation on each traversal over the facets of the triangulation.

**Figure:**
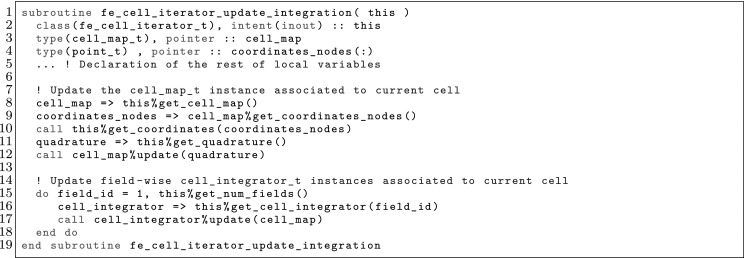
Listing 31. Implementation of the update_integration binding of fe_cell_iterator_t.

An update of the cell_map_t instance (associated to the cell pointed by the fe_cell_iterator_t instance on which this subroutine is invoked) is performed in Line 12 of Listing 31. It is followed by a loop over the number of fields of the PDE system at hand in order to update the cell_integrator_t for every field in Line 17. The update of the former requires that its nodes_coordinates(:) scratch member variable has been loaded with the coordinates in the physical space of the nodes describing the geometry of the cell at hand (see Sect. 8.3). This is in particular fulfilled in Line 10. The coordinates fetched by this call are actually stored within the triangulation. However, fe_cell_iterator_t can satisfy this query provided that it is composed of a cell_iterator_t instance; see Listing 28 and accompanying discussion. At this point, the reader should be already capable to grasp how the fe_facet_iterator_t counterpart of this subroutine is implemented, so that it is omitted here in order to keep the presentation short.

Going back to Listing 30, the binding in Line 16 lets the user fetch the field-wise global DOF identifiers that fe_space_t has associated to the node functionals on the current cell interior and its vefs. (The bindings in Lines 9–13 of Listing 30, however, assist fe_space_t on the generation of the global DOF numbering and their usage will be illustrated in Sect. 10.3.) This binding is implemented in Listing 32.

**Figure:**
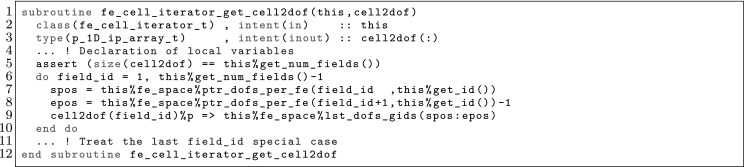
Listing 32. Implementation of the get_fe_dofs binding of fe_cell_iterator_t.

In Listing 32, p_1D_ip_array_t is assumed to be a data type with a single member variable, called p, declared as a pointer to a rank-1 integer(ip) array. For each field, the subroutine locates the region within the lst_dofs_gids(:) member variable corresponding to that field within the current cell, and then it associates to it the corresponding pointer in fe_dofs(:). At the expense of sacrificing type safety (in Fortran there is no mechanism to declare a pointer to be read-only), we avoid the costly re-allocation of user-level allocatable arrays that would be needed in the case of non-conforming FE spaces with highly varying degree polynomial spaces among cells.

To end up, the get_vef binding in Listing 30 sets up a fe_vef_iterator_t instance to point to the corresponding vef within the cell. As a consequence, one may navigate over the cells, its vefs, cells around these vefs, etc., using fe_space_t iterators all the way round.

### Global DOF Numbering Generation

In this section, we discuss how fe_space_t coordinates the building blocks covered so far in order to generate a *global* enumeration of the DOFs describing the global FE space $\mathcal {X}_h \, \doteq \, \mathcal {X}^1_h \times \ldots \times \mathcal {X}^n_h$ for general multi-field systems of PDEs. This process is encompassed within the generate_global_dof_numbering binding of fe_space_t (see Listing 27). The code of this method is shown in Listing 33. The block_layout dummy argument lets the caller to customize the global DOF numbering to be generated.^29^ On the one hand, this data type specifies in how many blocks the user wants to split the (discrete) PDE system at hand. In particular, the user may select to generate a DOF numbering suitable for monolithic or blocked storage linear algebra data structures, with block_layout%get_num_blocks() returning one and a number larger than one, respectively. On the other hand, block_layout_t specifies the mapping of fields into blocks, with block_layout%get_block_id(field_id) returning the block identifier the field with identifier field_id is mapped to. Provided that blocked linear algebra data structures in FEMPAR are addressed using row/column identifiers that are local to each block, block_layout equips the subroutine with the input necessary to generate a block-aware global DOF numbering, in which the DOFs belonging to fields of the first block are numbered first, followed by the ones of the second, and so on. We note that block_layout_t also holds inside how many DOFs are there per block (see Sect. 11.3). These latter quantities are computed within generate_global_dof_numbering (see discussion in the sequel).

**Figure:**
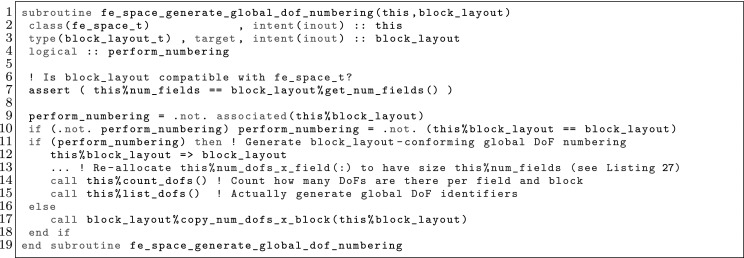
Listing 33. The generate_global_dof_numbering binding of fe_space_t.

The subroutine in Listing 33 starts checking whether it has to actually generate a global DOF numbering. It has to do so if there is no global DOF numbering available yet (see predicate in Line 9), or if the one available is not suitable for the input block_layout (see predicate in Line 10). The bulk of generate_global_dof_numbering is concentrated in the private helper TBPs of fe_space_t called fe_space_count_dofs and fe_space_list_dofs; see Lines 14 and 15 of Listing 33, respectively. The code of these bindings is shown in Listings 34 and 35, respectively. While the former computes the number of DOFs per field and block, the latter is in charge of the actual generation of the global DOF identifiers.

**Figure:**
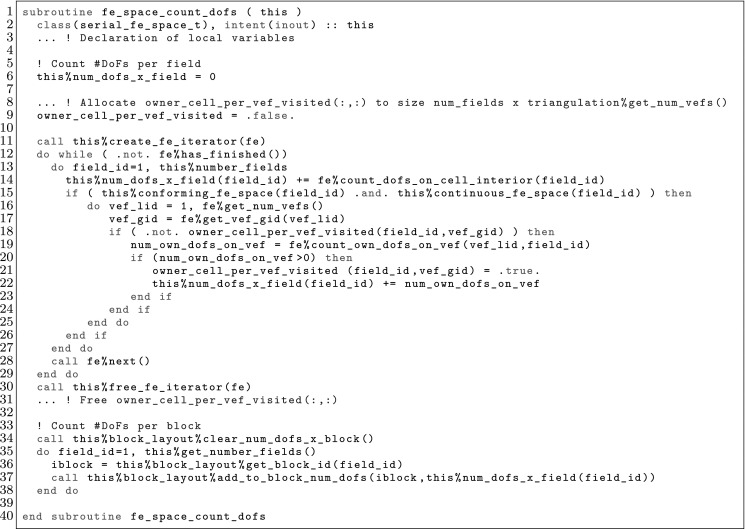
Listing 34. The count_dofs binding of fe_space_t.

Lines 6–31 of Listing 34 are in charge of computing the number of DOFs per field, while those in Lines-34–38, those per block. The latter lines just determine the number of DOFs per block by accumulating those corresponding to fields mapped to the block (computed in the former lines). The former lines are grounded on the notion of *owner cell of a vef*; a cell is the owner of a vef if (1) the latter lays on the boundary of the former, (2) it is the *first cell* for which (1) holds in the order in which the iterator over all cells presents them, and (3) the vef owns at least one DOF of the global FE space subject to consideration.^30^ The (logical) work array owner_cell_per_vef_visited(:) keeps track whether the owner cell of the vefs have been already visited (or not) as these are traversed in the nested loop over all cells (see outer loop in Line 12), and over all vefs within the current cell (see inner loop in Line 16). Sitting on a cell, the algorithm first counts those DOFs associated to node functionals logically placed in the interior of the current cell (see line 14). It then loops over the vefs of the current cell. If the owner cell of the current vef has not been visited yet, and the current cell is its owner, then the current cell is registered as the owner of the cell, and the DOFs associated to node functionals logically placed on this vef within the current cell are counted in Line 22. Provided that non-conforming FE spaces do not have DOFs on vefs, we can skip the loop over the vefs of a cell and accelerate the process in this case (see the if clause in Line 15 of Listing 34).

The algorithm shown in Listing 35 is in charge of the actual generation of the global DOF identifiers. The work array owner_cell_gid_per_vef(:,:) is used to store the owner cell global identifier of the vefs. On the other hand, vef_lid_in_owner_cell(:,:) array is used as an accelerator lookup table that stores the vef local identifiers (i.e., vef_lid) within their corresponding owner cells if they have been already visited, and -1 otherwise. Both arrays are indexed using vef global identifiers (i.e., vef_gid). Sitting on a cell, the algorithm first allocates global DOF identifiers for all node functionals associated to the interior of the current cell starting from fields_current_dof(field_id), i.e., the next freely available global identifier; see Line 27. It then loops over the vefs of the current cell. If the current vef has not been visited yet, then the current cell becomes its owner, and both the cell and the local identifier of this vef within the cell are registered in the corresponding work arrays. The global DOF identifiers associated to node functionals on this vef within the owner cell are allocated in Line 32 (as above starting from fields_current_dof(field_id)). On the other hand, if the current vef has been visited, then the global DOF identifiers associated to node functionals on this vef within the current cell are fetched from the corresponding ones within the owner cell in Line 39. The binding called in this line encodes the permutations described in Sect. 3.16.

**Figure:**
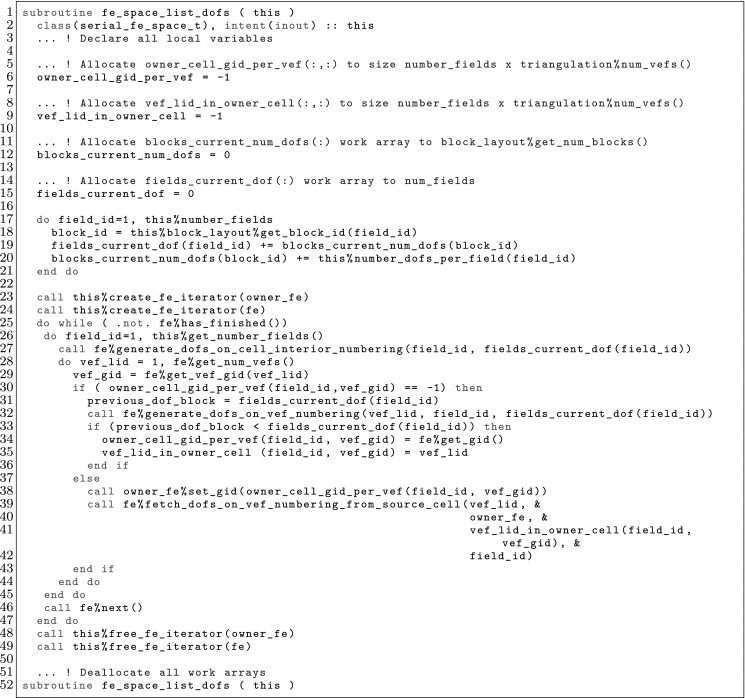
Listing 35. The list_dofs binding of fe_space_t.

As the reader might observe, Listing 35 is grounded on several (private) helper bindings of fe_cell_iterator_t that, at the cell level, aid in the generation of a global DOF numbering; see Lines 9–13 of Listing 30. These bindings ultimately rely on the reference_fe_t instances mapped to the cells of the triangulation; see Sect. 10.1. In particular, sitting on a cell, reference_fe_t instructs fe_cell_iterator_t with the association of its node functionals to the interior of the cell, and its lower-dimensional boundary objects according to the notion of conformity underlying the FE space at hand; see Sects. 3.6 and 6.2. For example, the implementation of the generate_own_dofs_vef_numbering binding is implemented as shown in Listing 36.

**Figure:**
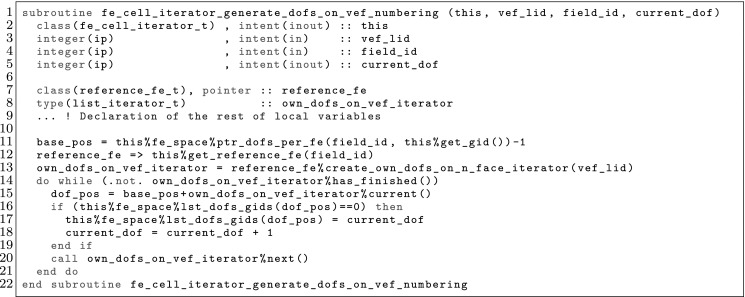
Listing 36. Implementation of the generate_own_dofs_vef_numbering binding of fe_cell_iterator_t.

The code in Listing 36 extracts a list_iterator_t from the own_dofs_n_face member variable of the reference_fe_t instance used in the current cell for field_id. This iterator lets it to traverse those node functionals owned by the vef with local identifier vef_lid (see Sect. 6.2), and thus determine the (relative) position in lst_dofs_gids(:) of the global DOF identifiers to be allocated for such node functionals. We note that the logical predicate in Line 16 is evaluated to .true. if the DOF at hand is actually free, i.e., not subject to boundary conditions imposed in strong form; see Sect. 10.4.

Finally, we would like to stress that error checking statements and a major optimization that can be applied for the single-field single-block case are not shown in the code listings of this section in order to keep the presentation as simple as possible. Both are present in FEMPAR. In particular, for the aforementioned case, the global DOF numbering can be generated with a single loop over all cells (instead of two). The call in Line 14 of Listing 33 can be avoided, deferring the computation of the number of DOFs per field and block to the call in Line 15.

On the other hand, there is no need to generate a global DOF numbering from scratch when there is already one available, a permutation from the old to the new numbering could be computed and applied to lst_dofs_gids(:) by a single sweep over all cells. This optimization, however, is not present in FEMPAR, as indeed we did not find frequent the case where an application requires to change on-the-fly the block-layout of the system of PDEs at hand.

### Strong Imposition of Boundary Conditions

In this section, we discuss the mechanisms that fe_space_t provides in order to support the strong imposition of boundary conditions. In order to grasp why these mechanisms are needed and how fe_space_t is designed to provide them, we must first briefly introduce the approach chosen by FEMPAR in order to handle this type of boundary conditions. We will use the term “fixed DOFs” to refer to those DOFs sitting on the boundary whose values are constrained (i.e., subject to strong boundary conditions), and the term “free DOFs” to refer to the remaining ones. For simplicity, let us restrict ourselves to the Laplacian problem with inhomogeneous Dirichlet boundary conditions $u(x)=u_{\mathrm{D}}(x)$ on ${\Gamma _\mathrm{D}}$ discretized with grad-conforming FEs.^31^ The discrete solution $u_h \in \mathcal {X}_h$ can be split into two parts as $u_h = {\bar{u}}_h + E_h u_{\mathrm{D}}$, where:

$$\begin{aligned} {{\bar{u}}}_h= & {} \ \sum _{{a \in \mathrm{\{free\ {DOFs} \}}}} {\bar{u}}_a \phi ^{a} \ \ + \ \ \sum _{{a \in \mathrm{\{fixed\ {DOFs} \}}}} 0 \phi ^{a} \quad \quad \mathrm{and} \quad \quad E_h u_{\mathrm{D}} \ = \ \sum _{{a \in \mathrm{\{free\ {DOFs} \}}}} 0 \phi ^{a} \ \ \nonumber \\&+ \ \ \sum _{{a \in \mathrm{\{fixed\ {DOFs} \}}}} u_{a}^{\mathrm{D}} \phi ^{a} . \end{aligned}$$

The nodal values ${\bar{u}}_a$ are the actual unknowns of the problem at hand. $E_h u_{\mathrm{D}}$ is a discrete Dirichlet data extension, which can be understood as the projection of a Dirichlet data extension $Eu_\mathrm{D}(x)$ introduced in Sect. 3.1. Its nodal values $u_{a}^{\mathrm{D}}$ are selected such that $E_h u_{\mathrm{D}}$ becomes a suitable boundary FE approximation of $u_{\mathrm{D}}(x)$ (e.g., a boundary FE interpolation).^32^ The linear system to be solved in order to compute the nodal values of ${\bar{u}}_h$ can be written as:

33 $$\begin{aligned} \sum _{{b \in \mathrm{\{free\ {DOFs} \}}}} a(\phi ^{a},\phi ^{b}) {\bar{u}}_b = (\phi ^{a},f)-\sum _{{c \in \mathrm{\{fixed\ {DOFs} \}}}} a(\phi ^{a},\phi ^{c}) u_{c}^{\mathrm{D}} \quad \forall a \in \{\mathrm{free \ {DOFs} }\}, \end{aligned}$$

where *its coefficient matrix has as many rows as free DOFs*, and its right-hand side is the FE discretization of the linear form in (3); see Sect. 3.1.

In order to assemble (33), the process described in Sect. 8 has to be slightly modified. A sweep over all triangulation cells is still required. Sitting on a given cell *K*, the element matrix $\mathbf {A}^{K}$ and vector $\mathbf {f}^{K}$ are computed as usual. However, the rows/columns corresponding to fixed DOFs in $\mathbf {A}^{K}$ are not assembled into the global matrix. The same applies to the entries of $\mathbf {f}^{K}$. However, $\mathbf {f}^{K}$ has to be updated before assembly in order to reflect the contributions of strong boundary conditions (see the right-hand side of (33)). Fortunately, the users of FEMPAR are unburdened from these subtleties. These are hidden within the assembly generic binding of fe_cell_iterator_t; see Listing 30 and 39. Apart from adding the contributions of the current cell to the global coefficient linear system and right-hand side, this binding is in charge of computing the contribution to $\mathbf {f}^{K}$ from strong Dirichlet boundary conditions. This poses two additional requirements on fe_space_t. In particular, (1) it should handle a global enumeration of free and *fixed* DOFs, while being able to distinguish among both kinds of DOFs; and (2) it should offer a suitable set of bindings to project/interpolate $u_{\mathrm{D}}(x)$ on the boundary to get $E_h u_{\mathrm{D}}$.

In order to satisfy (1), fe_space_t splits the whole set of DOFs into free and fixed DOFs, and the DOFs within each subset are labeled separately from each other as $\{1,2,\ldots ,|\{\mathrm{free \ {DOFs} }\}|\}$, and $\{-1,-2,\ldots ,-|\{\mathrm{fixed \ {DOFs} }\}|\}$, respectively. (This is nevertheless an implementation detail that is never exposed to FEMPAR users.) In turn, free and fixed DOF values are actually stored into different arrays, so that they can be addressed separately using the corresponding global identifiers in the former and latter set, respectively; see Sect. 10.5.

The process that associates global identifiers to free DOFs has been already covered in Sect. 10.3. The one corresponding to fixed DOFs very much resembles the one for free DOFs. It is, however, restricted to vertices, edges, and faces of the triangulation that lay at the boundary, and it has to be equipped with support from the user that lets the process become aware of which DOFs sitting on the boundary are actually fixed. The fixed DOFs global enumeration process occurs during the initial set-up of fe_space_t; see create generic binding in Listing 27. This process is grounded on two different ingredients. On the one hand, the user can determine $\Gamma$ sub-regions through the sets associated to vefs sitting on the boundary (see Sect. 7.1). For example, the user may decide to use set identifier 1 and 2 to split the vefs in $\Gamma$ into those which belong to ${\Gamma _\mathrm{D}}$ and ${\Gamma _\mathrm{N}}$, respectively. On the other hand, an abstract data type, called conditions_t, to be extended by FEMPAR users, lets users to customize the strong imposition of boundary conditions. In particular, with regard to the fixed DOFs global enumeration process, this data type offers a deferred binding that given a set identifier, provides a logical component mask. For each component of the PDE system, this mask provides whether the DOFs associated to vefs marked with this set identifier are fixed or free. For those FE spaces for which there is no DOF-to-component association (e.g., Raviart-Thomas or Nédélec FEs), only the first component in the mask is taken into account, and the rest neglected.

On the other hand, for 2), fe_space_t provides a set of methods that let the user interpolate/project $u_{\mathrm{D}}(x)$ on the boundary to get $E_h u_{\mathrm{D}}$ in a number of suitable ways. $E_h u_{\mathrm{D}}$ is ultimately stored within an instance of the fe_function_t data type; see Sect. 10.5. Boundary projectors involve the solution of a boundary mass matrix problem where integrals over boundary facets have to be evaluated; see Sect. 9. Again, all these bindings rely on the conditions_t abstract data type. In particular, given a boundary vef set identifier, a deferred binding of this data type returns a user-defined (scalar-valued) function to be imposed for each component of the PDE system at hand. In the case of Raviart-Thomas or Nédélec FEs, the *d* scalar-valued functions corresponding to its components are used to reconstruct the vector-valued function, whose tangential or normal component, respectively is to be imposed.

### Global FE Functions and Their Restriction to Triangulation Cells/Facets

In this section, we introduce a convenient software abstraction in our OO design, referred to as fe_function_t, which represents a global FE function $u_h \in \mathcal {X}\, \doteq \, \mathcal {X}^1_h \times \ldots \times \mathcal {X}^n_h$. This data type and a subset of its TBPs (in particular, those that are relevant for the present section) are presented in Listing 37.

**Figure:**
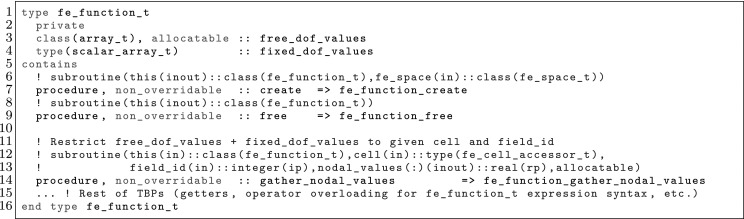
Listing 37. The fe_function_t data type.

In Listing 37, the free_dof_values and fixed_dof_values are used to store ${\bar{u}}_h$ and $E_h u_{\mathrm{D}}$, respectively; see Sect. 10.4. The former is a *polymorphic* member variable of type array_t; see Sect. 11.1. Relying on the set of deferred bindings offered by array_t, the code bounded to fe_function_t can be written independently of how the entries within the concrete implementation of array_t are laid out in memory, enabling code re-use to a large extent. For example, scalar_array_t is a concrete realization of array_t that uses monolithic storage, while block_array_t stores the entries organized into blocks (see Sect. 11.1 for more details). On the other hand, fixed_dof_values is a member variable of *static* type scalar_array_t; see Sect. 11.1.^33^ Fixed DOFs belonging to different fields might be indeed assigned intermixed global identifiers, significantly simplifying the enumeration process. In particular, a single sweep over all boundary objects suffices, in contrast to Listing 33, where two sweeps over all cells are required in order to generate a block-aware global numbering. From our experience, it turns out that neither blocked storage nor a data structure suitable for distributed-memory environments are strictly required to store $E_h u_{\mathrm{D}}$, so that we can prevent the overhead associated to run-time polymorphism when dealing with fixed_dof_values.^34^

A fe_function_t instance is created from a fe_space_t instance (to which it belongs); see signature of the create binding in Listing 37. This binding selects the dynamic type of free_dof_values, and therefore its storage layout, according to the one currently selected for the PDE system at hand; see block_layout member variable in Listing 27. The entries of free_dof_values can be determined in a number of ways. They might become the unknowns of a problem to be solved (e.g., by a preconditioned iterative linear solver or sparse direct solver), or computed from an expression involving other fe_function_t instances, e.g., $u_h=v_h$, or $u_h=v_h+w_h$, with $u_h,v_h,w_h \in \mathcal {X}_h$. (Indeed, FEMPAR offers an expression syntax for global FE functions grounded on overloaded operators.) Apart from these, fe_space_t offers a pair of generic bindings, referred to as interpolate and project, to compute the DOFs nodal values of ${u}_h$ by either interpolation (using the expression in (9)) or projection (e.g., a global $L^2$ projection) into the FE space of a user-defined function *u*(*x*).^35^ Each of these generic bindings is overloaded with three different regular bindings suitable for scalar, vector, and tensor-valued functions, respectively. The interpolate bindings in fe_space_t can be written independently of the reference FE by using a TBP associated to reference_fe_t that computes the local interpolator in (6).

Apart from the software representation of a global FE function, FE codes typically need a mechanism that, sitting on a cell or facet of the triangulation, provides the values, gradients, etc. of a global FE function $u_h=u^1_h \times \ldots \times u^n_h$ evaluated at quadrature points in the physical space. To this end, FEMPAR offers a set of data types, referred to as cell_fe_function_type_t and facet_fe_function_type_t, with type=scalar,vector,tensor, that represent the restriction of $u^i_h$ to a given triangulation cell and facet, respectively. The two code snippets in Fig. 9 illustrate the usage of these data types, where we are assuming that $u^i_h$ belongs to a global FE space of vector-valued functions.

**Fig. 9:**
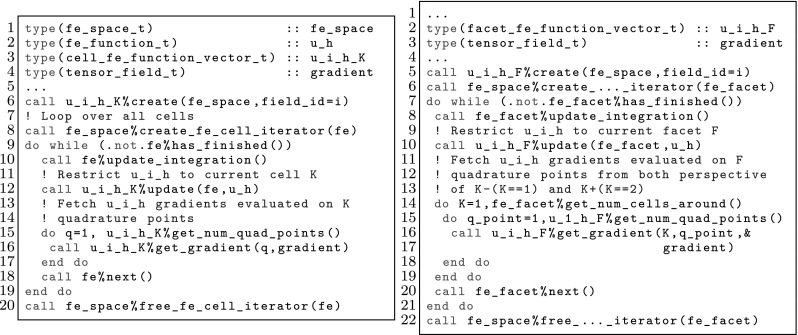
User-level code snippets illustrating the usage of the cell_fe_function_type_t (left) and facet_fe_function_type_t (right) data types

There are three worth noting remarks in these two code snippets. First, the update binding of both data types rely on the gather_nodal_values binding of fe_function_t; see Listing 37. The latter equips cell/facet FE functions with the ability to restrict (gather) the nodal values of $u^i_h$ from global to local arrays (stored as private scratch data within cell/facet FE function data types), *while taking care of strong boundary conditions*. Second, the update bindings require a procedure that, given the shape functions, first derivatives, etc., evaluated at quadrature points in physical space, and the nodal values $u^i_h$ restricted to the current cell, provides the shape function values, gradients, curls, etc., of the FE function at these quadrature points. This service is provided by reference_fe_t by the set of evaluate_fe_function... deferred bindings in Lines 63–68 of Listing 6. We note that fe_function_t can extract the first set of data from the cell_integrator_t and facet_integrator_t instances accessible through fe_cell_iterator_t and fe_facet_iterator_t (provided on input to update), respectively. Third, facet FE functions provide $u^i_h$ values, gradients, etc., at facet quadrature points from the perspective of its two surrounding cells. This make sense for functions $u^i_h$ belonging to non-conforming FE spaces, which might be discontinuous across cell boundaries. Facet FE functions should also cope with the fact that the coordinate systems of its surrounding cells might not be aligned in physical space, so that a different local numbering might be assigned to facet quadrature points from the perspective of either cell; see Sect. 9.3 for an exposition of the strategy followed to solve this issue.

## Building FE Affine Operators

**Fig. 10:**
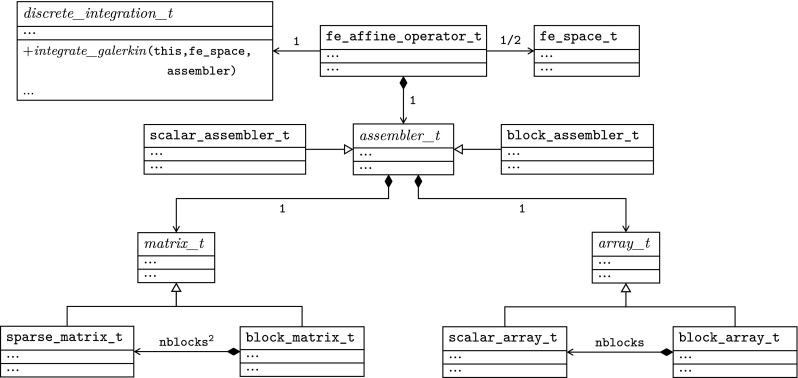
UML class diagram of the fe_affine_operator_t abstraction and its relationship with other FEMPAR classes

In this section, we introduce the software abstractions on which the construction of the algebraic problem (10) in Sect. 3 relies. These software abstractions, and their relationship, are depicted in Fig. 10. The main design goal underlying the proposed software architecture is as follows. In the seek of code reusability and extensibility, FEMPAR users should have at their disposal *a unique entry point data type and associated bindings* in order to build their FE linear system, no matter whether a scalar or a system of PDEs, no matter whether the linear algebra data structures holding the linear system entries are either scalar (monolithic) or blocked, and no matter how they are laid out in memory (centralized, distributed-memory). In FEMPAR, this unique entry point data type is referred to as fe_affine_operator_t. Mathematically, fe_affine_operator_t represents the affine operator in (5), obtained from the discrete weak formulation of the linear(ized) problem (4). As introduced in Sect. 3.6, the operator can be represented (after defining bases for trial and test FE spaces) with $\mathbf {A}$ and $\mathbf {f}$ defined in (10). The solution of the FE problem is the only root of this operator (as soon as the FE problem is nonsingular).

In order to seek the aforementioned goal, fe_affine_operator_t relies on an abstract data type, referred to as assembler_t (see Fig. 10). In a nutshell, assembler_t offers a set of *FE-assembly tailored*, data structure neutral, deferred TBPs, e.g., to assemble the contributions of a cell or facet integral into the linear system coefficient matrix $\mathbf {A}$ and/or right-hand side $\mathbf {f}$. The subclasses of assembler_t are the ones ultimately responsible to deal with the details underlying the particular linear algebra data structures at hand. The latter ones offer *FE-assembly neutral* interfaces to inject new entries or add contributions to them, such that this software piece becomes reusable and separable, e.g., to be used in third party software projects (not necessarily FE-oriented) as a standalone software subsystem. FEMPAR offers a rich set of linear algebra data structures, e.g., data structures organized by blocks, which enable the implementation of block preconditioners for multiphysics problems (see, e.g., [43–45]). Apart from those required to handle the linear coefficient matrix and right-hand side of the system, fe_affine_operator_t also interacts with other data types required to deliver its life cycle (i.e., its auto-generation). In particular, $\mathbf {A}$ and $\mathbf {f}$ entries are computed according to the expressions in (10). These expressions involve a FE space (fe_space_t) and the discrete (bi)linear forms of the problem at hand. To express in software this second ingredient, we introduce the discrete_integration_t abstraction; see Fig. 10.

We have structured this section as follows. In Sect. 11.1, we first present the assembler_t abstract data type, and the rationale underlying the design of the linear algebra structures it is grounded on. Next, in Sect. 11.2, we introduce the discrete_integration_t abstract data type that ultimately is in charge of performing the integration of the (bi)linear forms and assembly of the discrete affine operator. We show a particular implementation of this data type (i.e., a subclass) for the Galerkin approximation of the Stokes problem. Finally, the fe_affine_operator_t data type is described in Sect. 11.3.

### Linear Algebra Data Structures and Associated Assemblers

Linear algebra in FEMPAR relies on a pair of data type hierarchies rooted at the mathematical abstractions of a linear algebra operator and a vector, and represented in software by means of the linear_operator_t and vector_t abstract data types, respectively. These abstract data types let a number of linear algebra algorithms within FEMPAR (e.g., iterative linear solvers and block preconditioners for PDE systems) to be expressed independently from the actual implementation of the concrete matrix and vector data structures being used, such as block layout (if any), storage (e.g., dense or sparse storage format) or memory layout (e.g., local or distributed-memory), enabling code re-use and extensibility to a large extent. An abstract expression syntax that allows the construction of complex expressions involving operations among operators and/or vectors is also provided. This enables the implementation of new algorithms in a compact manner. However, because these linear algebra algorithms are not discussed herein but postponed to a further work, the description of the data types and associated methods in these hierarchies will be restricted to what is necessary to describe the assembly of the FE affine operator.

The sparse_matrix_t data type can be found at an intermediate level in the hierarchy rooted at linear_operator_t. This is a crucial data type in FEMPAR, which represents a scalar, non-distributed, sparse matrix. Its design follows the ideas presented in [92]. This design (re)uses the *state* OO design pattern [88] to hide the actual sparse matrix storage format to the user. Following this pattern, sparse_matrix_t is composed of a polymorphic member variable of (declared) type base_sparse_matrix_t. Its dynamic type can be thus changed at runtime (via re-allocation). This dynamic type represents the storage at hand being used. Current subclasses of base_sparse_matrix_t include coo_sparse_matrix_t, csr_sparse_matrix_t, csc_sparse_matrix_t, corresponding to the coordinate list (COO), the compressed sparse row (CSR), and the compressed sparse column (CSC) sparse matrix storage formats [93], respectively.

The life cycle of a sparse_matrix_t instance is as follows. The user first invokes its create TBP, in which one solely specifies the size of the matrix, i.e., the number of rows and columns. This method, however, triggers a number of subsequent actions. In particular, *it allocates its dynamic type to the one corresponding to the COO format*, and leaves it ready for the injection or addition of contributions to the entries of the matrix. Although not compressed, this format is ideally shaped for the injection or addition of contributions to the entries of the matrix. These are simply pushed back into member arrays that can grow dynamically during the integration/assembly loop (via a judiciously reallocation strategy to trade off cost and memory). Other sparse storage formats, as the CSR storage implemented in the csr_sparse_matrix_t data type (also a type extension of base_sparse_matrix_t), although more memory efficient, require a predefined sparsity pattern, which has to be precomputed. They are not thus well suited for the dynamic build up process of the matrix. At this point the reader should note that, for such inflexible storage formats, *one typically needs an accurate estimation of the maximum number of nonzeros per each row (or column) to be memory efficient*. This estimation, however, can only be a quite large upper bound in complex scenarios (e.g., *hp*-adaptive methods in 3D, among others).

Once the build up process finishes, the user can call a method specially designed to leave the sparse_matrix_t instance ready for being used (e.g., to perform operations with it). This involves a compression process, in which duplicated entries are either summed up, or filtered (as selected by the user) and a transformation of the COO storage format into the storage format that the user actually requires (e.g., CSR). For simplicity, we refer to this stage as the “compression” of the matrix. Once the sparse_matrix_t instance is in this final state, it is still possible to insert or add contributions to its entries, as far as they belong to the sparsity pattern resulting from the first build up process. Thus, e.g., if a transient and/or nonlinear problem is to be solved and the triangulation of the domain does not change, the assembly in COO format will only be performed at the first nonlinear iteration of the first time step.

As shown so far, the software architecture of sparse_matrix_t is such that several (current and future) storage formats are possible within a single framework. This flexibility is convenient for two main reasons. First, no given storage format is likely to be uniformly better in performance across all possible operations and computer architectures. Second, FEMPAR interoperability with external software dramatically increases. If a new library, that uses its own storage format, is to be integrated, only a new extension of base_sparse_matrix_t has to be added, while leveraging dozens of thousands of lines of code already written. Apart from sparse_matrix_t, there are other sparse matrix data types available, suitable to handle blocks and/or distributed-memory computers. All these data types are essentially composed in some way or another of sparse_matrix_t instances. For example, block_sparse_matrix_t is composed of $\texttt {nblocks}^2$ sparse_matrix_t instances; see Fig. 10. It, however, provides a set of specialized TBPs that only apply in the blocked case, e.g., the get_block TBP that lets a client to retrieve the sparse_matrix_t instance corresponding to a given block of the matrix.

The counterpart of sparse_matrix_t in the vector case is referred to as scalar_array_t. It represents a scalar, non-distributed, linear algebra vector, with its entries stored explicitly in a simple (Fortran intrinsic) allocatable array. However, provided that it does not have to exploit sparsity, the code bounded to this data type is significantly simpler to the one bounded to sparse_matrix_t. It is equipped with a pair of generic bindings, with signatures coming in different flavours, in order to insert or add contributions to the vector. Likewise, there are other vector-like data types available suitable to handle blocks and/or distributed-memory computers. For example, block_array_t is composed of $\texttt {nblocks}$ scalar_array_t instances; see Fig. 10.

Apart from the linear algebra data structures so far, we need the additional data type assembler_t, which offers FE-assembly tailored signatures to fe_affine_operator_t. The interface of its deferred TBPs, which its extensions, e.g., scalar_assembler_t and block...assembler_t, implement, are shown in Listing 38. assembler_t has to be “general enough” to handle many storage layouts and it is in charge to isolate fe_affine_operator_t from implementation details. With that purpose in mind, it is composed of a (polymorphic) matrix_t and a (polymorphic) array_t instance. These are in turn abstract data types rooted at all the matrix and array data types seen so far, respectively. The set of deferred TBPs of these two abstract data types is designed (on purpose) to be not sufficiently rich to handle the whole life cycle of the concrete matrix and array instances. The high heterogeneity of the concrete subclasses of matrix_t and array_t precludes it. This set of TBPs is, in particular, restricted to allocation of memory for its entries, initialization of its entries to a given value (e.g., initialization to zero), and deallocation of any internal memory. These three operations are required by fe_affine_operator_t during the deployment of its life cycle. The bulk of the life cycle of the concrete subclasses of matrix_t and array_t is handled by the subclasses of assembler_t. This is how it should be, provided that assembler_t subclasses are the ones aware of the concrete details of the corresponding matrix_t and array_t subclasses. Besides, by doing this, we can overcome the overhead associated to dynamic run-time polymorphism, provided that the binding of fine-grain calls to those TBPs injecting or adding contributions to the matrix or the array *can be determined at compilation time*.

**Figure:**
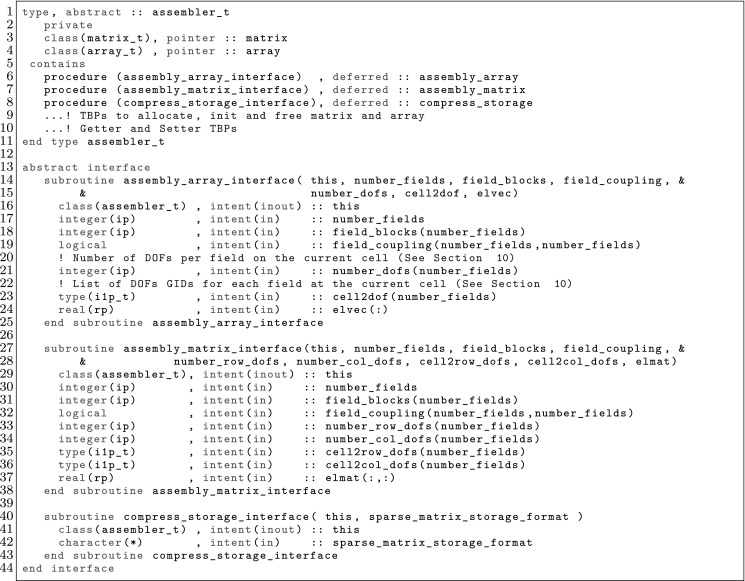
Listing 38. The assembler_t abstract data type and its deferred TBPs.

Going back to Listing 38, observe that assembly_array (resp., assembly_matrix) takes an intrinsic Fortran array (resp., rank-2 array) as dummy argument for the element vector (resp., matrix). Besides, it also gets the global DOFs identifiers on top a single cell, or those corresponding to cells surrounding the facet (see Lines 23, 35 and 36 in Listing 38). In the case of scalar_assembler_t, the implementation is made using the TBPs provided by scalar_array_t in order to add contributions to its entries and the corresponding TBPs of sparse_matrix_t. In the case of block_assembler_t, the implementation is made by looping through the blocks, obtaining a reference to the current block with the get_block TBP, and using the corresponding TBPs as in the previous case. The assembly_array and assembly_matrix TBPs are used by the fe_cell_iterator_t and fe_facet_iterator_t data types to implement their assembly TBPs (see Lines 35 and 68 in Listing 30 of Sect. 10.2). For completeness, in Listing 39 we show the signature of the latter TBPs. These are the ones actually used by the user in the type extension of discrete_integration_t, as described in Sect. 11.2.

**Figure:**
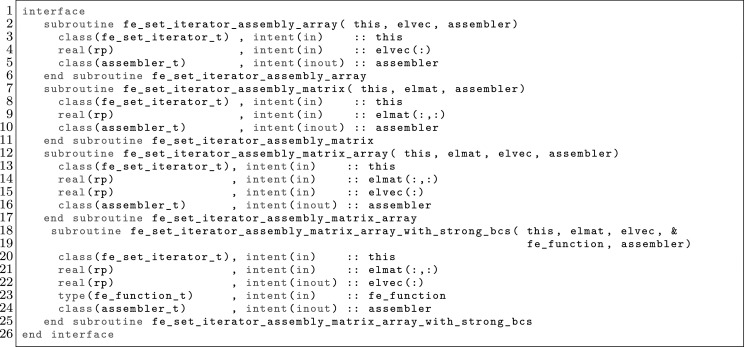
Listing 39. The interfaces of the assembly TBPs of “set” (either cell or facet) iterators.

Finally, the compress_storage deferred TBP of assembler_t lets fe_affine_operator_t to signal that the build up process of the linear algebra data structures has already finished and that they can already be “compressed” into its final stage.

We stress that the software architecture presented in this section provides uniform assembly interfaces to the client that are completely independent of the underlying implementation of linear algebra data structures. The subclasses of assembler_t are in charge of the management of blocks (if any), whereas sparse_matrix_t is in charge of the management of the storage schemes.

### Discrete Integration of FE Operators

In this section, we introduce the abstract data type discrete_integration_t (see Listing 40). It defines the generic integrate binding, which is overloaded by the integrate_galerkin and integrate_petrov_galerkin deferred TBPs, depending on the number of fe_space_t instances being passed to them (see, e.g., Line 8 of Listing 40 for the interface corresponding to the Galerkin case). A user that wants to implement a FE problem must extend this data type and overwrite the TBP to be used (Galerkin or Petrov-Galerkin) in the user-defined subclass. In the overridden method, the user must implement the evaluation of the entries of $\mathbf {A}$ and $\mathbf {f}$ as the numerical integration of the discrete bilinear and linear forms as in (10) (see Sect. 3).

Based on our experience, the integration part of a FE code must exhibit a huge level of flexibility. Every time one wants to consider a new set of PDEs or a new expression of the discrete bilinear form, this component must be modified. It must also have the ability to integrate general time integration schemes that can require functions in an arbitrary number of steps, deal with nonlinear problems that involve the need to evaluating FE functions in the integration of the discrete forms, or including variable physical coefficients of body force terms determined through analytical functions. As a result, any rigidity at this level must be eliminated. Indeed, the discrete_integration_t abstract data type only forces its subclasses to adhere to the signatures of the deferred TBPs overloading integrate, and has no member variables that subclasses are forced to handle. Using the design previously sketched, the user has absolute flexibility to design its own discrete_integration_t subclass, adding the attributes and methods that can be required to integrate and assemble the discrete forms, e.g., by adding an arbitrary number of fe_function_t and *_function_t instances (and corresponding setters to be used at the driver level) that can describe physical properties, previous time step values, the solution at the previous nonlinear iteration, etc.

The integration of cell-wise terms of the (bi)linear forms is accomplished by traversing through all the cells using a fe_cell_iterator instance (see Sect. 10.2), which has access to (1) all the cell integration data (see Sect. 8) required to compute the local cell contributions in (11) and (2) the local-to-global DOF numbering needed for the assembly in the global linear algebra data structures. Analogously, the integration of facet terms, e.g., the ones in (20) for DG formulations, requires the use of a fe_facet_iterator_t instance to traverse through the facets and integrate the corresponding facet terms. The method integrate is called during the execution of the numerical_setup TBP of fe_affine_operator_t. It is in fact the fe_affine_operator_t the one that decides whether to invoke the Galerkin or Petrov-Galerkin integration, depending on whether one or two FE spaces have been passed as actual arguments (the second one being optional) in its create binding (see Line 15 of Listing 42). Analogously, the FE space(s) are also passed as actual argument(s) to the integrate_* bindings, since they will be needed at any integration step (see Line 8 of Listing 40 for the Galerkin case).

**Figure:**
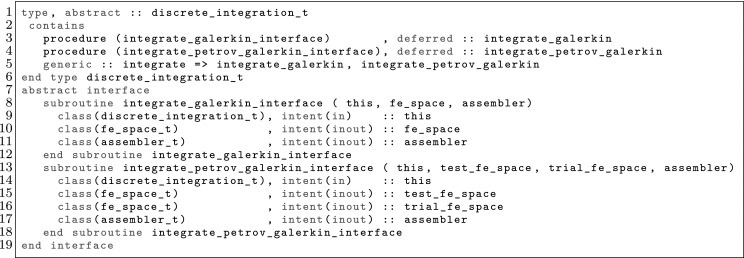
Listing 40. The abstract data type discrete_integration_t and its deferred TBPs.

For illustration purposes, we present in Listing 41 an example extension of discrete_integration_t. It shows the implementation of the deferred procedure integrate_galerkin for the approximation of the Stokes problem using a Galerkin method. This data types will be used in the example driver presented in Sect. 12 for the inf-sup stable Taylor-Hood mixed FE method (see Listing 41).^36^

**Figure:**
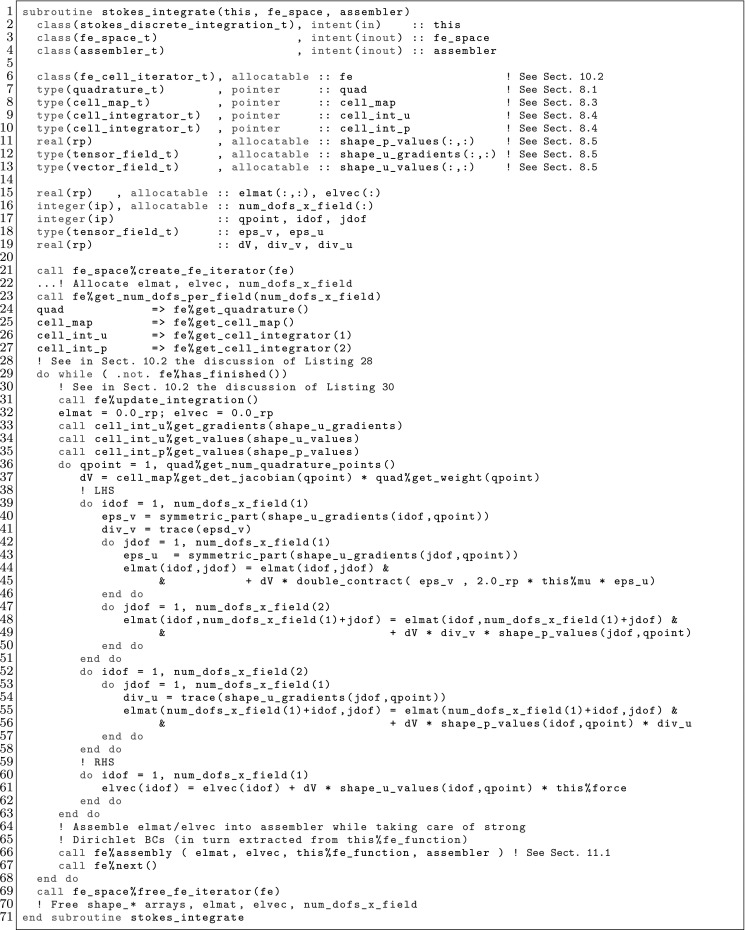
Listing 41. The implementation of a binding that overrrides the integration_galerkin TBP of discrete_integration_t for the Galerkin approximation to the Stokes problem.

As commented above, the integration of the (bi)linear forms requires the cell integration machinery, which is provided by fe_space_t through the creation of the fe_cell_iterator_t in Line 19 of Listing 41. Apart from controlling the loop over cells (Lines 24 and 62), fe_cell_iterator_t provides the numerical quadrature, which is in turn required to get the number of integration points (line 31), and its associated weights (line 32). It also provides the determinant of the Jacobian of the cell map (line 32), and the shape functions and gradients at Lines 28 to 30 (see (13) and (14)). The implementation of the (bi)linear forms is very close to the blackboard expression, making it compact, simple, and intuitive. This is possible through the definition of the vector_field_t, and tensor_field_t data types, together with their corresponding expression syntax available in FEMPAR. As it was carefully discussed in Sect. 8.5, it is achieved using operator overloading for different vector and tensor operations, e.g., the contraction and scaling operations. The symmetric_part (used at Lines 35 and 38), double_contract (used at line 40) and trace helper stand-alone functions (used at Lines 36 and 49) are also offered to make tensor operations easy. We also note that this implementation is also *efficient*, since all these operations are made without any dynamic memory allocation/deallocation.

Finally, the fe_cell_iterator_t also offers a TBP to assemble the element matrix and vector into the assembler and to impose strong Dirichlet conditions (line 66) using the perturbation in (3) (See Sect. 10.4). The Dirichlet data is extracted from a fe_function_t that represents $E_h u_{\mathrm{D}}$, which must be an attribute of the concrete discrete_integration_t. For non-conforming FE spaces, the formulation requires also a loop over the facets to integrate DG terms. It can be written in a similar fashion using the tools described in Sect. 9. In this example, the stokes_galerkin_integration_t extension has the attribute $\texttt {force}$, which is used in Line 56 to integrate the right-hand side. It is a vector field described by an instance of the vector_function_t data type.

### The FE Affine Operator Abstraction

A (simplified) declaration of the fe_affine_operator_t data type is shown in Listing 42. The fe_affine_operator_t is created from a single fe_space_t instance, or even two for Petrov-Galerkin formulations; the second instance is optional and, when it is not passed, the Galerkin method is used, i.e., the same FE space is used for trial and test spaces. The user can (optionally) configure a desired block layout. Given a Cartesian product FE space $\mathcal {X}^1_h \times \ldots \times \mathcal {X}^{n_\mathrm{field}}_h$ for a multi-field problem with $n_\mathrm{field}$ fields (see Sect. 3.11), the block layout represents a partition of fields into subsets.^37^ It is described through the argument array field_blocks of size num_fields equal to $n_\mathrm{field}$, which indicates the block to which each field is assigned; by default, the one-block case is used. e.g., For the Stokes problem in Example 3.2, one can consider a monolithic block layout with only one block that includes both the velocity and pressure field (field_blocks=[1,1]), or two one-field blocks (field_blocks=[1,2] or [2,1]). Additionally, the user must provide additional information about the diagonal blocks, namely (1) whether the block is symmetric or not, (2) whether symmetric storage wants to be used for the block or not, and (3) whether the block is positive definite, semi-positive definite, or indefinite. The user can optionally provide the array of logicals field_coupling (of size num_fields $\times$ num_fields); the position $(\texttt {i},\texttt {j})$ determines whether the matrix entries related to trial/test functions of the FE space $\texttt {i}$ and FE space $\texttt {j}$ are always zero (in this case, the coupling is false) or not. For the Stokes problem and the Galerkin method, the only entry that is false (no coupling) is the pressure-pressure entry. When this array is not provided, the case by default is that all fields are coupled. It only implies more memory consumption, e.g., to store the zero entries in the pressure-pressure block for the Stokes problem.

**Figure:**
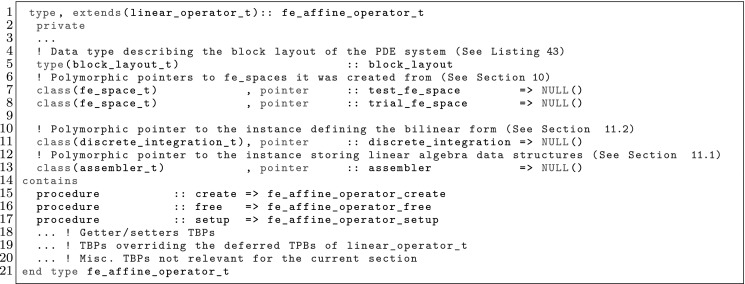
Listing 42. The fe_affine_operator_t data type.

The block layout information is stored in the data type block_layout_t, sketched in Listing 43, which stores the arrays field_blocks and field_coupling. It is created in the binding that creates the fe_affine_operator_t. It also stores a block-wise DOF numbering generated by the fe_space_t instance, which is instructed to do so by passing the block_layout_t 38 when calling its TBP generate_global_dof_numbering, described in Sect. 10.3.

**Figure:**
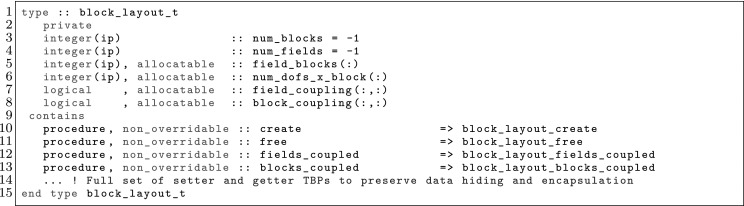
Listing 43. The block_layout_t data type.

The fe_affine_operator_t also holds a polymorphic pointer to an assembler_t instance. Its dynamic type is selected during the creation phase depending on the number of blocks, the storage layout required, and the (parallel or serial) environment. Finally, a polymorphic pointer to an instance of declared type discrete_integration_t is also stored (see line 11 of Listing 42). After the creation phase, the fe_affine_operator_t is ready for its setup. Thanks to the design of the linear algebra data structures in FEMPAR, it does not require a symbolic setup, i.e., to precompute a (potential) sparsity pattern. The numerical_setup TBP at line 17 of Listing 42 calls the integrate_galerkin TBP of discrete_integration when the pointer to trial_fe_space is not associated or integrate_petrov_galerkin otherwise, as discussed in Sect. 11.2.

## Driver Example for the Stokes Problem

In this section, we describe the software architecture of a driver program that approximates the solution of the Stokes problem. To this end, it implements a Galerkin FE method grounded on a “static” (i.e., non-adaptable) conforming mesh and inf-sup stable FE spaces. In particular, we consider a conforming FE space $\varvec{\mathcal {V}}_h \times \mathcal {Q}_h$, where $\varvec{\mathcal {V}}_h$ is a grad-conforming Lagrangian space of order $k+1$, and $\mathcal {Q}_h$, a grad-conforming Lagrangian space of order *k*, i.e., the mixed Taylor-Hood FE [5].^39^

It is up to FEMPAR users to decide how to design the software architecture of their main driver program. Any driver program has nevertheless to follow the typical stages needed in a simulation pipeline based on FEs. In the seek of uniformity, the architecture presented in Listing 44 and 45 is recommended to FEMPAR users. The main program unit relies on a number of driver-level module units, which are not part of the FEMPAR library but developed by the user specifically for the problem at hand. Each of these modules defines a driver-level derived data type and its TBPs. A central derived data type, called stokes_driver_t in this example, is designed to drive all the necessary steps. In particular, it offers a public TBP, called run_simulation, on which the driver program relies to perform the actual simulation. The driver program is therefore as simple and concise as shown in Listing 44.

**Figure:**
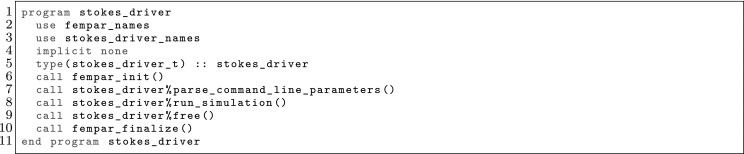
Listing 44. The main program for the solution of the Stokes problem.

**Figure:**
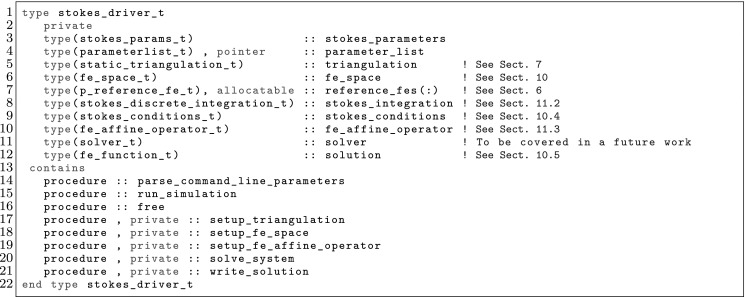
Listing 45. The main data type of the Stokes driver.

The main data type of the driver, stokes_driver_t, is shown in Listing 45. It is equipped with a set of member variables of type already described in previous sections; see comments on the right-hand side of each member variable. The data type solver_t in Line 11 does not exist in FEMPAR as such. There is actually a complete set of data types that provide interfaces to high-end third party sparse direct solvers. Besides, we have developed our own abstract implementation of iterative linear solvers (including, e.g., the conjugate gradient or GMRES Krylov subspace solvers). The convergence of these solvers can be accelerated using advanced preconditioners grounded on the Multilevel Balancing Domain Decomposition by Constraints (MLBDDC) preconditioner [34, 37]. The description of the linear solvers software subsystem deserves considerable space and is postponed to a future work. In this example, it has to be understood as a data type that provides the necessary services required to implement the solve_system TBP at Line 20 of Listing 45. The data type stokes_conditions_t at Line 9 extends conditions_t in Sect. 10.4. It encodes the strong Dirichlet boundary conditions data for this particular operator. The member variable parameter_list (see Line 4) is a parameter dictionary of $<key, value>$ pairs. Its implementation is provided as a stand-alone external software library called FPL [86]. The member variable stokes_parameters (see Line 3) is a user-defined data type that encapsulates the interaction with a command line parser provided by the FLAP software package [94]. Both of them are used to implement the TBP in Line 14, which parses the arguments given by the user in the command line, and transfers them into the aforementioned parameter_list member variable.

The run_simulation TBP (called from the main program in Line 8 of Listing 44) is implemented with the help of the private TBPs in Lines 17–21 of Listing 45. The setup_triangulation TBP invokes the create TBP of static_triangulation_t. Depending on the command-line parameter values, the user may select to automatically generate a structured/uniform triangulation for simple domains (e.g., a unit cube), currently of brick (quadrilateral or hexahedral) cells, or read it from a mesh data file, e.g., using the GiD unstructured mesh generator [91]. The FE space is built in setup_fe_space TBP, sketched in Listing 46.

**Figure:**
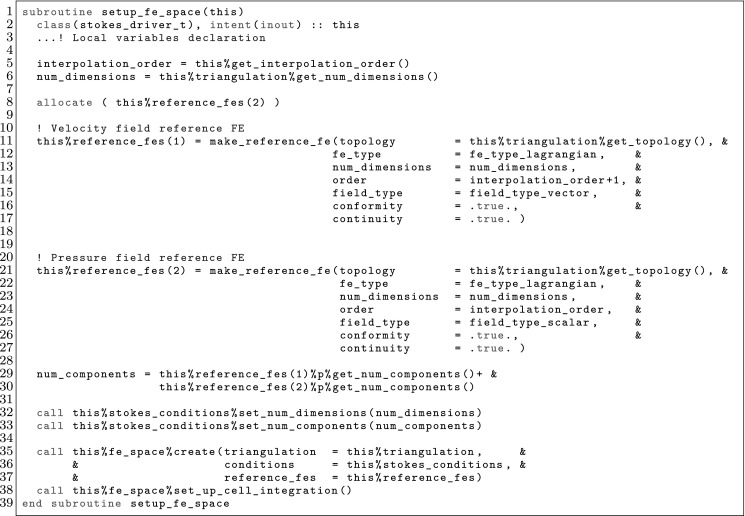
Listing 46. The implementation of the setup_fe_space binding for the Stokes problem.

An array with base type p_reference_fe_t, a data type that wraps a polymorphic pointer to a reference_fe_t instance, is allocated in Line 8 of Listing 46. The reference_fe_t instances for the velocity and pressure fields are created by calling make_reference_fe in Lines 11 and 21, respectively; see Sect. 6.4. The interpolation order of the numerical scheme is read from command-line in Line 5. We select order equal to $k+1$ and *k* in Lines 11 and 21, respectively. The dummy argument continuity determines whether $\mathcal {X}$ admits a trace operator. In this particular example, we could consider continuity=.false. if we wanted to use a discontinuous pressure space. The create TBP of fe_space_t (Line 35) performs the composition of the reference FEs to build the Cartesian product space $\mathcal {X}_h$. Finally, we call the set_up_cell_integration TBP of fe_space_t in Line 38 to set up all the data structures required to evaluate cell integrals in Listing 40.

The implementation of the setup_fe_affine_operator binding is shown in Listing 47. It first invokes the create TBP of fe_affine_operator_t in Line 6. We state monolithic storage for the global coefficient matrix (Line 13), that it is symmetric (Line 9), that we want symmetric storage, i.e., to only store its upper triangle (Line 8), and the fact that it is indefinite (Line 10). The definition of field_coupling in Line 14 reflects that the pressure diagonal block is null. We also pass an instance of fe_space_t in Line 11 and an instance of the subclass stokes_integration_t in Line 12.

**Figure:**
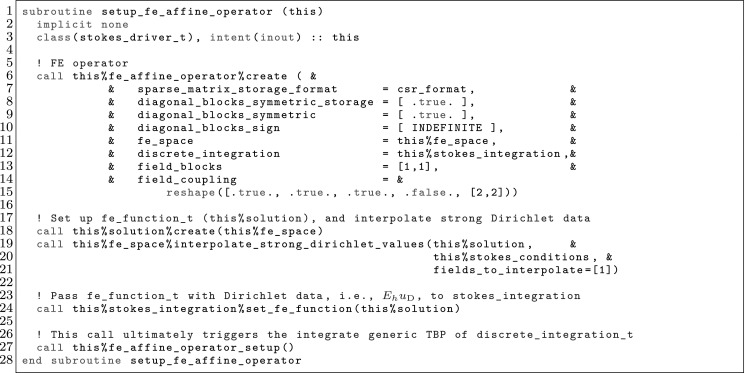
Listing 47. The implementation of the setup_fe_affine_operator binding for the Stokes problem.

Before we set up the operator in Line 27, we create a fe_function_t instance in Line 18. In Line 19, by means of the services provided by fe_space_t, we interpolate the analytical function to be prescribed on the boundary for the velocity field (retrieved from stokes_conditions), fixing the strong Dirichlet DOFs of the fe_function_t instance at hand. As a result, this FE function represents $E_h u_\mathrm{D}$, with the zero extension to free DOFs; see Sect. 10.4. This FE function is passed to the stokes_integration_t instance in Line 24. Finally, we trigger the operator auto-construction in Line 27.

The solve_system TBP (see Line 20 of Listing 45) invokes either a direct or preconditioned iterative solver to obtain the free DOFs nodal values of our FE function (see Sect. 10.5). Provided that this%solution on input to solve_system is such that it vanishes on free DOFs (see discussion in previous paragraph), a common practice used in FEMPAR drivers to save space is to re-use the space devoted for free DOFs in this%solution to store the free DOFs nodal values of the solution of the problem at hand. We stress that all solvers in FEMPAR are such that they only solve for free DOFs. In our experience, this decision dramatically simplifies the development of some preconditioners, provided that they can be developed without taking care of strong Dirichlet boundary conditions.

Finally, the write_solution TBP (see Line 21 of Listing 45) is in charge of the generation of simulation results in data files for later visualization using, e.g., VisIt [95] or Paraview [96]. To this end, write_solution relies on a format independent, extensible abstraction, referred to as output_handler_t. It lets the user to register an arbitrary number of FE functions (together with the corresponding FE space these functions were generated from) and cell data arrays (e.g., material properties or error estimator indicators), to be output in the appropriate format for later visualization. Among its responsibilities, this (abstract) data type generates the data to be written to the (potentially parallel-distributed) file system in neutral, cell-oriented data structures, dealing with (potentially) non-conforming (discontinuous), and variable degree FE spaces among cells. The user may also select to apply a differential operator to the FE function, such as divergence, gradient or curl, which involve further calculations to be performed on each cell, or to customize those cells to be output (e.g., only those that belong to the interior of the geometry in unfitted FE simulations) via their own implementation of cell iterators.

The generation of the actual data files in the appropriate format is in charge of the implementations (extensions) of output_handler_t. FEMPAR currently offers two implementations of output_handler_t (although many others could be implemented as well by the growing community of FEMPAR developers given the extensible software architecture designed). vtk_output_handler_t lets the user to generate their data in the standard-open model VTK [97]. It currently relies on Lib_VTK_IO [98], which (by now) does not actually exploit parallel MPI I/O but instead uses a naive single file per MPI task scheme. vtk_output_handler_t is therefore the recommended option for serial computations or parallel computations on a moderate number of processors. The second one, xh5_output_handler_t, lets the user generate their data in XDMF [99]. XDMF separates the description of the raw data, referred to as “light data”, from the data itself, referred to as “heavy data”. The light data is expressed using a set of XML-based constructs that are suited to represent the distributed-memory data structures in FEMPAR. XDMF in turn supports the heavy data to be stored using HDF5 [100]. HDF5 is, among others, a data model and file format designed with the parallel I/O data challenge in mind. By means of a set of supporting open source libraries, referred to as parallel HDF5 libraries, FEMPAR takes advantage of the underlying distributed file system without having to deal with the high complexity of other lower-level implementations, such as raw MPI I/O. In particular, the latter service is provided by XH5For [101], a stand-alone software library, which we developed from scratch, and lets the user to read/write parallel partitioned FEM meshes taking advantage of the Collective/Independent MPI-IO provided by the PHDF5 library for the efficient generation of the vast amount of data typically resulting from a large-scale scientific computing simulation.

## Conclusions

In this work, we have thoroughly described the approach that we have followed in FEMPAR in order to abstract in software the numerical approximation of problems governed by PDEs using FE methods. The mathematical framework of FEs has been split into a number of (mathematically motivated) derived data types and their interaction, resulting into a well-separated, robust, and stable set of customizable software abstractions for the development of widely applicable FE solvers. These tools equip FEMPAR users with the machinery needed to perform all the steps in the simulation pipeline, including mesh import/generation, DOFs enumeration, evaluation/assembly of the algebraic system of linear equations via FE integration, solution of the linear system, and output of computational results in the appropriate format for later visualization. In order to achieve this goal, the software architecture of FEMPAR has been thoroughly designed by means of advanced OO software re-engineering techniques (including the recurrent application of OO design patterns [85, 88]) in order to increase its ease of use, extensibility, flexibility, and reusability. FEMPAR software architecture has been implemented using the latest OO features of the Fortran03/08 standard, namely, *information hiding and data encapsulation*, *inheritance via type extension*, and *dynamic run-time polymorphism*. This version of the Fortran standard is already widely (and robustly) supported by most of the compilers typically available on high-end computing environments. A judiciously set of programming techniques let us achieve a reasonable trade-off among extensibility and performance, while avoiding in most cases the computational overheads frequently associated with abstract OO software libraries.

The software abstractions covered in this work include:

- The definition of reference FEs, which relies on the concept of polytopes to define the cell topology in arbitrary dimensions, a machinery to define multi-dimensional polynomial functions of arbitrary order in an easy and automatic way, and a general procedure for the generation of the shape function bases and local DOFs.
- The global FE space abstraction, which relies on reference FE(s) and a triangulation of the physical domain. It is responsible to define the local-to-global DOF numbering, which must respect conformity (if needed). The FE space also provides tools for the numerical integration of (bi)linear forms, e.g., mappings from the reference to the physical space, etc., in cells and facets (for DG methods).
- The FE affine operator generated after the discretization of the original problem (probably after a linearization step). The FE solution is the only root (as soon as the problem is well-posed) of this operator. This operator, once the trial and test functions and the discrete (bi)linear forms of the problem at hand are defined, is represented through a matrix and a vector whose entries can be computed by numerical integration using the FE space.

FEMPAR has been used for more than 4 years now by a team of about 10 researchers of different research institutions and universities. During the initial OO re-design, derived data types (attributes and bindings) were gradually modified to accommodate new features that had not been considered, to fix expressivity limitations or even dependency knots of the original design. The software architecture to which we have converged, although certainly subject to future change, has been already proven to be capable to satisfy a number of users’ software requirements, even when the application problems involved complex and advanced features (e.g., the development of growing geometries in 3D printing technology). We consider that this steady regime, which has been attained after years of development, and a tremendous man-month power effort, is the proof that the software abstraction in FEMPAR is of practical relevance not only for prospective users and developers, but also for researchers that want to learn about the OO implementation of FE methods. It has motivated the decision of the authors to promote the library as a community software project, to open it to external users and new collaborators, to publish the library in an public git repository [42], and to write this article. In particular, the architecture described here corresponds to the first public release of FEMPAR, to which we assigned the git tag FEMPAR-1.0.0.

The first public release of FEMPAR has almost 300K lines of (mostly) Fortran code. Thus, a document like this one, with a quite detailed description of the services provided by the library and the motivation underlying our software design, can be a very valuable resource to complement the source code, which can become overwhelming in itself. In this paper, we have restricted ourselves to the construction of FE operators for body-fitted FE spaces. However, a major (and unique compared to other FE scientific software packages available on the Internet) cornerstone of FEMPAR is an abstract OO framework for the implementation of widely applicable highly scalable multilevel DD solvers.^40^ By letting this framework to be highly coupled with the numerical integration data structures of the application, on the one hand, and to be highly customizable, on the other, one can derive optimal preconditioners for the particular structure of the discrete operator at hand, and tackle new problems and challenges, while leveraging the distributed-memory implementation ideas [37] on which the framework is grounded on. Customizable building blocks in the framework include the fine-grid to coarse-grid DOFs aggregation, the constraint matrix underlying the imposition of continuity of coarse DOFs functionals across coarse objects, the weighting operator underlying the injection among the continuous and discontinuous spaces, and the kind of solvers to be used for the Dirichlet, Neumann constrained local problems, and the coarsest-grid global problem [103]. However, we postpone the discussion about solvers, preconditioners, data structures suitable for parallel distributed-memory computers, and other more exotic discretization techniques in FEMPAR, like B-splines and XFEM methods, to subsequent works.

## Footnotes

1 A paradigmatic example is the design of scalable solvers for the discretization of the Maxwell equations using edge elements, which involve the discretization of additional operators (discrete gradients) and changes of basis at the reference FE level [32].

2 Available at http://semver.org/.

3 The code snippets are written in advanced OO Fortran 200X [67]. There is a close relationship between these language features and those available in the C++ language [68] and we established some code style rules to emphasize it. In particular, Fortran modules in FEMPAR are always named with the suffix _names, to indicate the analogy with namespaces in C++. Derived types, analog to C structs or C++ classes, are always named with _t to distinguish them from instances. However it should be kept in mind that, whereas structs in C++ are passive data containers and classes are used to carry also methods, Fortran derived data types are used in both cases since the introduction in the 2003 standard of the so called type-bound procedures (TBPs).

4 In this section, we do not make difference between reference and physical spaces, e.g., using the ${\hat{\cdot }}$ symbol. In any case, all the following developments are usually performed at the reference FE level.

5 This assumption in fact applies for FEs of any kind, since the local functional spaces are already conforming and do not require an equivalence class at the cell level.

6 The test function spaces in the definition of the moments are always considered with respect to the corresponding domain of integration.

7 We note that we can take $\varvec{J}_{\hat{F}}^T \varvec{v}$ instead of $\varvec{J}_{\hat{F}}^T (\varvec{v}\times \varvec{n})$ in the definition of the face moments, since the rows of the Jacobian matrix are the transformation of the axes in the reference face ${\hat{F_0}}$ to the actual face ${\hat{F}}$ of the reference cell and the space of test functions is invariant to rotations.

8 We note that in fact the order *k* is not a scalar but a vector $\varvec{k} \in \mathbb {R}^d$. In principle, the use of a vector-valued order only has sense for n-cubes. The implementation in FEMPAR makes use of a vector-valued order, even though all entries should be the same for polytopes that are not n-cubes. We note that the use of different orders in different directions is basic to define high order Raviart-Thomas and Nédélec elements on n-cubes. In the following presentation, we consider the scalar order case for simplicity.

9 In the following, one can consider two unit cubes sharing a face. Since all the concepts are logical one does not have to take into account the real shape of the cells in the physical space. On the other hand, we note that the orientation index is invariant to which of the two cells sharing the face we select as first and second cell.

10 Analogously, one could generate serendipity elements only by changing the generation of the multi-dimensional space in terms of 1D ones.

11 We can consider three levels of DOF numbering: the cell-wise DOF numbering (referred to as local DOFs), the subdomain-wise DOF numbering (referred to as global DOFs), and a full domain global DOFs. The latter numbering is never created/required in FEMPAR. In serial environments, the latter two match.

12 We note that the responsibility of determining the permutation index does not lay on reference_fe_t, but on the abstraction of FEMPAR that represents the mesh of the computational domain; see Sect. 7.

13 This data type is implemented within the FPL software package [86].

14 We note that the actual conceptual representation of the triangulation in FEMPAR differences among local (to subdomain) cell identifiers and global cell identifiers (among the whole triangulation of the domain) in a distributed-memory context. The second sort of identifiers are coded as long precision integers, i.e., integer(igp), in order to accommodate simulations with more than $2^{31}-1$ global cells.

15 As mentioned in the case of cells, the actual conceptual representation of the triangulation in FEMPAR differences among local (to a subdomain) vef identifiers and global vef identifiers (among the whole triangulation of the domain) in a distributed-memory context. Again the latter ones are long precision integers.

16 For completeness, let us mention that triangulation_t also offers traversals over *subsets of objects* conveniently selected for acceleration purposes. For example, triangulation_t provides an iterator over vertices, edges, and faces that lay on the interface among subdomains, called itfc_vef_iterator_t (i.e., a subset of the set of objects traversed by vef_iterator_t) for those subclasses suitable for parallel distributed-memory environments.

17 Thus, e.g., a triangulation_t subclass that internally labels the global identifiers of vefs by their dimension in increasing order would result in a traversal with such an order. This is however a potentially changing over time low-level implementation detail that user programs relying on triangulation_t and its associated iterators should not assume nor rely on.

18 We note that $F^{3}_i \langle F^3 \rangle$ is simply $F^{3}_i$ and is not stored.

19 As it is well known, considering n-cube topologies for ${\hat{K}}$, for a Lagrangian reference FE of order *p* and an affine geometrical map, we need a 1D Gaussian quadrature with $p+1$ points. For tetrahedral meshes with the Duffy transformation, we need to take $n=p+ \mathrm{ceiling}(d/2)$ to integrate exactly mass matrices (see Sect. 3.5 for more details).

20 Here (and in many other places) we try to maximize the granularity of each call to a deferred binding for efficiency reasons. The reader should be aware that calling to deferred bindings with the granularity of the latter approach would be very expensive, apart from preventing a number of potential compiler optimizations enabled by the former.

21 This represents another design decision in the seek of maximizing the granularity of the calls to deferred bindings for code efficiency reasons.

22 In fact, 2D problems for PDEs that involve curl operators require SPACE_DIM to be equal to 3.

23 This can be easily fulfilled by calling the get_nodes_coordinates binding of vef_iterator_t in Listing 11.

24 We note that, as in Sect. 8.3, the nodes_coordinates(:) member variable of these two cell_map_t instances has to be loaded with the coordinates in physical space of the geometry nodes of the two cells surrounding the facet.

25 We stress, however, that the full set of services provided by fe_space_t is not actually restricted to only these two.

26 We note that fe_space_t is not actually in FEMPAR. It is a whole data type hierarchy rooted at base_fe_space_t, not included here for simplicity. Within this hierarchy, we have, e.g., FE space concretizations suitable for either serial or parallel distributed-memory environments. The one shown in the listing very much resembles serial_fe_space_t.

27 The term hash table here reflects its usual meaning, i.e., an associative array that maps keys to values.

28 We stress, however, that each particular reference_fe_t subclass at hand has the freedom to implement a different strategy if required.

29 We refer to Listing 43 and its accompanying text in Sect. 11 for a full description of the member variables and TBPs of block_layout_t. In this section, we restrict ourselves to those that are relevant for the global DOF numbering process.

30 The last requirement has been introduced to include the concept of void FEs for multi-field problems in which some fields are not defined on the whole domain (see Sect. 6.5).

31 We stress, however, that the approach discussed in the sequel to handle the strong imposition of boundary conditions is applicable to more complex problems and discretizations, e.g., the Maxwell equations discretized with curl-conforming FE spaces.

32 It is assumed that the discrete Dirichlet data extension is zero on free DOFs, but other more general situations can also be accommodated.

33 In parallel environments, every processor only stores the fixed DOF values that belong to its associated subdomain.

34 Some of the algorithms in charge of computing $E_h u_{\mathrm{D}}$ may require a different storage layout from the one of scalar_array_t (e.g., blocked storage and/or suitable for distributed-memory computers), and/or restrict themselves to those fixed DOFs of $E_h u_{\mathrm{D}}$ corresponding to a given field (or set of fields). In such a case, $E_h u_{\mathrm{D}}$ is scattered in place back and forth into temporary work space with the appropriate layout for the algorithm at hand in charge of computing its entries (e.g., a serial or parallel distributed-memory boundary mass problem iterative solver). It turns out that it is not such a high performance penalty provided that such algorithms already require to perform a sweep over boundary facets (e.g., in order to assemble a boundary mass matrix). During this sweep, the fixed DOFs in question can be already counted and identified.

35 Analytical scalar, vector, and tensor-valued functions are also supported in FEMPAR through the classes scalar_function_t, vector_function_t, and tensor_function_t, respectively. To implement an analytical scalar function $f(\varvec{x})$ in FEMPAR, the user has to extend scalar_function_t methods get_value, get_gradient (if used), etc., with the analytical expression, for a given $\texttt {point\_t}$ that represents $\varvec{x}$. We proceed analogously for vector and tensor fields. These data types are very simple and we omit their description here.

36 We note that the Stokes subclass of discrete_integration_t in Listing 41 implements the Galerkin approximation for this problem but it is independent of the FE space being used. It can be re-used for any conforming inf-sup stable mixed FE method, e.g., Taylor-Hood, conformal Crouzeix-Raviart, MINI element, etc. The choice of the mixed FE space will be determined by the user in the driver, when building the Cartesian two-field FE space.

37 The actual ordering of the fields in the Cartesian FE space is determined by the user in the creation of the multi-field FE space, which must be consistent with the implementation of the discrete weak form. See, e.g., the creation of the mixed Taylor-Hood FE space in Lines 11–21 of Listing 46, where the first field is the velocity field and the second one is the pressure field, and the integration of the weak form, e.g., in Lines 34, 37, and 42 of Listing 41, where this numbering is respected.

38 The block-wise numbering creates independently the DOF numbering of every block. Thus, DOFs of different blocks can have the same block-wise DOF label.

39 The pressure field belongs to $L^2(\Omega )$. Thus, a discontinuous pressure FE space could have been also considered as well. It would still be $L^2(\Omega )$-conforming. This is the case of, e.g., the conformal Crouzeix-Raviart mixed FE.

40 Indeed, the multilevel DD solvers within FEMPAR are since 2014 in the High-Q club of the most scalable European codes, maintained by the Jülich supercomputing center [102].
